# Terminal methylene phosphonium ions: precursors for transient monosubstituted phosphinocarbenes†

**DOI:** 10.1039/d3sc02899b

**Published:** 2023-06-29

**Authors:** Pawel Löwe, Marius A. Wünsche, Felix R. S. Purtscher, Jakob Gamper, Thomas S. Hofer, Lukas F. B. Wilm, Maike B. Röthel, Fabian Dielmann

**Affiliations:** a Instutut für Anorganische und Analytische Chemie Universität Münster Corrensstraße 28/30 48149 Münster Germany fabian.dielmann@uibk.ac.at; b Institute of General, Inorganic and Theoretical Chemistry Leopold-Franzens-Universität Innsbruck Innrain 80–82 6020 Innsbruck Austria

## Abstract

Isolable singlet carbenes are among the most important tools in chemistry, but generally require the interaction of two substituents with the electron deficient carbon atom. We herein report a synthetic approach to monosubstituted phosphinocarbenes *via* deprotonation of hitherto unknown diprotic terminal methylene phosphonium ions. Two methylene phosphonium salts bearing bulky *N*-heterocyclic imine substituents at the phosphorus atom were isolated and fully characterized. Deprotonation studies indicate the formation of transient monosubstituted carbenes that undergo intermolecular cycloadditions or intramolecular Buchner ring expansion to afford a cycloheptatriene derivative. The reaction mechanism of the latter transformation was elucidated using DFT calculations, which reveal the ambiphilic nature of the phosphinocarbene enabling the insertion into the aromatic C–C bond. Additional computational studies on the role of substituent effects are presented.

## Abstract 2

Diprotic terminal methylene phosphonium ions were prepared using bulky *N*-heterocyclic imine substituents. Deprotonation yields transient monosubstituted phosphinocarbenes, which insert into the C=C bond of a phenyl ring *via* a Buchner ring expansion.

## Graphical abstract

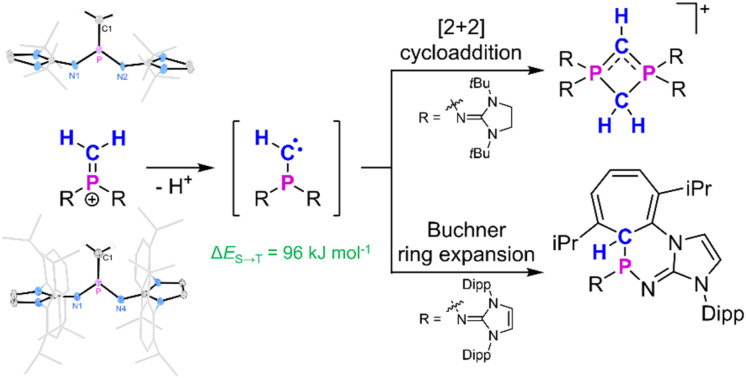

## Introduction

**Scheme 1:**
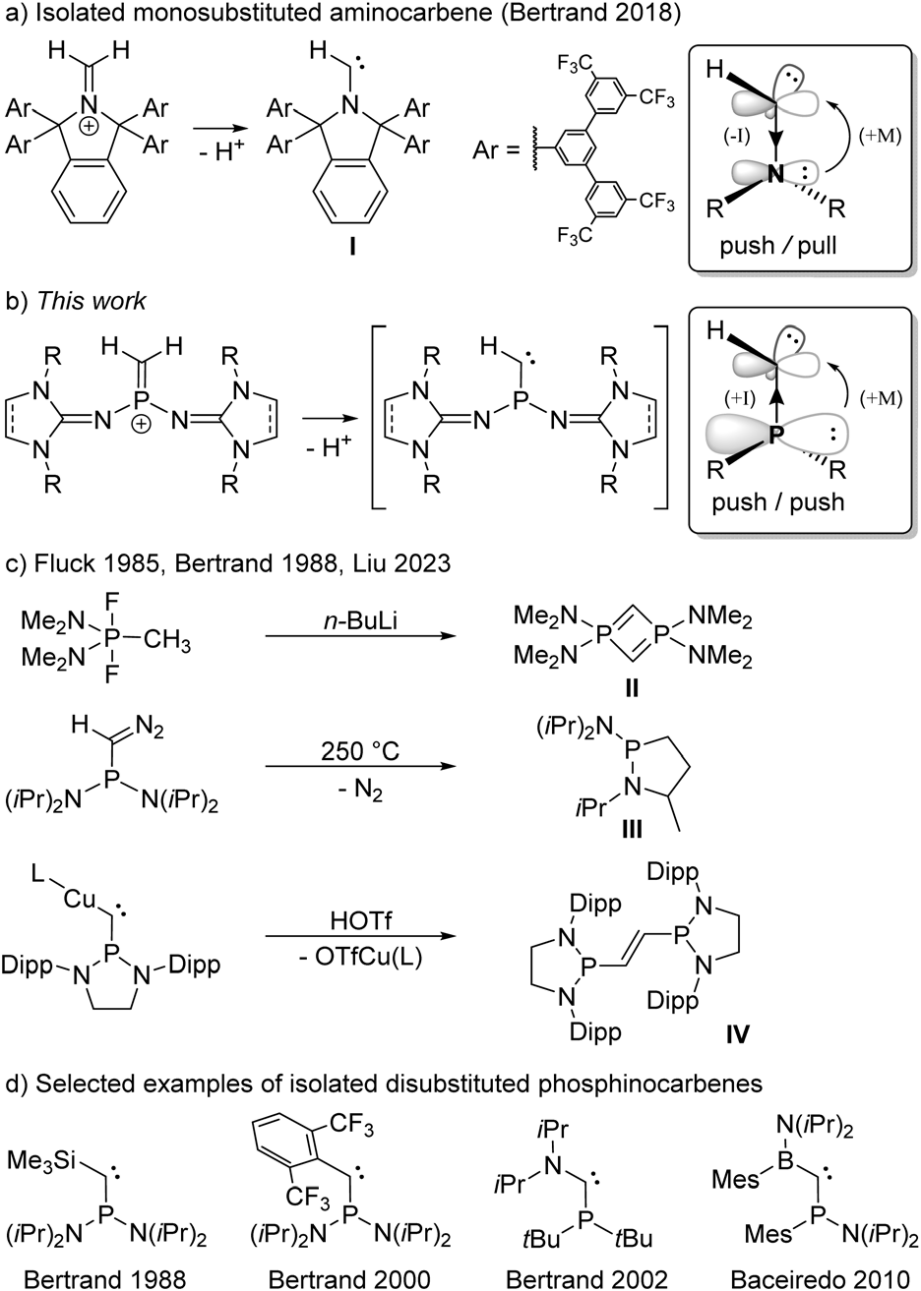
(a) Synthesis of a persistent monosubstituted aminocarbene reported by Bertrand. (b) Monosubstituted phosphinocarbenes transiently formed in this work. (c) Reactions in which phosphinocarbene intermediates have been postulated. (d) Selected examples of isolated phosphinocarbenes. L = 1,3-bis(2,6-diisopropylphenyl)imidazole-2-ylidene, Dipp = 2,6-diisopropylphenyl, OTf^−^ = trifluoromethanesulfonate (triflate). Boxes: Mesomeric and inductive substituent effects in amino- and phosphinocarbenes.

Carbenes are compounds of the general formula R_2_C featuring a divalent carbon atom with six electrons in its valence shell. While the parent carbene (H_2_C) has a triplet ground state and is too reactive to be isolated,^1^ it soon became evident that the carbene center is highly sensitive to electronic interactions with its substituents.^2–6^ Prompted by the pioneering work of Bertrand^7^ and Arduengo^8^ on the isolation of the first persistent singlet carbenes, countless stable carbenes with various substitution patterns have been prepared.^3,9–12^ Among the available heteroatom substituents, nitrogen substituents are particularly effective for the stabilization of carbenes, due to the interplay of π-donor and σ-acceptor properties.^13^ Indeed, *N*-heterocyclic carbenes (NHCs) – particularly diaminocarbenes – have evolved to be impressively useful tools in various fields of chemistry.^14–17^ Despite their significantly enhanced nucleophilicity and electrophilicity, several isolable NHCs bearing only one amino group and one alkyl or aryl substituent have been reported.^18,19^ Most remarkably, Bertrand and coworkers also succeeded in isolating the monosubstituted aminocarbene I by constructing an extremely bulky π-donor substituent to ensure its kinetic stabilization (Scheme 1a).^20^

This achievement raises the question of whether substituents other than amino groups are capable of stabilizing monosubstituted carbenes. The heavier phosphino group exerts much less π-conjugation towards the carbon center than an amino group, leading to smaller singlet–triplet energy separation and a low-lying LUMO,^21,22^ which results in an increased electrophilicity and nucleophilicity of the carbene center. The interaction of the carbene carbon atom with a second substituent was therefore hitherto required for the formation of stable phosphinocarbenes (Scheme 1d).^3,23–26^ The intermediate existence of a monosubstituted phosphinocarbene was speculated by Fluck and coworkers in 1985, when they treated (Me_2_N)_2_PF_2_CH_3_ with 2 equivalents of *n*-butyllithium and obtained diphosphete II as the formal carbene dimer (Scheme 1c).^27,28^ Later, Bertrand and coworkers showed that thermolysis of (iPr_2_N)_2_PC(N_2_)H at 250 °C leads to heterocycle III, which formally results from a C–H insertion reaction of the corresponding phosphinocarbene (Scheme 1c).^7,29^ Very recently, Liu and coworkers demonstrated that protonation of the bulky copper phosphinocarbyne anion complex IV results in the formation of the corresponding R_2_P–CH=CH–PR_2_ carbene dimer.^30^ We reasoned that deprotonation of terminal methylene phosphonium ions would provide access to monosubstituted phosphinocarbenes under mild conditions. Although several methylene phosphonium salts have been reported, mainly by the groups of Appel,^31^ Bertrand,^32^ Grützmacher,^33–35^ Erker,^36^ and Baceiredo,^24^ stabilizing groups at both phosphorus and carbon were employed in all cases. Herein, we report a synthetic access to terminal methylene phosphonium cations and show that they are suitable precursors for transient monosubstituted phosphinocarbenes.

## Results and discussion

### Experimental results

**Scheme 2:**
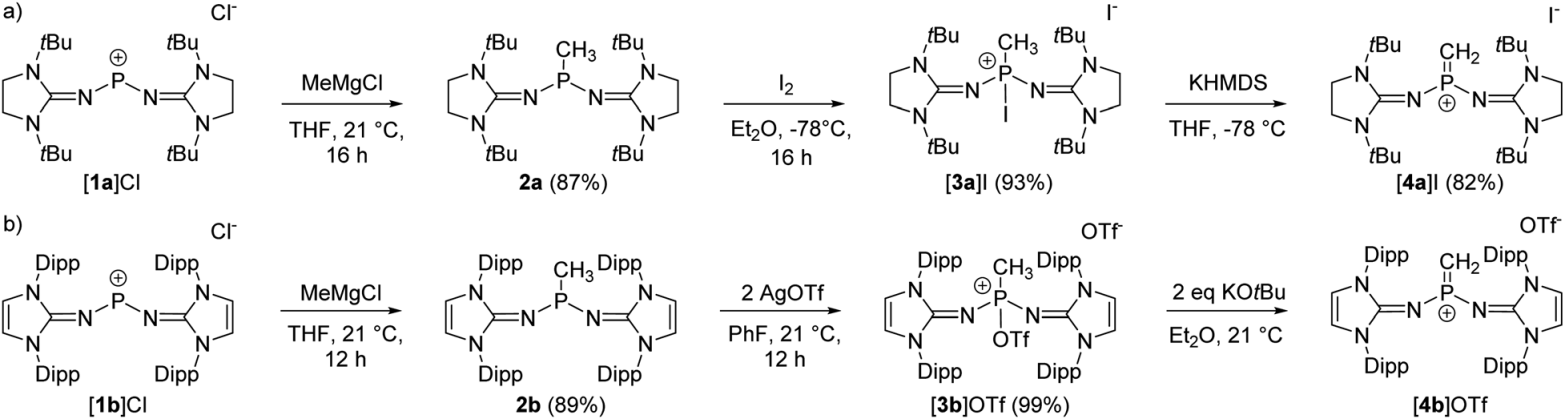
Synthesis of methylene phosphonium salts [4a]I (a) and [4b]OTf (b).

**Fig. 1:**
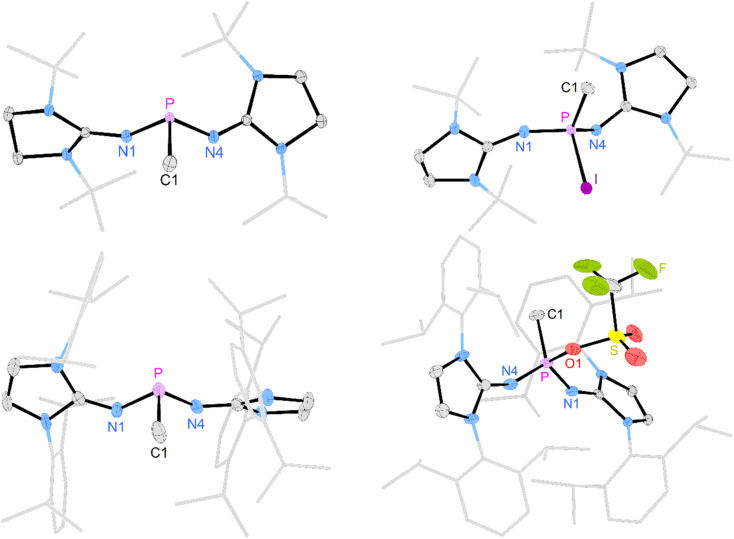
Solid-state structures of 2a (top left), 2b (bottom left), [3a]I (top right), and [3b]OTf (bottom right). In 2b and [3b]OTf, only one of the two crystallographically independent molecules is depicted. Hydrogen atoms, anions, and disordered moieties are omitted for clarity. Ellipsoids are drawn at 50% probability. *t*Bu (2a, [3a]^+^) and Dipp (2b, [3b]^+^) groups are shown in wireframe. Selected bond lengths [Å] and angles [deg]: 2a: P–C1 1.8444(9), P–N1 1.6700(8), P–N4 1.6824(8), C1–P–N1 97.88(4), C1–P–N4 97.03(4), N1–P–N4 100.23(4). [3a]I: P–C1 1.785(2), P–N1 1.550(3), P–N4 1.570(2), P–I 2.4982(8), C1–P–N1 113.20(13), C1–P–N4 109.75(12), N1–P–N4 114.02(12), C1–P–I 99.10(9). 2b: P–C1 1.848(5), P–N1 1.702(4), P–N4 1.687(4), C1–P–N1 95.6(2), C1–P–N4 103.3(2), N1–P–N4 96.6(2). [3b]OTf: P–C1 1.787(5), P–N1 1.543(4), P–N4 1.549(4), P–O1 1.730(4), C1–P–N1 117.9(2), C1–P–N4 115.1(2), N1–P–N4 110.8(2), C1–P–O1 99.3(2).

The synthesis of the terminal diprotic methylene phosphonium ions [4a]^+^ and [4b]^+^ was realized by oxidation of the methyl-substituted phosphines to phosphonium salts and subsequent deprotonation, analogous to the original synthesis by Grützmacher (Scheme 2).^37^ Sterically demanding *N*-heterocyclic imines (NHIs) have been shown to be effective for the kinetic stabilization of low-coordinate phosphorus(v),^25,38–42^ and other main group cations.^43^ Moreover, Schoeller predicted that nitrogen-based π-donor substituents at phosphorus effectively increase the singlet–triplet gap of phosphinocarbenes.^21^ Hence, we chose NHIs with *tert*-butyl (*t*Bu) and 2,6-diisopropylphenyl (Dipp) groups to flank the reactive P=C double bond. The phosphenium salts [1a]Cl and [1b]Cl (Scheme 2) were prepared according to reported procedures by the reaction of PCl_3_ with NHI–SiMe_3_ derivatives upon elimination of ClSiMe_3_.^25,44–46^ Alkylation of the phosphenium cations with MeMgCl gave phosphines 2a and 2b as crystalline solids in very good yields. Characteristically, the phosphines show quartets (2a: 69.3 ppm, 2b: 62.0 ppm) in the ^31^P NMR spectrum with ^2^*J*_PH_ coupling constants of 7–9 Hz. The solid-state structures of the bulky phosphines were determined by single-crystal X-ray diffraction (SCXRD) studies showing the expected pyramidalization of the phosphorus atoms (Fig. 1, left).

The oxidation of the electron-rich phosphines 2a and 2b was carried out with elemental iodine and silver trifluoromethanesulfonate (AgOTf), respectively, to afford the corresponding phosphonium salts [3a]I and [3b]OTf as white, air-sensitive solids in excellent yield. The use of elemental iodine allows the subsequent exchange of the iodide for a weakly coordinating anion (*vide infra*), but entails the problem that the triiodide anion is formed with excess iodine, as confirmed by a SCXRD analysis of [3a]I_3_ (see the SI for details). AgOTf was therefore used as a more convenient oxidizing agent in the case of the sterically encumbered phosphine 2b. SCXRD studies reveal distorted tetrahedral bonding environments for the phosphonium cations (Fig. 1, right). The P–O bond in [3b]OTf (1.730(4) Å) is exceptionally long compared to other P^V^–O bonds (*cf.* Ph_3_P–O: 1.479(2) Å;^47^ [Ph_3_P–O–PPh_3_]^2+^: 1.597(3) Å,^48^ 1.598(4) Å; [{(Ph_3_P)_2_C}P(OTf)Ph_2_]^2+^: 1.676(4) Å).^49^ This elongation and the planarization of the N_2_PC moiety (sum of angles [3b]^+^: 343.8°) suggests that the P–O bond is strongly activated, which is supported by the observation that [3b]OTf readily polymerizes tetrahydrofuran (THF) in solution.

The solid-state structure of [3a]I also exhibits a distorted tetrahedral geometry of the phosphorus atom. However, the N_2_PC unit of [3a]^+^ is less planarized (sum of angles: 337°) compared with that of [3b]^+^. There are short contacts of 3.829 Å between the phosphorus bound iodine and the iodide anion, which is not uncommon for iodophosphonium iodide salts including [iPr_3_I]I (3.384 Å)^50^ and [(Me_2_N)_3_PI]I (3.619 Å)^51^ (van der Waals radii for two iodine atoms: 4.3 Å).^52^ The ^31^P NMR resonances of [3a]I (−71.7 ppm) and [3b]OTf (−11.9 ppm) appear as quartets with ^2^*J*_PH_ coupling constants of 14–16 Hz. Consistent with the solid-state structures, two distinct ^19^F NMR resonances are observed in solution for the phosphorus bound triflate and the free anion, respectively (−73.67 ppm and −78.77 ppm). The ^127^I NMR spectrum of [3a]I shows a broad signal at 62 ppm, which is in the expected range for free iodide.^53^ The absence of a second signal corresponding to the phosphorus-bound iodine atom is due to the quadrupolar nature (*I* = 5/2) of the ^127^I nucleus,^54^ since resonances with sufficiently narrow line width are only detected for iodine in highly symmetrical environments.^55,56^

**Fig. 2:**
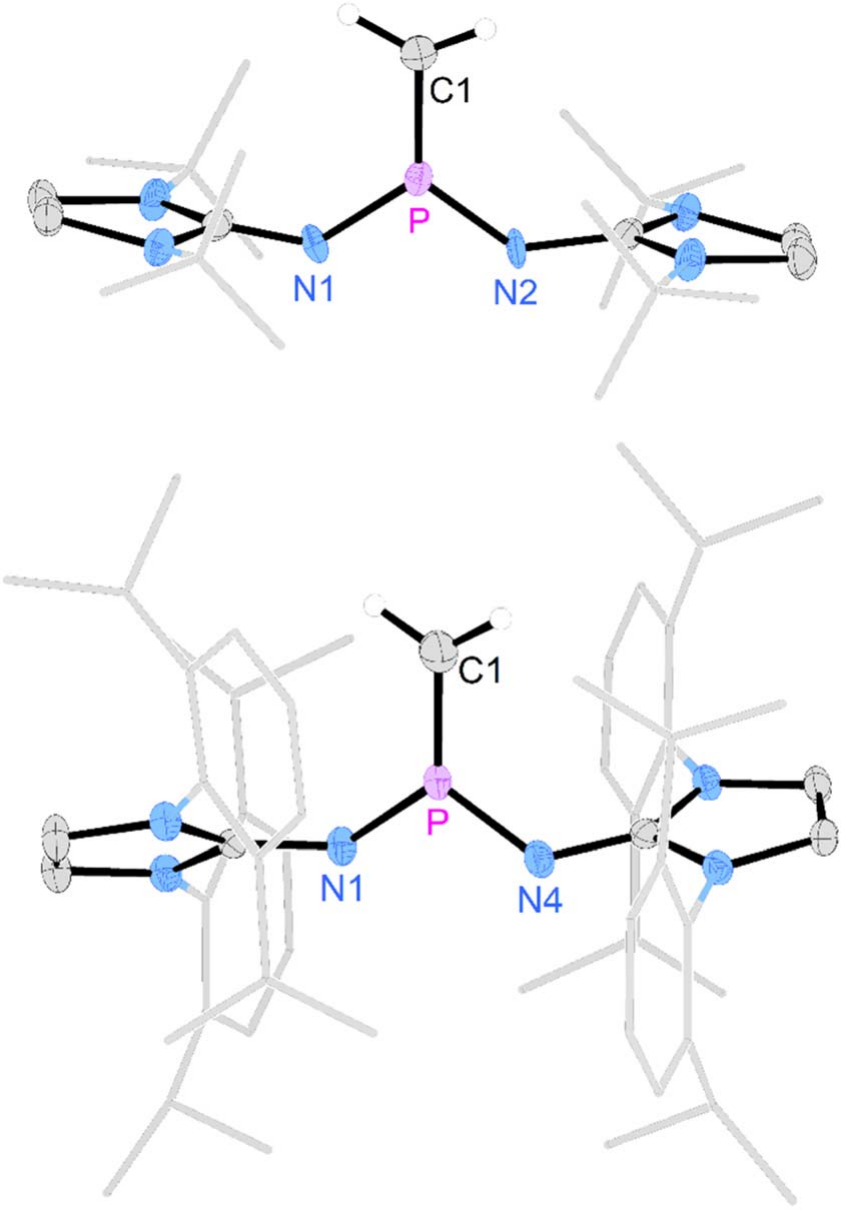
Solid-state structure of [4a]I (top) and [4b]OTf (bottom). Hydrogen atoms (except C1 hydrogen atoms) and anions are omitted for clarity. Ellipsoids are drawn at 50% probability. *t*Bu ([4a]^+^) and Dipp ([4b]^+^) groups are shown in wireframe. Only one of two crystallographically independent molecules of [4b]OTf is depicted. Selected bond lengths [Å] and angles [deg]: [4a]I: P–C1 1.621(5), P–N1 1.569(7), P–N2 1.523(9), C1–P–N1 123.7(4), C1–P–N2 125.9(3), N1–P–N2 110.4(5). [4b]OTf: P–C1 1.619(3), P–N1 1.566(2), P–N4 1.565(2), C1–P–N1 125.9(2), C1–P–N4 123.29(13), N1–P–N4 110.69(9).

Deprotonation of [3a]I with potassium hexamethyldisilazide (KHMDS) resulted in the formation of the desired terminal methylene phosphonium salt [4a]I, which was obtained after recrystallization as large colorless crystals in 82% yield. [4a]I is air sensitive but can be stored under inert atmosphere for months without noticeable decomposition. It is soluble in dichloromethane but sparingly soluble in THF or Et_2_O and decomposes in acetonitrile. Exposure to D_2_O leads to activation of both O–D bonds and affords [(NHI)_2_CH_2_DP–O–PCH_2_D(NHI)_2_]^2+^ (NHI = 1,3-di-*tert*-butylimidazolidin-2-ylidenamino), which is isotypic to the Hendrickson reagent [Ph_3_POPPh_3_]^2+^ (see the SI for details). The methylene phosphonium salt [4a]I shows a characteristic triplet at 89 ppm (^2^*J*_PH_ = 15 Hz) in the ^31^P NMR spectrum. The ^13^C resonance of the terminal methylene group (32.2 ppm, ^1^*J*_CP_ = 186 Hz) exhibits a significantly larger ^1^*J*_PC_ coupling constant than [3a]I (34.3 ppm, ^1^*J*_CP_ = 119 Hz). The broad signal at 77 ppm in the ^127^I NMR spectrum indicates that iodide is not bound to the cationic molecule.^53^ A SCXRD study confirms the ionic nature of [4a]I in the solid state with the iodine atom located 5.52 Å away from the phosphorus center (Fig. 2, top). The P atom is in a trigonal planar environment (sum of angles: 360°). The P–C bond length (1.620 Å) is slightly shorter than in the methylene phosphonium ions reported by Bacereido [Mes(iPr_2_N)P=CH(BMes(NiPr_2_))]^+^ (1.634 Å)^24^ and Grützmacher [*t*Bu_2_P = CPh(*o*-MeC_6_H_4_)]^+^ (1.680 Å),^35^ consistent with the minimum steric bulk at the methylene moiety.

The deprotonation of the sterically encumbered phosphonium salt [3b]OTf turned out to be unselective regardless of the type of base. Treatment with lithium organyls such as MeLi, *n*BuLi and PhLi led to the reduction to the phosphine 2b in more than 70% yield according to ^31^P NMR analysis. The fact that the lithium organyls act as reducing agents^57,58^ rather than as bases can be attributed to the effective steric shielding of the methyl protons by the Dipp substituents, which is also evident in the solid-state structure of [3b]OTf (see Fig. S97 and S98† for steric maps of [3a]^+^ and [3b]^+^). NBO charges of 1.842*e* and 2.238*e* were calculated for the phosphorus atoms of [3a]^+^ and [3b]^+^, respectively, making an electron transfer to the electrophilic P atom a plausible alternative pathway when deprotonation is hampered by bulky substituents. The attempted deprotonation of [3b]OTf with stoichiometric amounts of KHMDS or KO*t*Bu did not afford the reduced phosphine 2b, but gave several phosphorus species. However, when using two equivalents of KO*t*Bu, the ^31^P NMR spectrum of the reaction mixture showed the characteristic triplet of the methylene phosphonium cation at 102.6 ppm (^2^*J*_PH_ = 13 Hz) as the major species along with a resonance at 0.0 ppm, which we putatively assign to the phosphine oxide.^59,60^ The formation of the latter can be explained by a nucleophilic attack of the base at the sulfur atom of the triflate. A detailed study on the reactivity of the triflate-containing phosphonium ion [{(Ph_3_P)_2_C}P(OTf)Ph_2_]^2+^ with nucleophiles by Weigand and co-workers showed that both the P atom and the S atom are prone to nucleophilic attack.^49^ Substitution at the latter leads to the elimination of the phosphine oxide. Although we were not able to isolate [4b]OTf on preparative scale, a SCXRD of a single crystal grown from the reaction mixture confirmed its molecular structure, which exhibits geometric parameters analogous to [4a]I (Fig. 2, bottom).

**Scheme 3:**
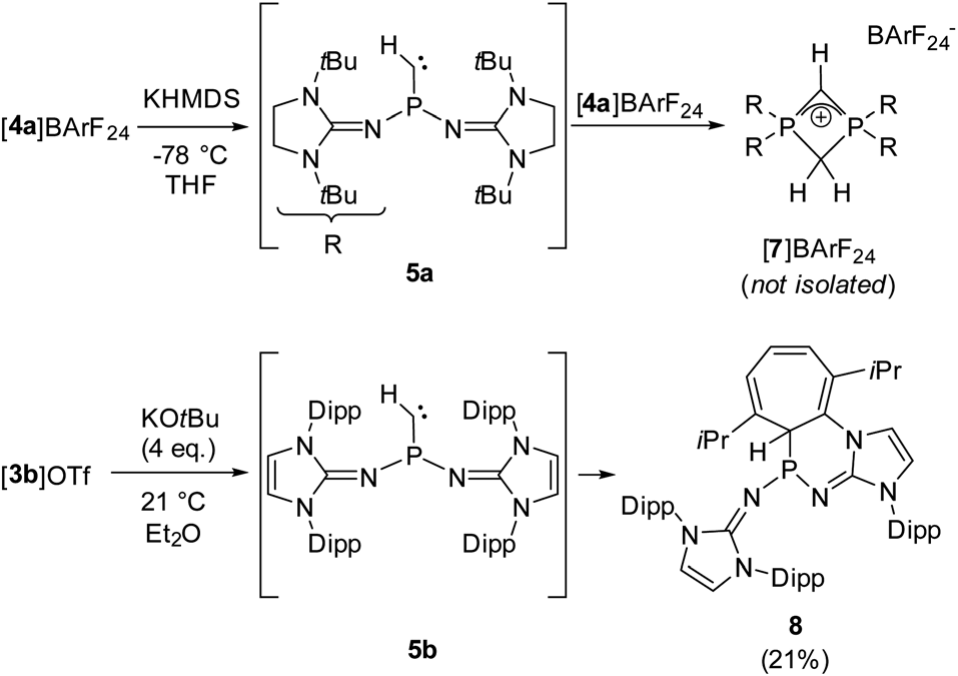
Reactivity of [4a]BArF_24_ with KHMDS and reactivity of [3b]OTf with an excess of KO*t*Bu.

We next performed deprotonation studies to generate the desired monosubstituted phosphinocarbene (Scheme 3). For this purpose, [4a]BArF_24_ (BArF_24_ = [2,6-bis(trifluoromethyl)phenyl]borate) was prepared from the reaction of [4a]I with Na(BArF_24_) to increase the solubility of the methylene phosphonium ion in ether solvents. Treatment of [4a]BArF_24_ with various bases gave the diphosphete salt [7]BArF_24_ as the major product (>80% yield using KHMDS) along with other unidentified products (see the ESI† for details), which leads to the conclusion that the free carbene reacts readily with the methylene phosphonium precursor. The composition of [7]BArF_24_ was confirmed by high resolution mass spectrometry and it was characterized using multinuclear 1D and 2D NMR spectroscopy (see the SI for details). The ^31^P resonance at −39.7 ppm is split into a triplet (^2^*J*_PH_ = 18 Hz) due to coupling with the CH_2_ ring protons. In agreement with the isostructural phosphete ion [(NMe_2_)_2_PCH_2_CHP(NMe_2_)_2_]^+^, the ^2^*J*_PH_ coupling constant with the CH bridge is not resolved.^61^ In the ^13^C{^1^H} NMR spectrum of [7]BArF_24_, characteristic triplets are observed for both the CH bridge (63.1 ppm, ^1^*J*_CP_ = 134 Hz) and the CH_2_ bridge (51.7 ppm, ^1^*J*_CP_ = 84 Hz).

Aiming at intercepting the generated phosphinocarbene, the deprotonation of [4a]BArF_24_ was performed in the presence of various trapping reagents including cyclohexene, diphenylacetylene, 1-propynylbenzene, elemental selenium and [Rh(COD)Cl]_2_ (COD = 1,5-cyclooctadiene). However, the formation of [7]BArF_24_ did not appear to be affected by the trapping reagents, which we attribute to the fact that both 5a and [4a]BArF_24_ are highly reactive. In addition, the basic carbene atom is expected to bind to Lewis acids after deprotonation, which hampers carbene-type reactivity but does not prevent the adjacent phosphino group from initiating the cyclization with the electrophilic methylene phosphonium cation.

**Fig. 3:**
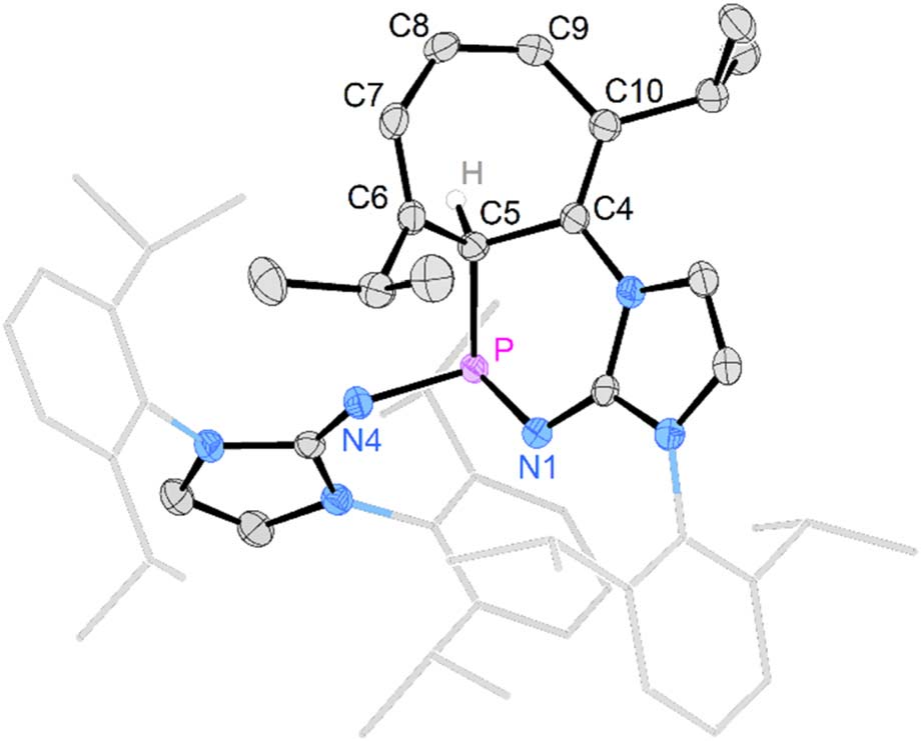
Solid-state structure of 8. Hydrogen atoms (except C5 hydrogen) are omitted for clarity. Ellipsoids are drawn at 50% probability. Dipp groups are shown in wireframe. Selected bond lengths [Å] and angles [deg]: P–C5 1.866(2), P–N1 1.7017(14), P–N4 1.693(2), C5–C6 1.510(3), C6–C7 1.358(2), C7–C8 1.433(3), C8–C9 1.366(3), C9–C10 1.438(3), C10–C4 1.366(2) C4–C5 1.510(3), N1–P–N4 199.67(7), N1–P–C5 103.78(7), C4–P–C5 100.84(7).

Gratifyingly, unequivocal evidence for the formation of the transient phosphinocarbene was provided by deprotonation of [3b]OTf using an excess of KO*t*Bu, which gave the formal ring insertion product 8 (Scheme 3). ^31^P NMR analysis of the reaction mixture indicates 62% conversion to the polycyclic phosphine 8 (Fig. S79†), which was isolated after extraction with *n*-hexane and recrystallization as yellow crystalline solid in 21% yield. Phosphine 8 shows a diagnostic doublet at 73.2 ppm in the ^31^P NMR spectrum with a ^2^*J*_PH_ coupling constant of 11 Hz. The molecular structure was established by a SCXRD study and reveals that the CH carbene moiety was inserted into a C=C bond of a former Dipp substituent (Fig. 3). The cycloheptatriene ring of the resulting tricyclic structure shows alternating C=C double bonds (C4–C10 1.366 Å, C6–C7 1.358 Å, C8–C9 1.366 Å) and C–C single bonds (C4–C5 1.510 Å, C5–C6 1.510 Å, C7–C8 1.433 Å, C9–C10 1.438 Å).

The formation of compound 8 is rationalized by the intermediate generation of the phosphinocarbene 5b which undergoes an intramolecular cyclopropanation with an adjacent Dipp substituent followed by a divinylcyclopropane–cycloheptadiene rearrangement. The analogous Buchner ring expansion reactions, involving benzene derivatives and transient carbenes or complexes thereof, has been widely used to introduce cycloheptatriene rings into natural products and materials.^62–69^ Similar intramolecular ring expansions were reported for other transient main-group carbene analogues, such as silylenes,^70–76^ and phosphinidenes.^77^ Attempts to trap the transient carbene by performing the deprotonation at −40 °C in fluorobenzene in the presence of 2 bar dihydrogen or carbon monoxide, both known to react with electrophilic carbenes,^78–80^ were unsuccessful.

### Computational studies

**Fig. 4:**
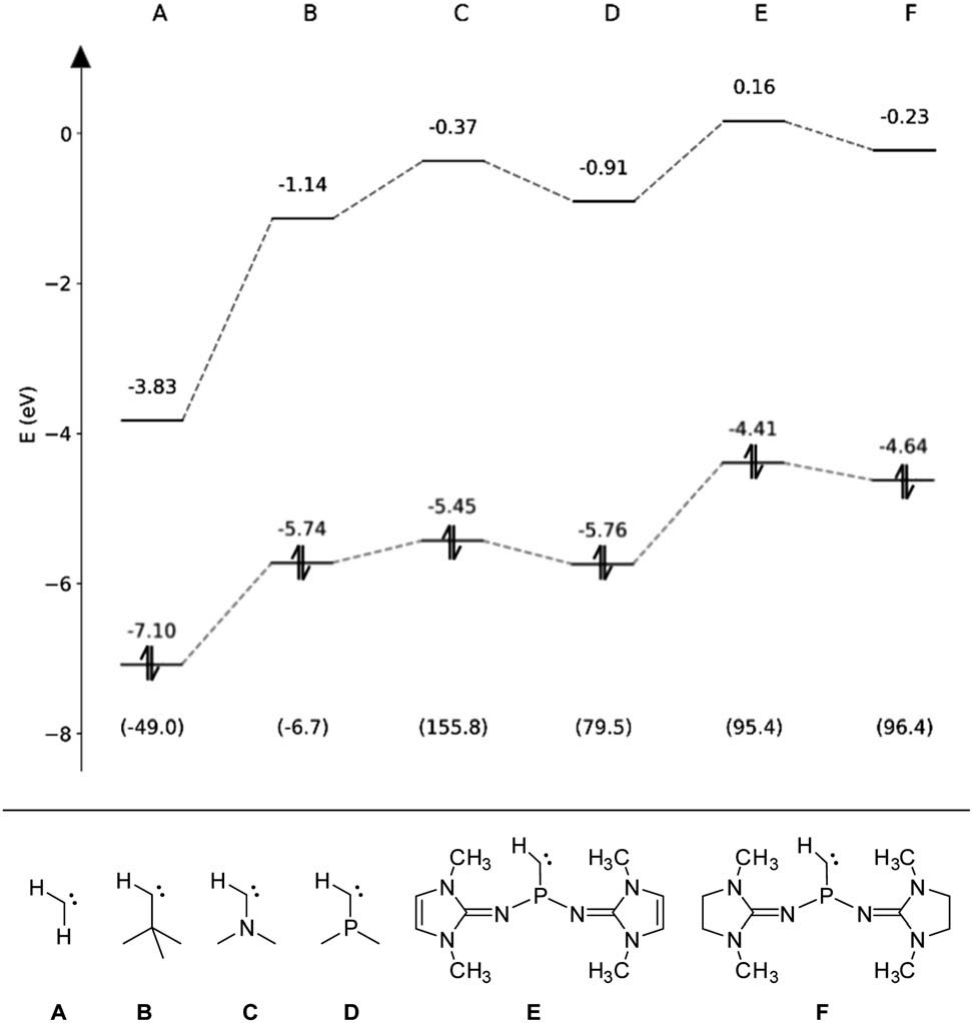
Eigenenergies of the HOMO and LUMO for carbenes A–F obtained at the B3LYP-GD3BJ/def2-TZVP level. The respective singlet–triplet energy differences (Δ*E*_ST_) are given in kJ mol^−1^ in parentheses.

To assess the electronic properties of the transient phosphinocarbene and elucidate the mechanism of the ring insertion reaction, theoretical calculations at the B3LYP-GD3BJ/def2-TZVP level of theory^81–83^ were performed. The HOMO/LUMO energies in the respective singlet state along with the associated singlet–triplet energy difference (Δ*E*_ST_) have been calculated for simplified model compounds (Fig. 4, E and F) and compared to prototype aliphatic and heteroatom-substituted (N, P) carbenes (Fig. 4, A–D). While the non-heteroatom substituted carbenes A and B are predicted to have a triplet ground state, the singlet state is energetically favored in case of the heteroatom substituted carbenes (C–F) in agreement with previous studies.^84,85^ Furthermore, substitution of the nitrogen atom in compound C by a phosphorus atom (D) leads to a much smaller Δ*E*_ST_. Although the gap increases by the employment of NHI substituents (E, F), it is still much lower compared to the ones from NHCs and CAACs (>200 kJ mol^−1^).^86,87^ Moreover, the calculated Δ*E*_ST_ of E and F are lower than that of the acyclic amino(alkyl)carbene iPr_2_N–C–*t*Bu (112.5 kJ mol^−1^)^20^ but slightly higher than that of the persistent amino(silyl)carbene iPr_2_N–C–SiPh_2_*t*Bu (84.9 kJ mol^−1^).^88^ Among the carbenes considered, E and F show the highest HOMO energy, in the same range like iPr_2_N–C–*t*Bu (−4.69 eV),^20^ indicating that they are stronger σ-donors compared to carbenes A–D. In contrast, the HOMO–LUMO gap of E and F (4.57 eV and 4.87 eV) is comparable to the other monosubstituted carbenes B–D.

**Fig. 5:**
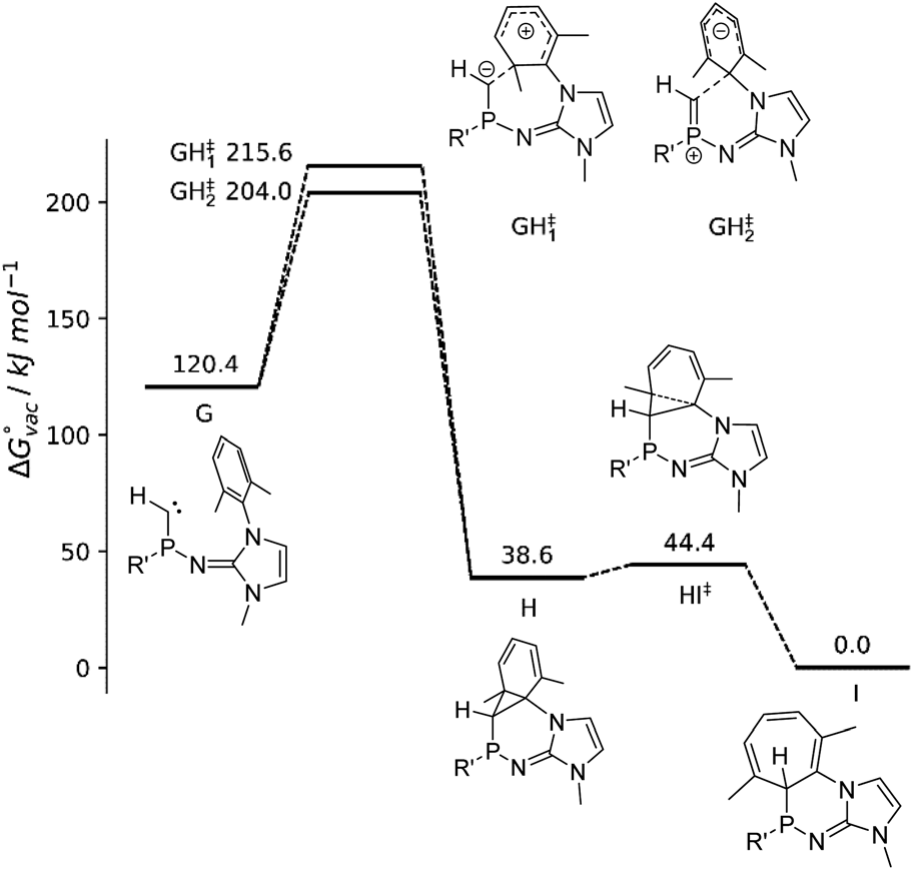
Free energy profile of the reaction path G → I including the identified transition states GH^‡^_1–2_ and HI^‡^ obtained at B3LYP-GD3BJ/def2-TZVP level of theory in the gas phase. R′ = 1,3-dimethylimidazolin-2-ylidenamino.

In addition, the reaction path of the ring insertion of the phosphinocarbene has been characterized for a simplified model system (Fig. 5, G) by DFT calculations, considering both a vacuum environment and implicit solvation in diethyl ether. Although the targeted analog of model compound G could not be isolated experimentally, the theoretical calculations confirm the proposed reaction mechanism of a carbene formation and subsequent ring insertion *via* an initial [1 + 2] cycloaddition reaction step in both vacuum as well as implicit solvation. Two different transition states for the reaction G → H could be identified, which display a comparable activation energy. However, close inspection of the associated P–C_carbene_ bond distance *r*_PC_ and the angular sum around the P atom reveal that the respective reactions are fundamentally different: Phosphinocarbene G exhibits a P=C double bond of about 1.61 Å and a planar P atom (sum of angles: 359°). While this structural pattern is very well retained for transition state GH^‡^_2_, the bond distance and angular sum changed to 1.69 Å and 330° in case of GH^‡^_1_. The latter implies a conversion of the double bond towards a P–C single bond along with the pyramidalization of the P atom, which is associated with an electrophilic addition of the carbene to the phenyl ring. On the other hand, the unchanged geometry observed for GH^‡^_2_ implies a nucleophilic attack of the carbene at the aromatic moiety.

From the intermediate state H onward, the geometrical properties correspond to that of a P–C single bond in the range of 1.85 to 1.86 Å with the corresponding angular sums being close to 300° as expected for NHI-substituted phosphines (2b: 295°).

The isolation of the intermediate H appears not possible due to the small activation barrier of 9.7 kJ mol^−1^ and 9.2 kJ mol^−1^ in vacuum and implicit solvent, respectively, corresponding to an effectively instantaneous reaction to the final product I. The reaction barriers associated with the two transitions states GH^‡^_1_ and GH^‡^_2_ are in the range of 83.6 to 95.2 kJ mol^−1^, which implies that the phosphinocarbene should be sufficiently long-lived to be trapped by a suitable reagent. However, the basic phosphinocarbene is expected to interact with Lewis acids such as K^+^ in the reaction mixture, which might significantly facilitate the electrophilic addition mechanism. Similarly, the electrophilic addition of a transient phosphinidene to a mesityl ring was promoted by its coordination to AuCl.^77^

## Conclusions

We report the synthesis and full characterization of the first terminal methylene phosphonium ions and demonstrate that they are suitable precursors for the generation of monosubstituted phosphinocarbenes. The latter are highly reactive and, depending on the steric bulk of the NHI substituents, either undergo intermolecular [2 + 2] cycloadditions to form P_2_C_2_ four-membered rings or insert intramolecularly into the C=C bond of an adjacent aryl substituent, leading to a Buchner ring expansion and the formation of a cycloheptatriene derivative. DFT calculations reveal similar reaction barriers for both the electrophilic and the nucleophilic attack at the phenyl ring, corroborating the ambiphilic character of the phosphinocarbene. Although we were not able to isolate the phosphinocarbenes, the present study shows that NHI substituents effectively increase the singlet triplet energy gap. Thus, the challenge in designing isolable monosubstituted phosphinocarbenes appears to narrow down to the design of appropriate substituents that can withstand the ambiphilic carbon center, which is the subject of ongoing research in our laboratories.

## Data availability

Further details of the experimental procedures, the computational studies, and the charactization data for the new compounds are available in the ESI.†

## Author contributions

P. L. and M. A. W. synthesized the compounds and performed the experimental studies. M. B. R. helped with experiments and obtained characterization data. P. L., L. F. B. W. and F. D. performed the SCXRD studies. F. R. S. P., J. G and T. S. H. performed the computational studies. P. L., T. S. H. and F. D. wrote the manuscript. F. D. directed the investigation. All authors have given approval to the final version of the manuscript.

## Conflicts of interest

There are no conflicts to declare.

## Supplementary Material

SC-014-D3SC02899B-s001

SC-014-D3SC02899B-s002
