# Supplementary material for: Terminal methylene phosphonium ions: precursors for transient monosubstituted phosphinocarbenes
Source: Chem Sci. 2023 Jun 29;14(29):7928–35. doi: 10.1039/d3sc02899b (PMC10370551; doi:10.1039/d3sc02899b)
Supplement: SC-014-D3SC02899B-s001 [file SC-014-D3SC02899B-s001.pdf]

Electronic Supplementary Material (ESI) for Chemical Science.
This journal is © The Royal Society of Chemistry 2023

Supplementary Material for

Terminal methylene phosphonium ions: precursors for transient monosubstituted phosphinocarbenes

Pawel Löwe,[a] Marius A. Wünsche,[a] Felix R. S. Purtscher,[b] Jakob Gamper,[b] Thomas S. Hofer,[b] Lukas F. B. Wilm,[a] Maike B. Röthel,[b] Fabian Dielmann[a,b]*

a Institut für Anorganische und Analytische Chemie, Westfälische Wilhelms-Universität Münster, Corrensstraße 28/30, 48149 Münster (Germany)

b Institute of General, Inorganic and Theoretical Chemistry, Universität Innsbruck, Innrain 80-82, 6020 Innsbruck (Austria), E-mail: Fabian.Dielmann@uibk.ac.at

CONTENTS:

1 **Experimental procedures** 3

1.1 Synthetic Details 3
1.2 Preparation of [**1b**]Cl 4
1.3 Preparation of **2a** 9
1.4 Preparation of **2b** 13
1.5 Preparation of [**3a**]I 16
1.6 Characterization data of [**3a**]I_3 20
1.7 Preparation of [**3b**]OTf 23
1.8 Preparation of [**4a**]I 26
1.9 Preparation of [**4a**]$BArF_{24}$ 32
1.10 Preparation of [**4a'**]$(BArF_{24})_2$ 38
1.11 Characterization data of [**4a'***]$(BArF_{24})_2$ 42
1.12 Crude ^{31}P NMR spectrum containing [**4b**]OTf 47
1.13 Characterization data of [**7**]$BArF_{24}$ 48
1.14 Deprotonation study of [**4a**]$BArF_{24}$ 54
1.15 Preparation of **8** 55

2 **X-ray Diffraction Studies** 60

2.1 Crystal structure data of compound **2a** 61
2.2 Crystal structure data of compound **2b** 62
2.3 Crystal structure data of compound [**3a**]I 63
2.4 Crystal structure data of compound [**3a**]I_3 64
2.5 Crystal structure data of compound [**3b**]OTf 65
2.6 Crystal structure data of compound [**4a**]I 66
2.7 Crystal structure data of compound [**4a'**]$(BArF_{24})_2$ 67
2.8 Crystal structure data of compound [**4b**]OTf 68
2.9 Crystal structure data of compound **8** 69

3 **Computational studies** 70

3.1 General 70
3.2 Optimized structures of structures **G-I** 70
3.3 Cartesian Coordinates 72
3.4 Steric maps of [**3a**]$^+$ and [**3b**]$^+$ 82
3.5 Atomic charges and electrostatic surface potential plots of model compounds of phosphonium cations [**3a**]$^+$, [**3b**]$^+$ and [**4a**]$^+$/[**4b**]$^+$ 83

4 **References** 88

1 Experimental procedures

1.1 Synthetic Details

General remarks: All manipulations were performed under an inert atmosphere of dry argon, using standard Schlenk and drybox techniques. Dry and oxygen-free solvents were employed. All glassware was oven-dried at 160 °C prior to use. ^{1}H, ^{13}C, ^{31}P, ^{35}Cl and ^{127}I NMR spectra were recorded at 300 K on Agilent DD2 600, Bruker AVANCE I 400, Bruker AVANCE III 400 or Bruker AVANCE II 200 spectrometers. Chemical shifts (δ) are given in parts per million (ppm) relative to $SiMe_4$ (^{1}H, ^{13}C), 85% H_3PO_4 (^{31}P), 0.1 M NaCl in D_2O (^{35}Cl), 0.01 M KI in D_2O (^{127}I) and referenced to the residual solvent signals ($CDCl_3$: ^{1}H δ_H = 7.26, ^{13}C δ_C = 77.16; CD_2Cl_2: ^{1}H δ_H = 5.32, ^{13}C δ_C = 54.00; C_6D_6: ^{1}H δ_H = 7.16, ^{13}C δ_C = 128.06; CD_3CN: δ_H = 1.94, ^{13}C δ_C = 118.26) or internally by the instrument after locking and shimming to the deuterated solvent (^{31}P, ^{35}Cl and ^{127}I). NMR multiplicities are abbreviated as follows: s = singlet, d = doublet, t = triplet, q = quartet, p = pentet, sept = septet, m = multiplet, br = broad signal. Mass spectrometry was recorded using an Orbitrap LTQ XL (Thermo Scientific) spectrometer.

Reagents and Handling: [**1a**]Cl was synthesized according to literature procedure.[1] All compounds were purchased from commercial sources (Sigma Aldrich, Alfa Aesar, Tokyo Chemical Industry) and used as received, if not stated differently. To prepare the iodine stock solution in diethyl ether, an arbitrary amount of iodine was filled into a pre-weighed argon-filled Schlenk tube, dried at 21 °C for 30 min *in vacuo* and then stored under argon. After re-weighing the iodine, it was mixed with the corresponding amount of dry diethyl ether.

1.2 Preparation of [1b]Cl

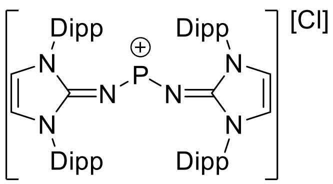

IDippNTMS (1,3-bis(2,6-diisopropylphenyl)-*N*-(trimethylsilyl)-imidazolin-2-imine) (2.29 g, 4.82 mmol, 2.10 eq.) was dissolved in THF (20 mL). At -78 °C, PCl_3 (0.20 mL, 2.3 mmol, 1.0 eq.) was added while stirring. The reaction mixture was warmed to 21 °C and then stirred for 20 h at that temperature. The mixture was dried *in vacuo* and washed with diethyl ether (2x15 mL). After drying again *in vacuo*, the product was obtained as a yellow solid.

Note: The NMR signals were assigned using 2D NMR experiments (*vide infra*).

Yield: 72% (1.65 mmol, 1.39 g).

^{1}H NMR (CD_3CN, 400 MHz, 298 K): δ (ppm) = 7.45 (t, $^3J_{HH}$ = 7.8 Hz, 4 H, Dipp; *para*), 7.19 (d, $^3J_{HH}$ = 7.8 Hz, 8 H, Dipp; *meta*), 7.12 (s, 4 H, N-CH=CH-N), 2.35 (sept, $^3J_{HH}$ = 6.9 Hz, 8 H, CH *i*Pr), 1.09 (d, $^3J_{HH}$ = 6.9 Hz, 24 H, CH_3 *i*Pr), 0.82 (d, $^3J_{HH}$ = 6.9 Hz, 24 H, CH_3 *i*Pr).

^{13}C NMR (CD_3CN, 101 MHz, 298 K): δ (ppm) = 148.2 (d, $^2J_{CP}$ = 20 Hz, N_2C=N), 147.2 (C_q Dipp; *ortho*), 132.0 (CH Dipp; *para*), 130.8 (C_q Dipp; *ipso*), 125.3 (CH Dipp; *meta*), 119.8 (CH N-CH=CH-N), 29.7 (CH *i*Pr), 24.3 (CH_3 *i*Pr), 23.5 (CH_3 *i*Pr), 23.4 (CH_3 *i*Pr).

^{31}P NMR (CD_3CN, 162 MHz, 298 K): δ (ppm) = 308.4.

^{35}Cl NMR (CD_3CN, 39 MHz, 300 K): δ (ppm) = 44.7.

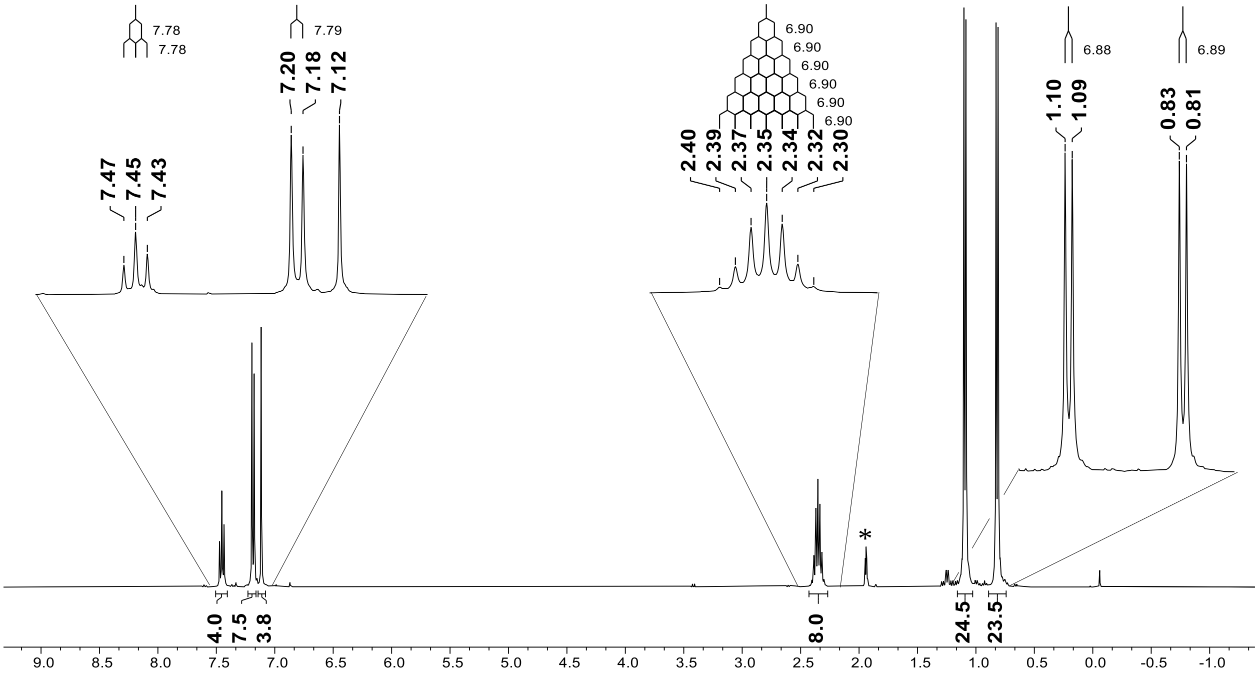

Figure S1: ^{1}H NMR (400 MHz, 298 K) spectrum of [**1b**]Cl in CD_3CN. The asterisk marks the solvent signal.

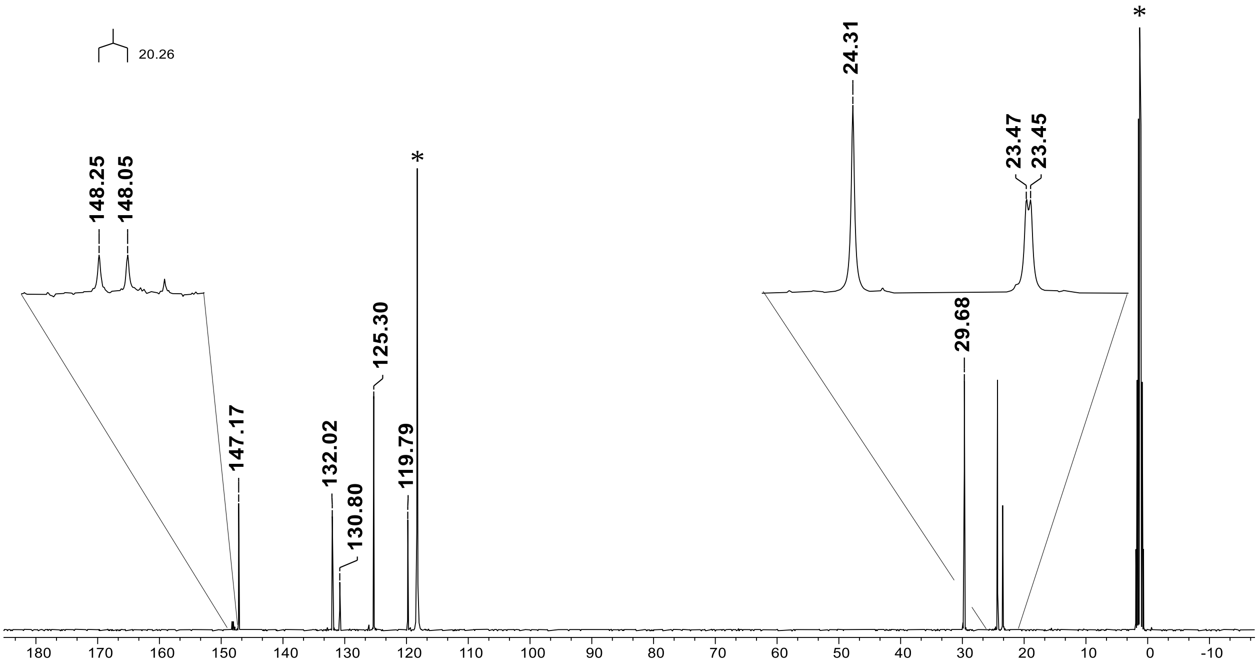

Figure S2: $^{13}C\{^{1}H\}$ NMR (101 MHz, 298 K) spectrum of [**1b**]Cl in CD_3CN. The asterisks mark the solvent signal.

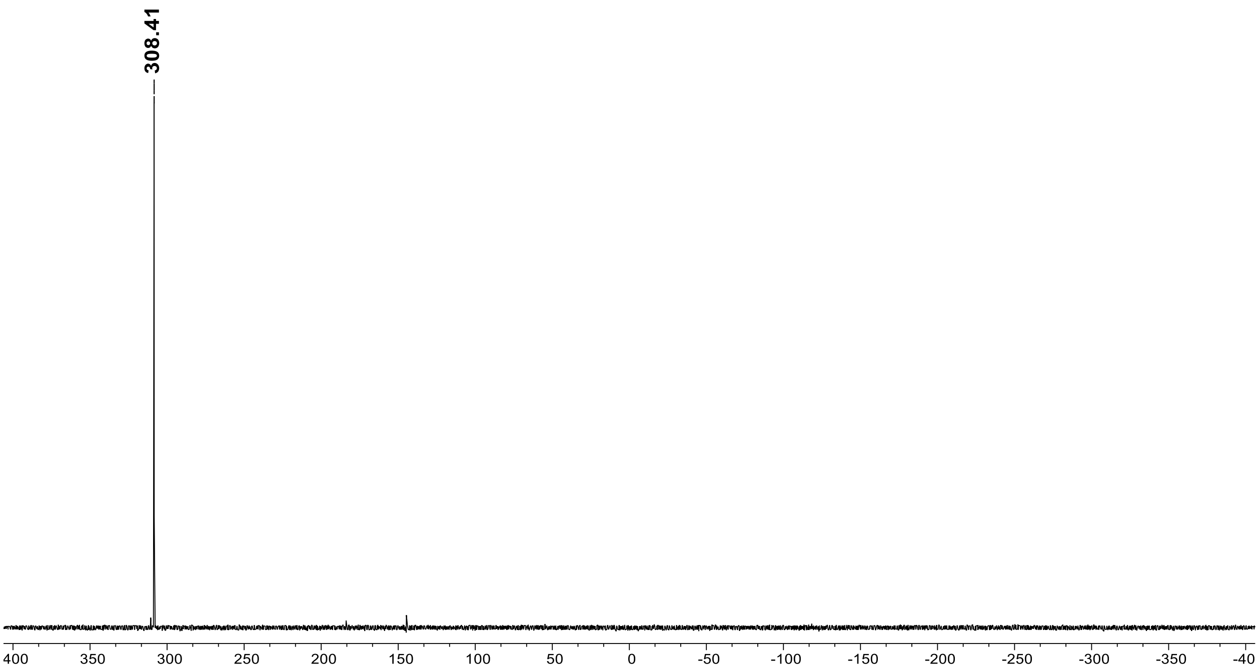

Figure S 3: ^{31}P NMR (162 MHz, 298 K) spectrum of [**1b**]Cl in CD_3CN.

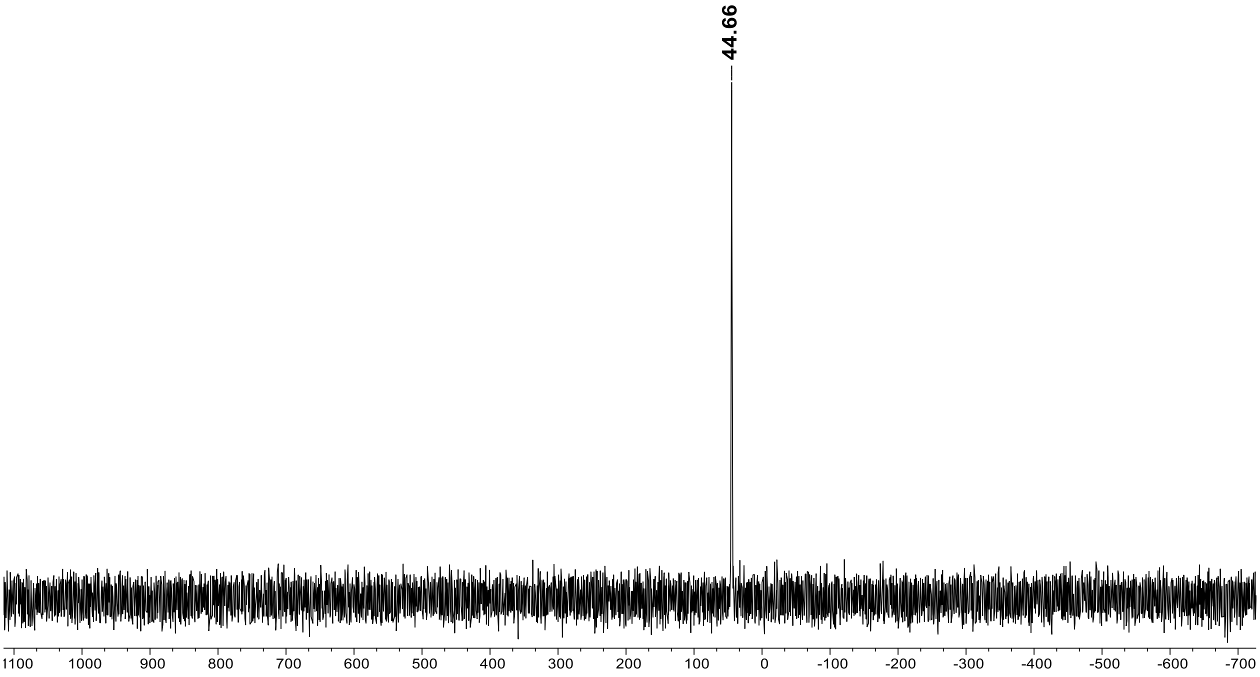

Figure S 4: ^{35}Cl NMR (39 MHz, 300 K) spectrum of [**1b**]Cl in CD_3CN.

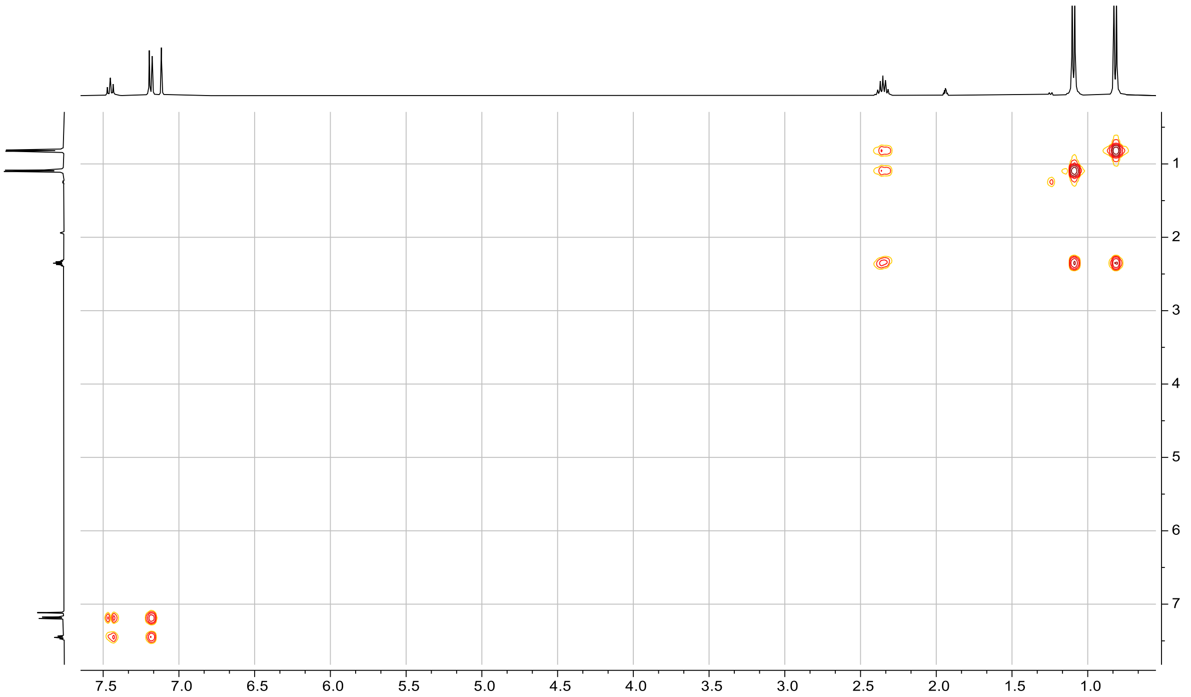

Figure S5: $^{1}H/^{1}H$ COSY NMR spectrum of [**1b**]Cl in CD_3CN.

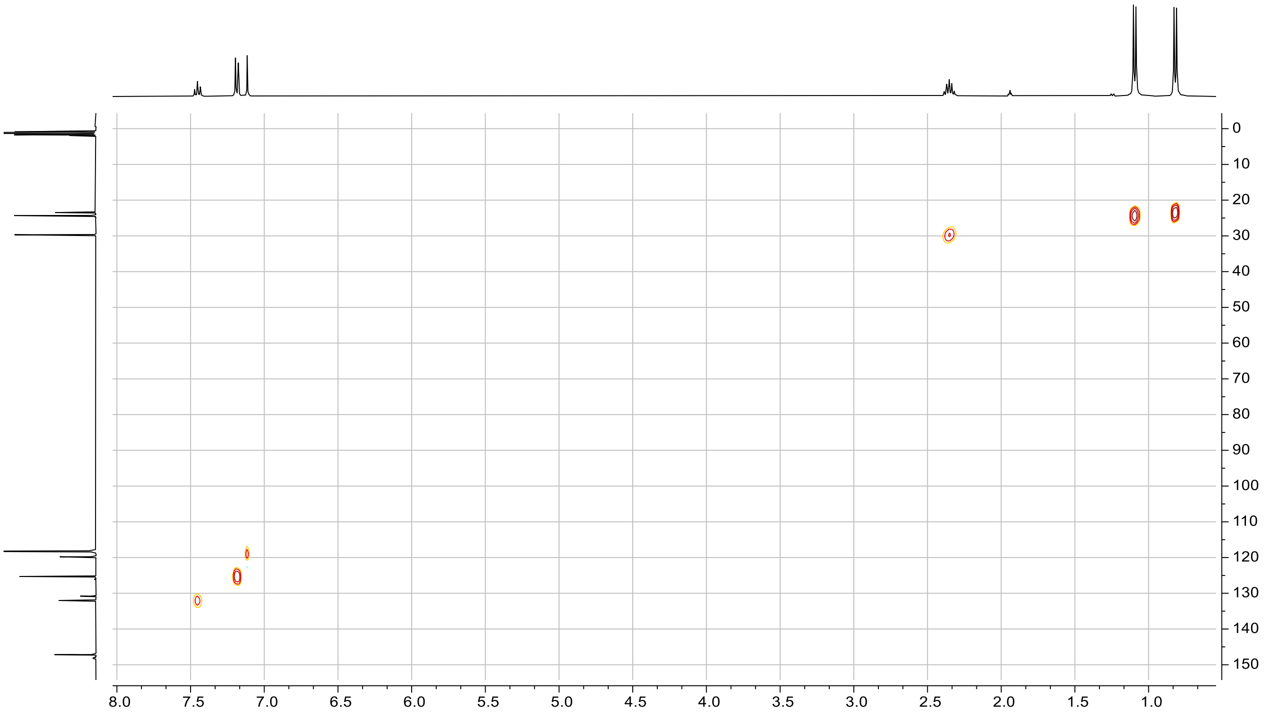

Figure S6: $^{1}H/^{13}C\{^{1}H\}$ HSQC NMR spectrum of [**1b**]Cl in CD_3CN.

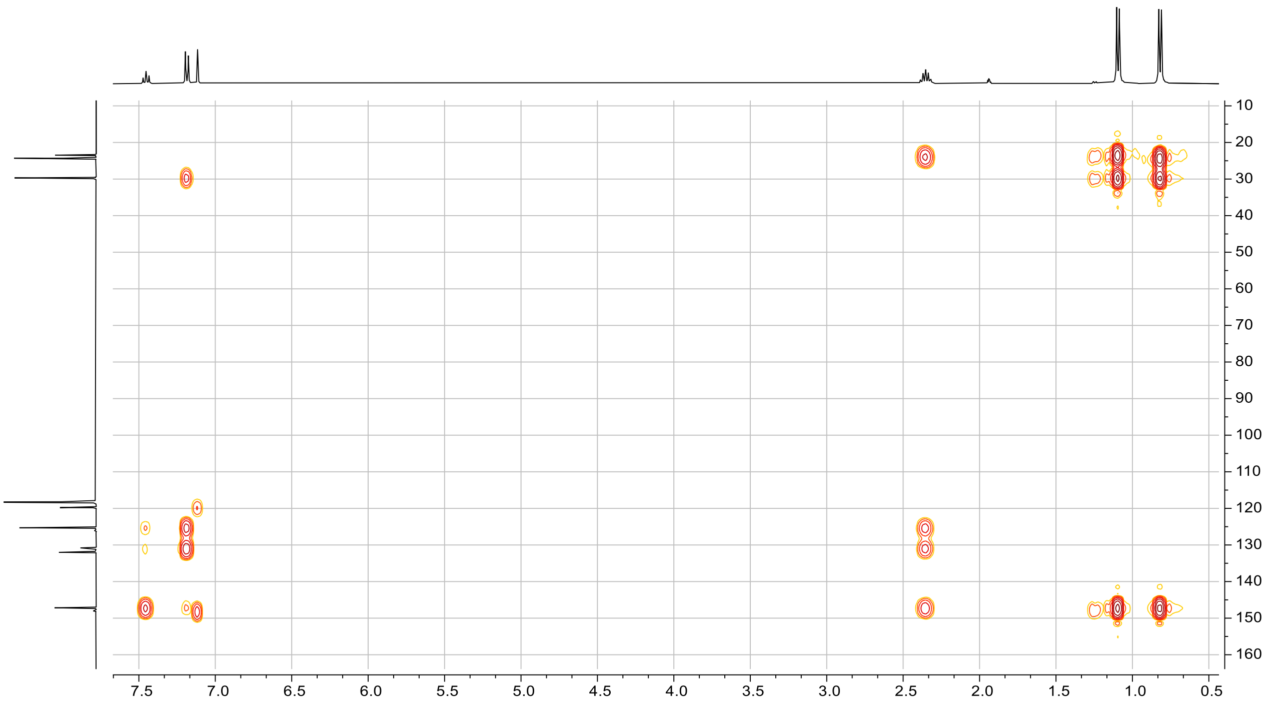

Figure S7: $^{1}H/^{13}C\{^{1}H\}$ HMBC NMR spectrum of [**1b**]Cl in CD_3CN.

1.3 Preparation of 2a

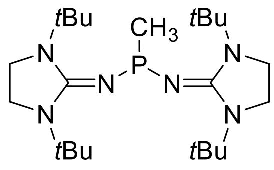

Bis((1,3-di-*tert*-butylimidazolidin-2-ylidene)amino)phosphenium chloride ([**1a**]Cl) (4.36 mmol, 2.00 g, 1.00 eq.) was suspended in tetrahydrofuran (50 mL). The mixture was cooled to -78°C and a solution of methylmagnesium chloride in tetrahydrofuran (3 M, 1.45 mL, 1.00 eq.) was quickly added. The reaction mixture was immediately allowed to warm up to 21 °C and was stirred for 16 h. All volatiles were removed *in vacuo*. The crude product was extracted with *n*-hexane (50 mL). After the solvent was removed *in vacuo*, the product was obtained as a white crystalline powder.

Note: The NMR signals were assigned using 2D-NMR experiments (*vide infra*).

Yield: 1.67 g (3.81 mmol, 87 %).

^{1}H NMR (C_6D_6, 400 MHz, 300 K): δ (ppm) = 2.82 (s, 8 H, N-CH_2-CH_2-N), 1.78 (d, $^{2}J_{PH}$ = 8.8 Hz, 3 H, P-CH_3), 1.49 (s, 36 H, *t*Bu).

^{1}H{^{31}P} NMR (C_6D_6, 400 MHz, 300 K): δ (ppm) = 2.82 (s, 8 H, N-CH_2-CH_2-N), 1.78 (s, P-CH_3), 1.49 (s, 36 H, *t*Bu).

^{13}C NMR (C_6D_6, 101 MHz, 300 K): δ (ppm) = 149.3 (d, $^{2}J_{CP}$ = 18 Hz, N-C-N), 53.7 (m, $\underline{C}$-Me_3), 42.6 (tt, $^{1}J_{CH}$ = 141 Hz, $^{2}J_{CH}$ = 4 Hz, N-CH_2-CH_2-N), 28.9 (qm, $^{1}J_{CH}$ = 126 Hz, C-$\underline{C}H_3$), 24.1 (qd, $^{1}J_{CH}$ = 126 Hz, $^{1}J_{CP}$ = 10 Hz, P-CH_3).

^{13}C{^{1}H} NMR (C_6D_6, 101 MHz, 300 K): δ (ppm) = 149.3 (d, $^{2}J_{CP}$ = 18 Hz, N-C-N), 53.7 ($\underline{C}$-Me_3), 42.6 (N-CH_2-CH_2-N), 28.9 (C-$\underline{C}H_3$), 28.8 (C-$\underline{C}H_3$), 24.1 (d, $^{1}J_{CP}$ = 10 Hz, P-CH_3).

^{31}P NMR (C_6D_6, 162 MHz, 300 K): δ (ppm) = 69.3-69.2 (m).

^{31}P{^{1}H} NMR (C_6D_6, 162 MHz, 300 K): δ (ppm) = 69.3.

Elemental analysis: Calculated for $C_{23}H_{47}N_6P$ (**2a**): C 62.98%, H 10.80%, N 19.16%; found: C 62.73%, H 10.58%, N 18.96%.

HR-MS(ESI): Calculated for $[C_{23}H_{48}N_6P]^+$ ([**2a**+H]$^+$): m/z = 455.35998, found: m/z = 455.35988.

Melting point: 102°C (clear liquid).

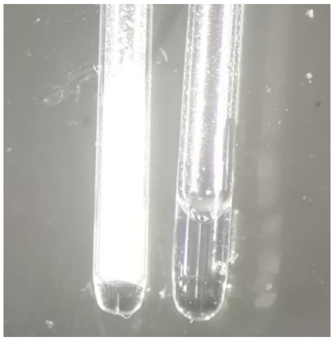

Figure S 8: Crystalline (left) and molten-then-resolidified (right) **2a** in glass capillaries.

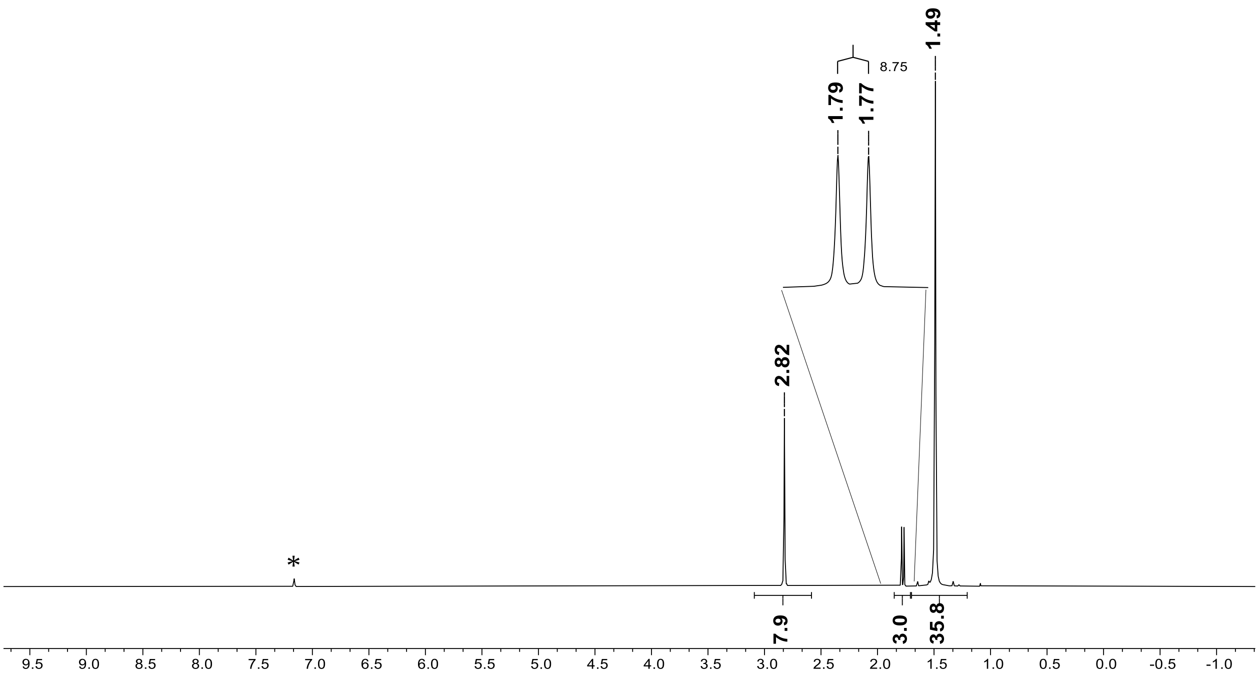

Figure S 9: ^{1}H NMR (400 MHz, 300 K) spectrum of **2a** in C_6D_6. The asterisk marks the solvent signal.

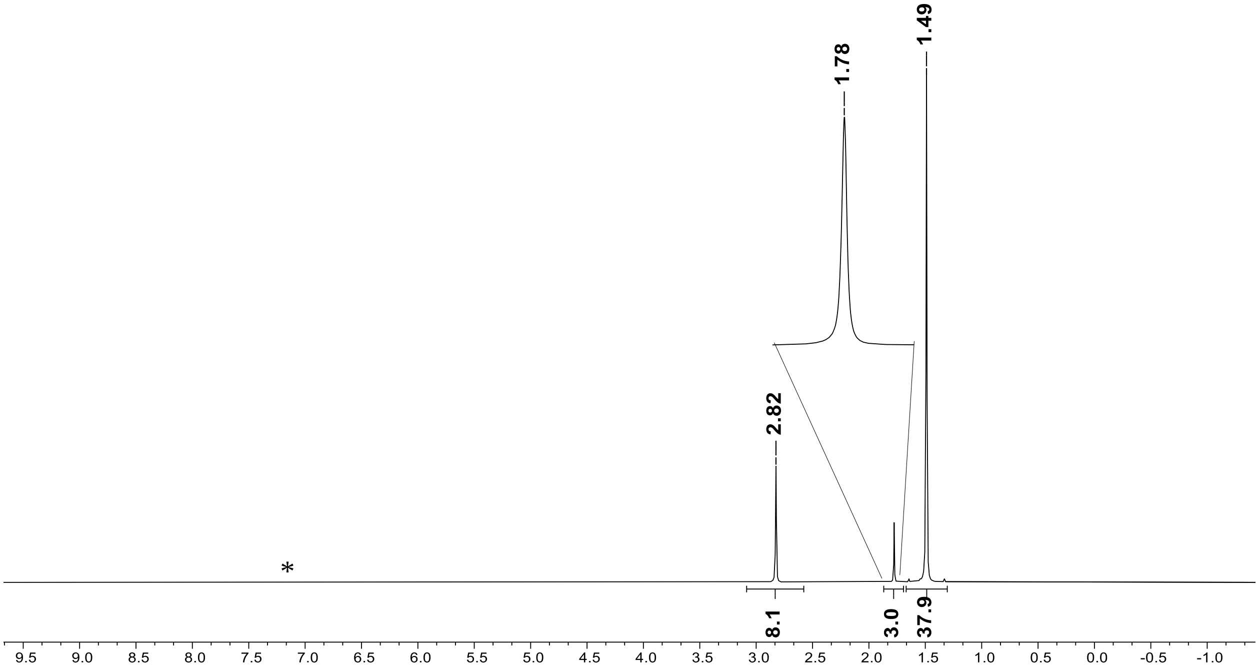

Figure S 10: $^{1}H\{^{31}P\}$ (400 MHz, 300 K) NMR spectrum of **2a** in C_6D_6. The asterisk marks the solvent signal (too weak to be seen at the given amplification level).

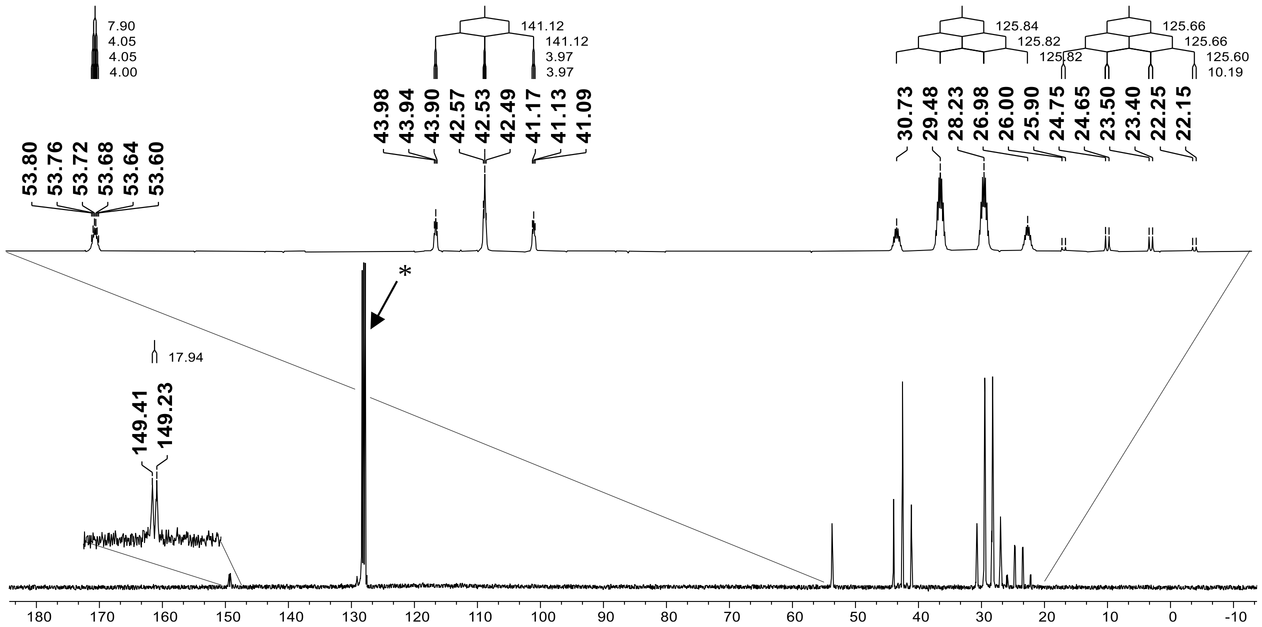

Figure S 11: ^{13}C NMR (101 MHz, 300 K) spectrum of **2a** in C_6D_6. The asterisk marks the solvent signal.

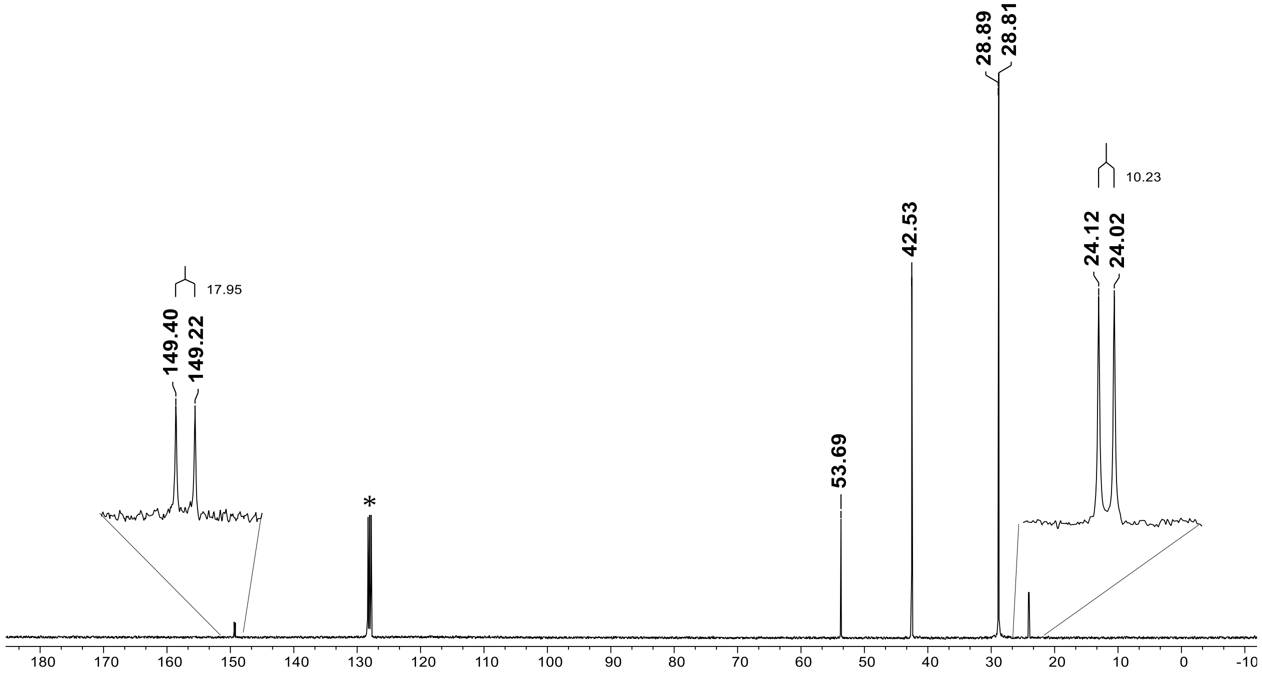

Figure S 12: $^{13}C\{^{1}H\}$ NMR (101 MHz, 300 K) spectrum of **2a** in C_6D_6. The asterisk marks the solvent signal.

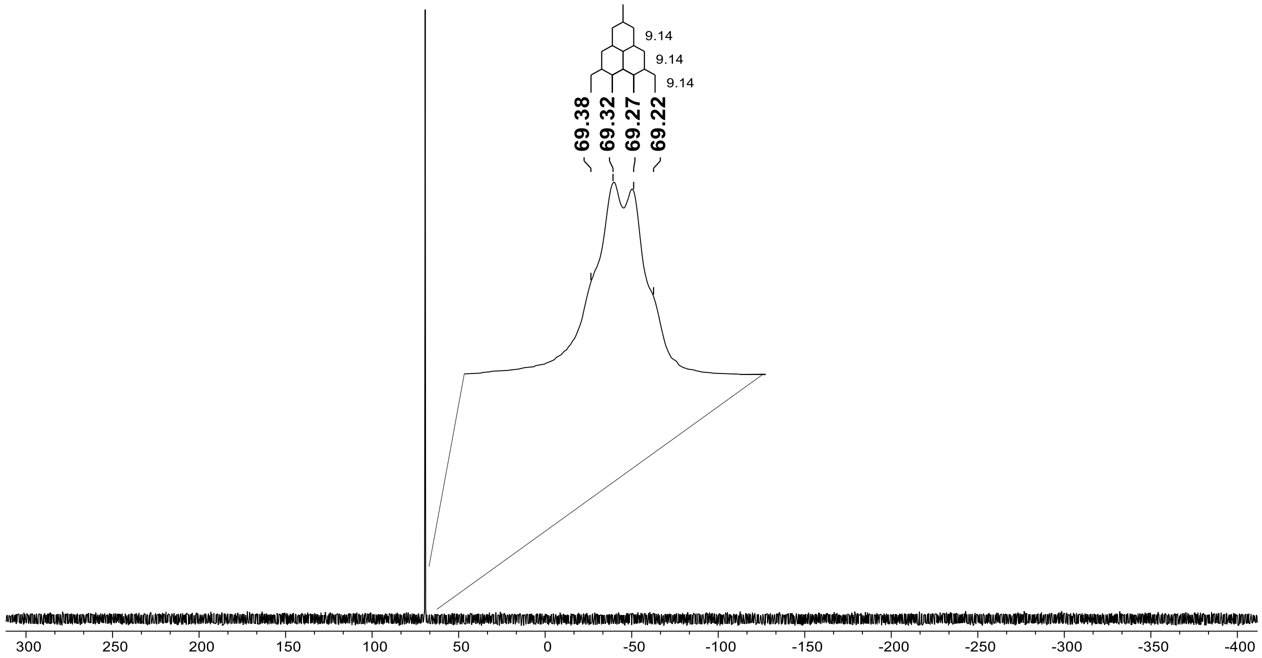

Figure S 13: ^{31}P (162 MHz, 300 K) NMR spectrum of **2a** in C_6D_6.

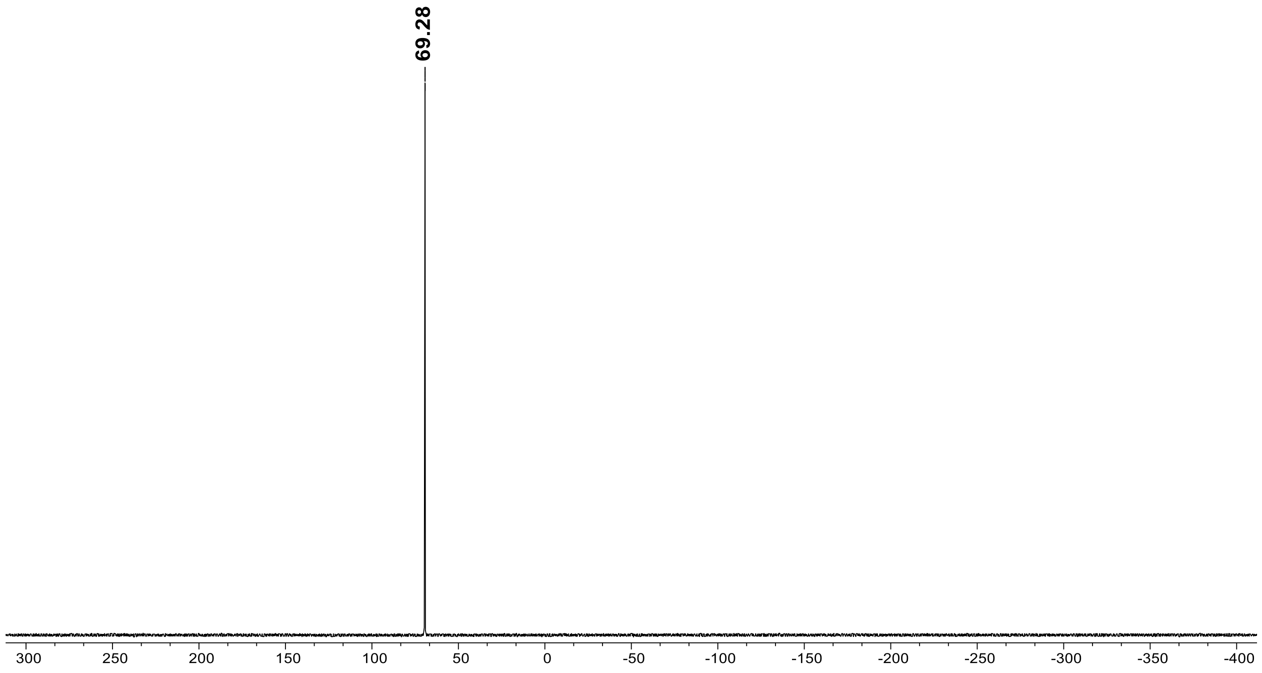

Figure S 14: $^{31}P\{^1H\}$ NMR (162 MHz, 300 K) spectrum of **2a** in C_6D_6.

1.4 Preparation of 2b

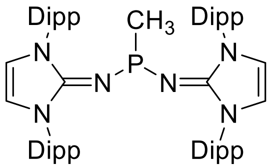

To a suspension of [**1b**]Cl (1.15 mmol, 1.00 g, 1.00 eq.) in tetrahydrofuran (30 mL) methylmagnesium chloride in tetrahydrofuran (3 M, 0.38 mL, 1.00 eq.) was added at 21 °C. The solution was stirred for 12 h. Subsequently, all volatiles were removed *in vacuo*. The residue was suspended in a mixture of toluene and *n*-hexane (1:1, 50 mL) and the suspension was filtrated. All volatiles were removed *in vacuo* and the product was obtained as a pale yellow solid.

Note: The NMR signals were assigned using 2D NMR experiments (*vide infra*).

Yield: 874 mg (1.02 mmol, 89%).

^{1}H NMR (C_6D_6, 400 MHz, 300 K): δ (ppm) = 7.26 (t, $^{3}J_{HH}$ = 7.7 Hz, 4 H, CH Dipp; *para*), 7.12 - 7.05 (m, 8 H, CH Dipp), 5.82 (s, 4 H, N-CH=CH-N), 3.21 (sept, $^{3}J_{HH}$ = 6.9 Hz, 4 H, CH *i*Pr), 3.06 (sept, $^{3}J_{HH}$ = 6.9 Hz, 4 H, CH *i*Pr), 1.27 - 1.05 (m, 48 H, CH_3 *i*Pr), 0.24 (d, $^{2}J_{HP}$ = 7.1 Hz, 3 H, CH_3 P-Me).

^{13}C NMR (C_6D_6, 101 MHz, 300 K): δ (ppm) = 148.7 - 147.3 (C_q Dipp; *ortho*), 141.1 (d, $^{2}J_{CP}$ = 21 Hz, C_q C=N-P), 135.5 (C_q Dipp; *ipso*), 128.8 (CH Dipp; *para*), 123.8-123.5 (C_q Dipp; *meta*), 114.5 (CH N-CH=CH-N), 29.0 - 28.8 (CH *i*Pr), 27.0 (d, $^{1}J_{CP}$ = 23 Hz, CH_3 P-Me), 24.8-23.4 (CH_3 *i*Pr).

^{31}P NMR (C_6D_6, 162 MHz, 300 K): δ (ppm) = 62.0 (q, J_{PH} = 7 Hz).

Elemental analysis: Calculated for $C_{55}H_{75}N_6P$ (**2b**): C 77.61%, H 8.88%, N 9.87%; found: C 77.63%, H 8.96%, N 9.52%.

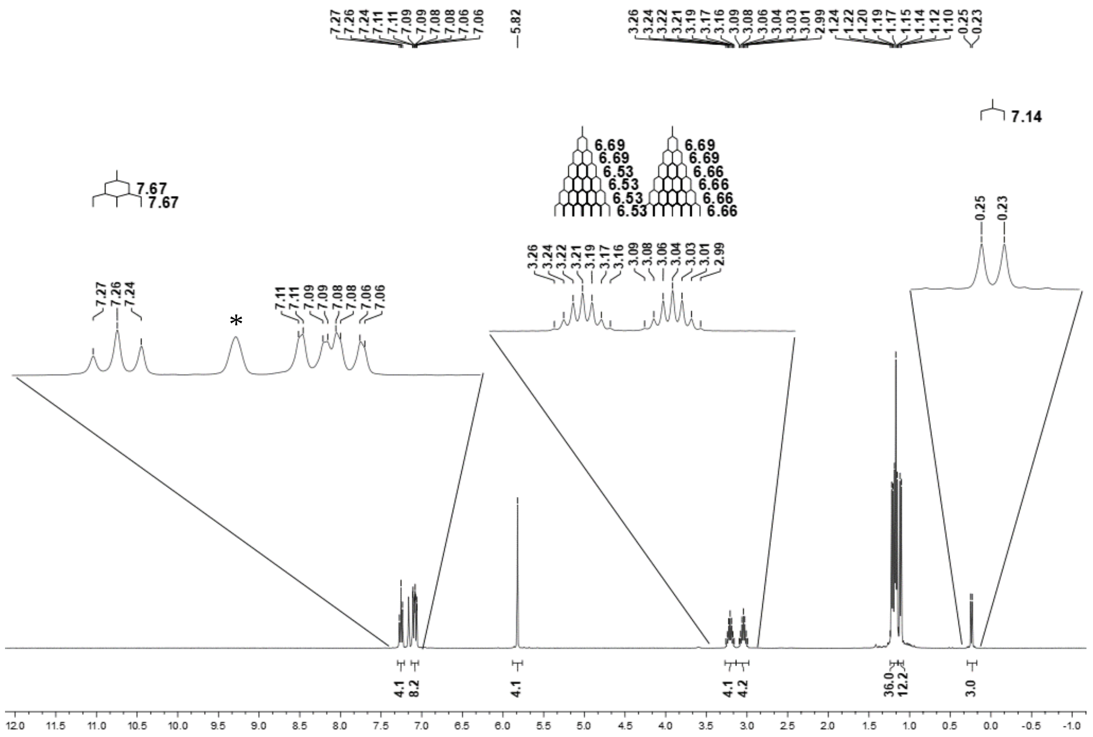

Figure S15: ^{1}H NMR (400 MHz, 300 K) spectrum of **2b** in C_6D_6. The asterisk marks the solvent signal.

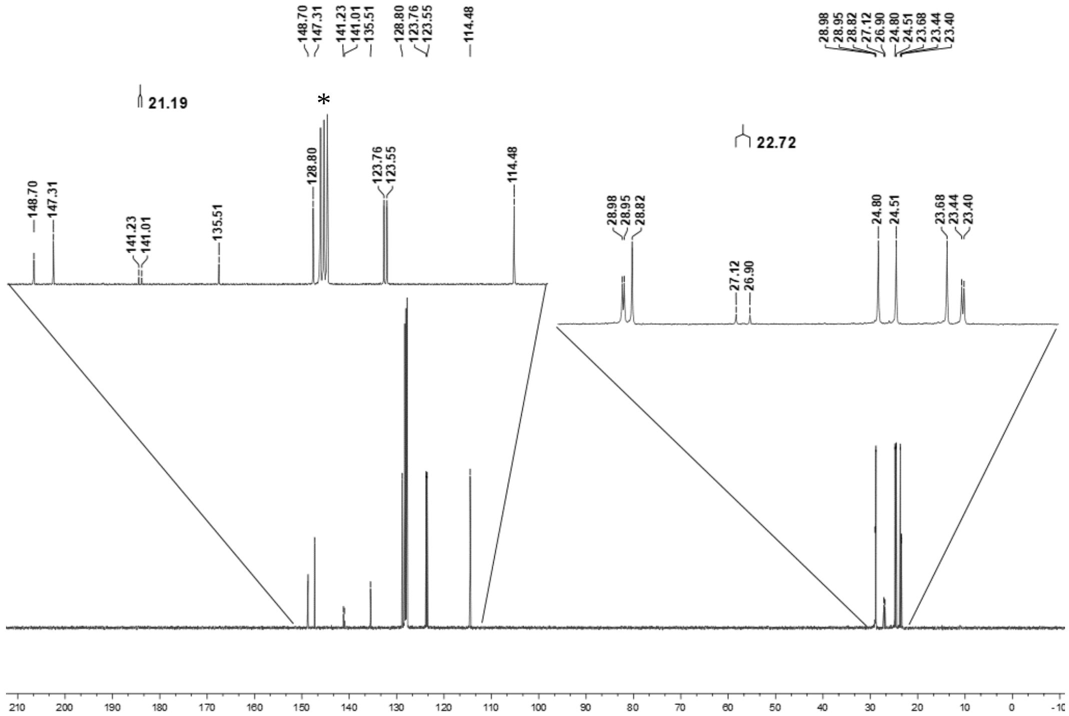

Figure S16: $^{13}C\{^{1}H\}$ NMR (101 MHz, 300 K) spectrum of **2b** in C_6D_6. The asterisk marks the solvent signal.

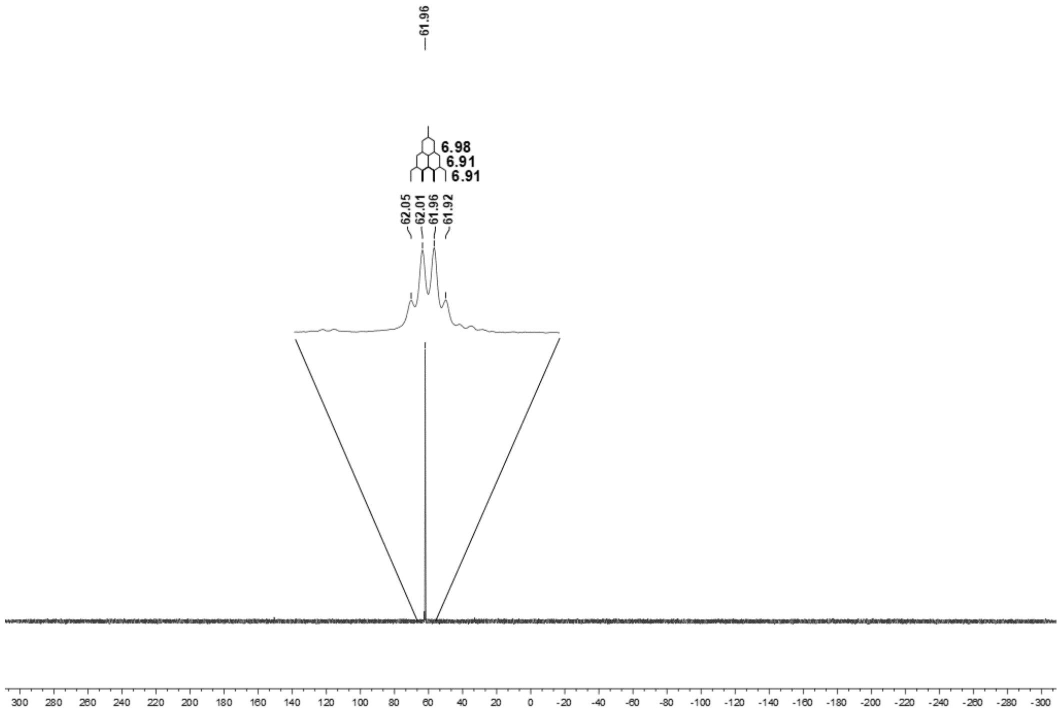

Figure S17: ^{31}P NMR (162 MHz, 300 K) spectrum of **2b** in C_6D_6.

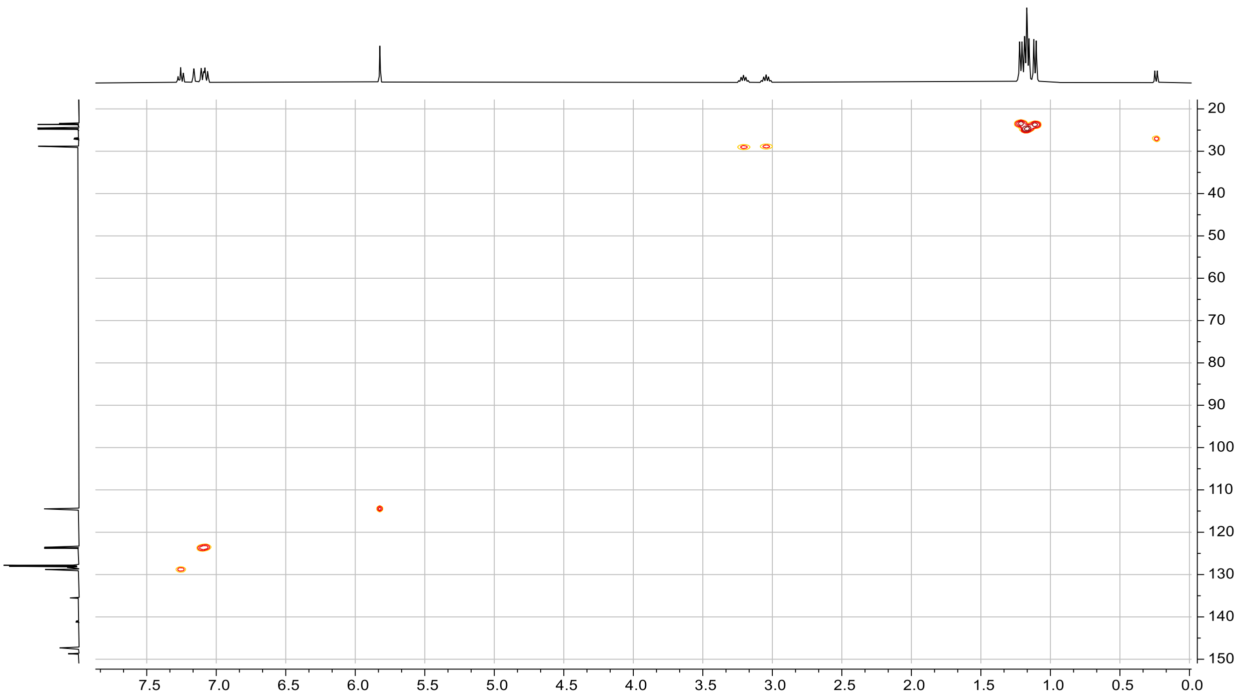

Figure S18: $^{1}H/^{13}C\{^{1}H\}$ HSQC NMR spectrum of **2b** in C_6D_6.

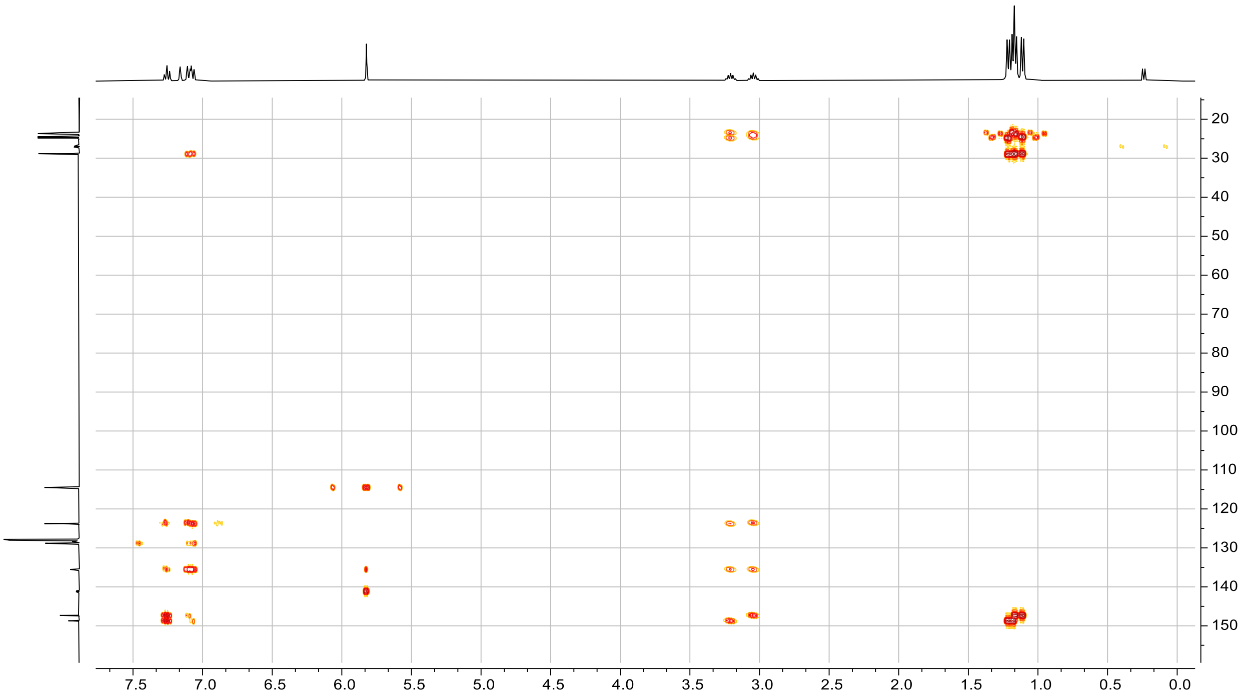

Figure S19: $^{1}H/^{13}C\{^{1}H\}$ HMBC NMR spectrum of **2b** in C_6D_6.

1.5 Preparation of [3a]I

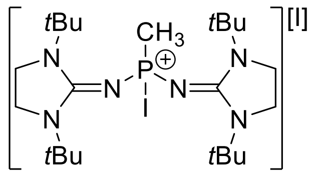

2a (3.96 mmol, 1.74 g, 1.00 eq.) was dissolved in diethyl ether (50 mL). The clear solution was cooled to -78°C *via* a dry ice/acetone cooling bath. While vigorously stirring the solution, a freshly prepared stock solution of iodine in diethyl ether (0.442 M, 3.96 mmol, 1.00 eq.) was added dropwise directly into the vortex of the stirred mixture over a time span of 30 minutes. The suspension was stirred for 16 h while keeping it in the cold bath, concurrently allowing the reaction mixture to warm up to 21 °C over a time span of several hours. The white precipitate was isolated via filtration and washed subsequently with tetrahydrofuran (2x10 mL) and diethyl ether (2x10 mL). The residue was dried *in vacuo* at 21 °C for 2 h. The product was obtained as a white, slightly sticky powder.

Note: The NMR signals were assigned using 2D-NMR experiments (*vide infra*). Too weak stirring will result in the formation of an unidentified byproduct. A slight excess of iodine will result in the formation of red [**3a**]I_3, which can be partially washed out with tetrahydrofuran in the workup (*vide infra*).

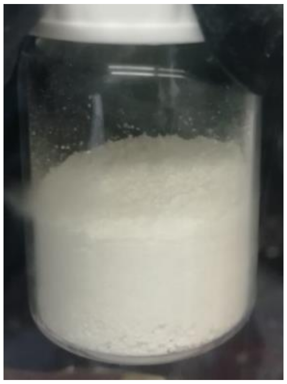

Figure S 20: Batch of [**3a**]I in a scintillation vial.

Yield: 2.56 g (3.70 mmol, 93 %).

^{1}H NMR (CD_2Cl_2, 400 MHz, 300 K): δ (ppm) = 3.56 (m, 8 H, N-CH_2-CH_2-N), 2.61 (d, $^2J_{PH}$ = 14.5 Hz, 3 H, P-CH_3), 1.45 (s, 36 H, *t*Bu).

^{13}C{^{1}H} NMR (CD_2Cl_2, 101 MHz, 300 K): δ (ppm) = 155.7 (d, $^2J_{CP}$ = 14 Hz, N-C-N), 56.8 ($\underline{C}$-Me_3), 43.2 (N-CH_2-CH_2-N), 34.3 (d, $^1J_{CP}$ = 119 Hz, P-CH_3), 29.1 (C-$\underline{C}H_3$).

^{31}P NMR (CD_2Cl_2, 202 MHz, 300 K): δ (ppm) = -71.6 (q, $^2J_{PH}$ = 14 Hz).

^{31}P{^{1}H} NMR (CD_2Cl_2, 202 MHz, 300 K): δ (ppm) = -71.6.

^{127}I NMR (CD_2Cl_2, 80 MHz, 300 K): δ (ppm) = 62 (br, $w_{1/2}$ = 8500 Hz).

Elemental analysis: Calculated for $C_{23}H_{47}I_2N_6P$ ([**3a**]I): C 39.89%, H 6.84%, N 12.14%; found: C 39.82%, H 6.84%, N 12.34%.

HR-MS(ESI): Calculated for $[C_{23}H_{47}N_6PI]^+$ ([**3a**]$^+$): m/z = 565.26390, found: m/z = 565.26148.

Melting point: 143°C (decomposition).

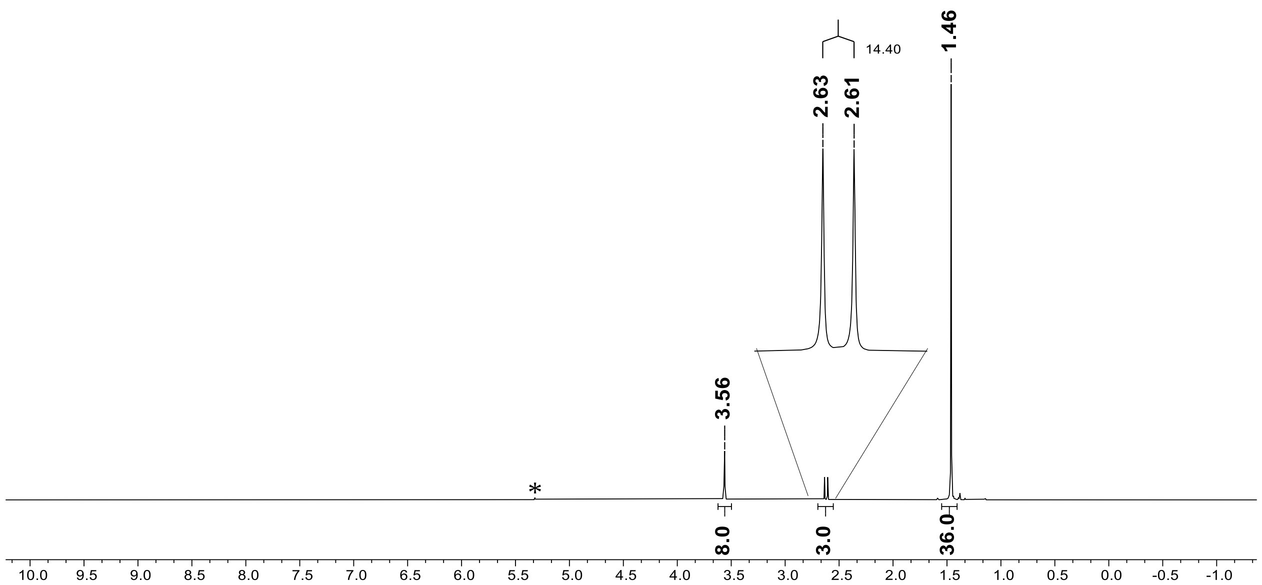

Figure S21: ^{1}H NMR (400 MHz, 300 K) spectrum of [**3a**]I in CD_2Cl_2. The asterisk marks the solvent signal (too weak to be seen at the given amplification level).

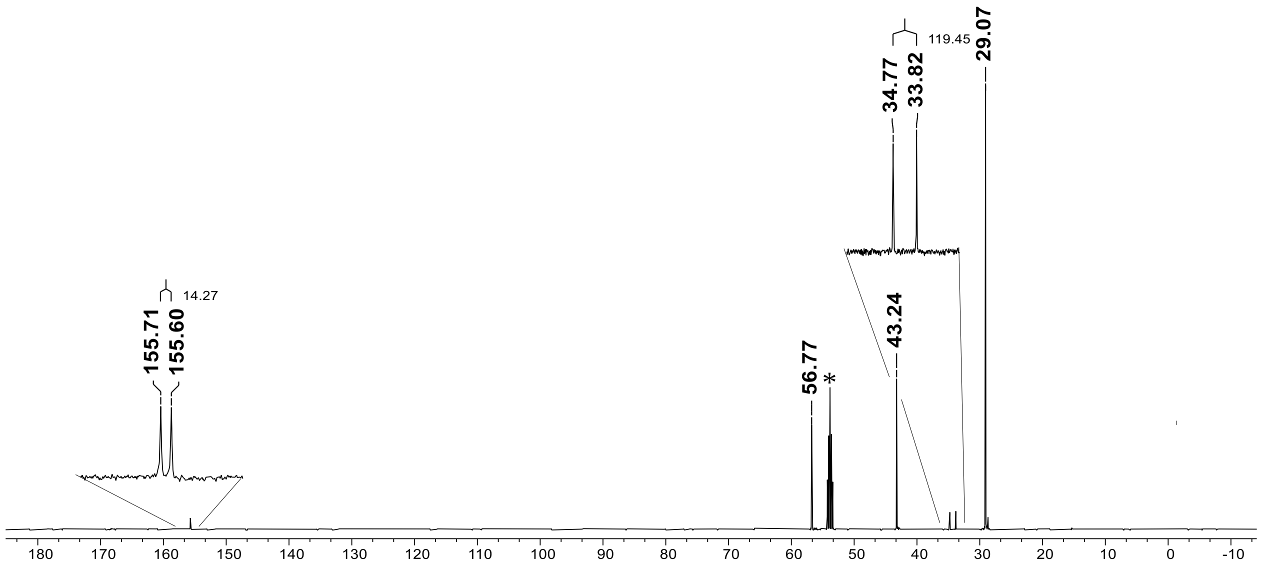

Figure S22: $^{13}C\{^{1}H\}$ NMR (101 MHz, 300 K) spectrum of [**3a**]I in CD_2Cl_2. The asterisk marks the solvent signal.

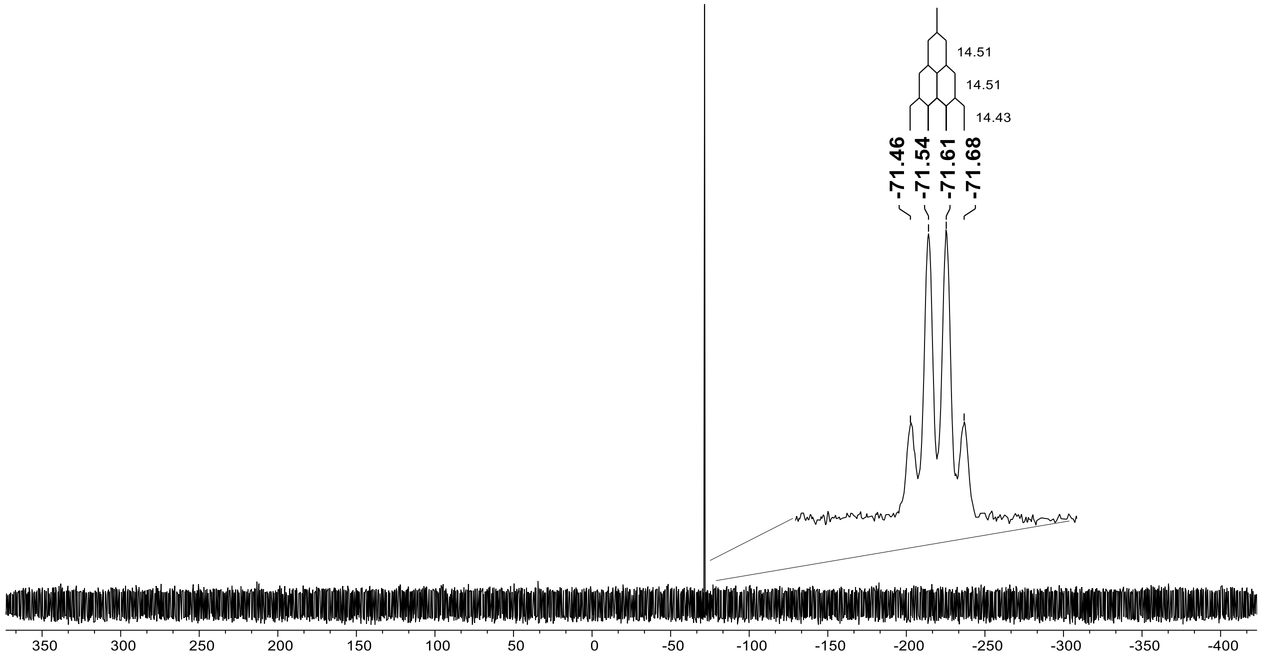

Figure S23: ^{31}P NMR (202 MHz, 300 K) spectrum of [**3a**]I in CD_2Cl_2.

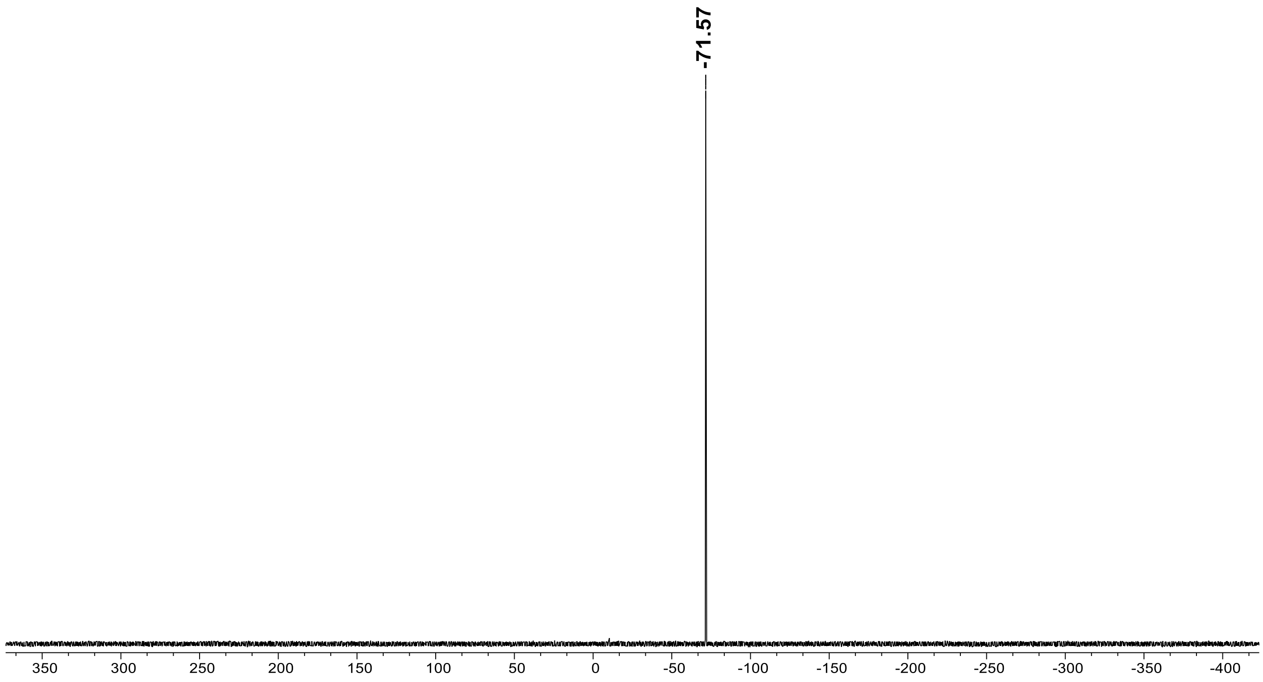

Figure S24: ^{31}P NMR (202 MHz, 300 K) spectrum of [**3a**]I in CD_2Cl_2.

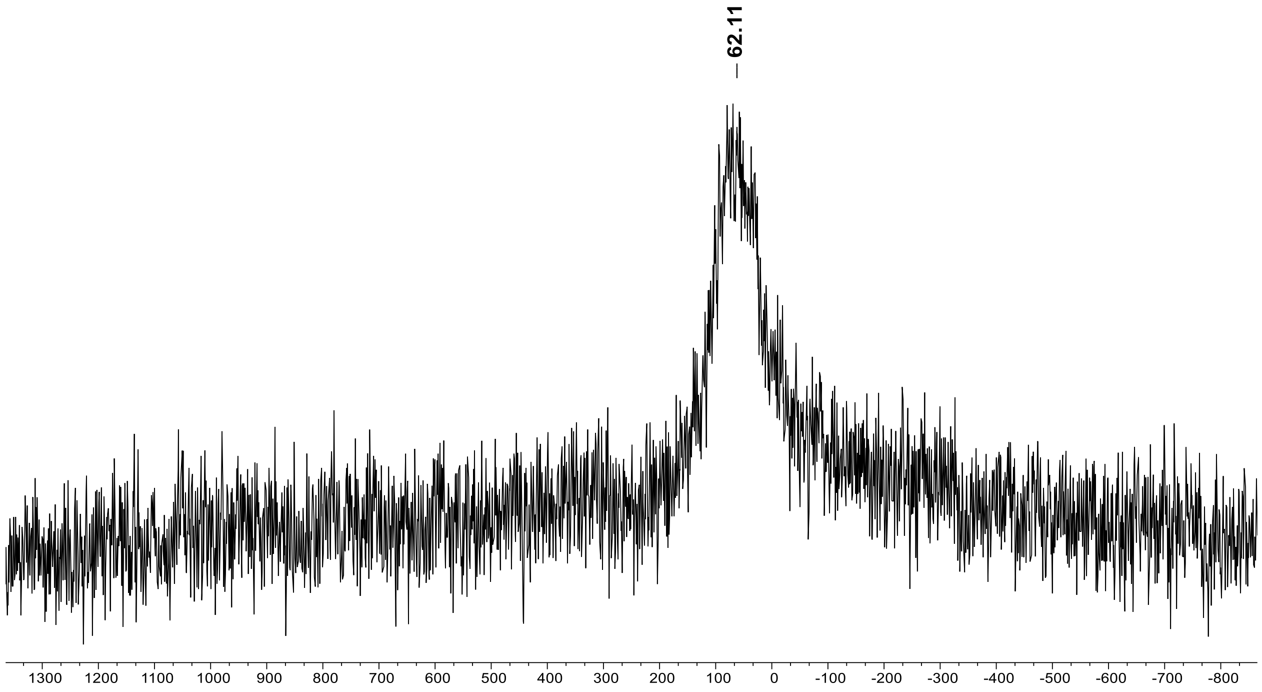

Figure S 25: ^{127}I NMR (80 MHz, 300 K) spectrum of [**3a**]I in CD_2Cl_2.

1.6 Characterization data of [3a]I3

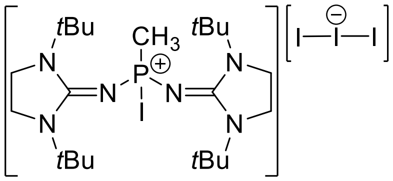

Traces of [**3a**]I_3 form in the synthesis of [**3a**]I (*vide supra*) when a slight excess of iodine is used. This results in a yellow to red crude product. [**3a**]I_3 can be partially washed out by washing the crude product with tetrahydrofuran. Red crystals of [**3a**]I_3 crystallized out of the yellow wash solution and were isolated by pipetting off the mother liquor and dried *in vacuo* for 2 h at 21°C.

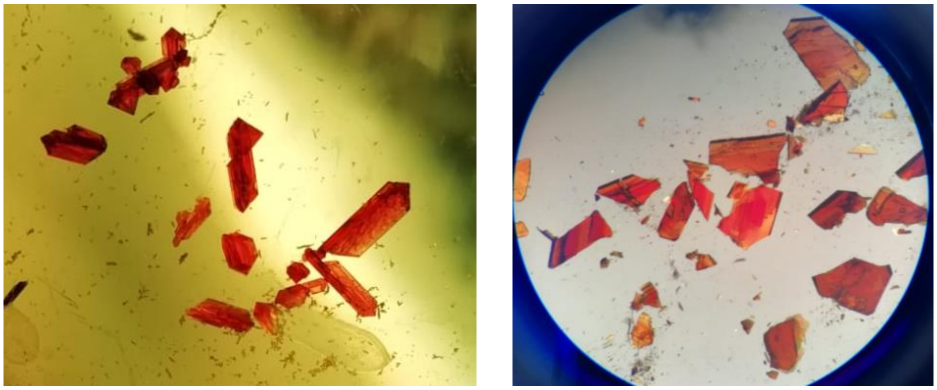

Figure S 26: Crystals of [**3a**]I_3 in the tetrahydrofuran wash solution (left) and in oil under a microscope (right).

Note: The ^{1}H, ^{13}C{^{1}H} and ^{31}P NMR shifts of [**3a**]I_3 are almost identical to the ones of [**3a**]I.

^{1}H NMR (CD_2Cl_2, 400 MHz, 300 K): δ (ppm) = 3.56 (d, $^{5}J_{HP}$ = 1.9 Hz, 8 H, N-CH_2-CH_2-N), 2.64 (d, $^{2}J_{PH}$ = 14.5 Hz, 3 H, P-CH_3), 1.48 (s, 36 H, *t*Bu).

^{13}C{^{1}H} NMR (CD_2Cl_2, 101 MHz, 300 K): δ (ppm) = 155.8 (d, $^{2}J_{CP}$ = 14 Hz, N-C-N), 57.0 (<u>C</u>-Me_3), 43.4 (N-CH_2-CH_2-N), 34.5 (d, $^{1}J_{CP}$ = 120 Hz, P-CH_3), 29.3 (C-<u>C</u>H_3).

^{31}P NMR (CD_2Cl_2, 162 MHz, 300 K): δ (ppm) = -71.7 (q, $^{2}J_{PH}$ = 14 Hz).

^{31}P{^{1}H} NMR (CD_2Cl_2, 162 MHz, 300 K): δ (ppm) = -71.7.

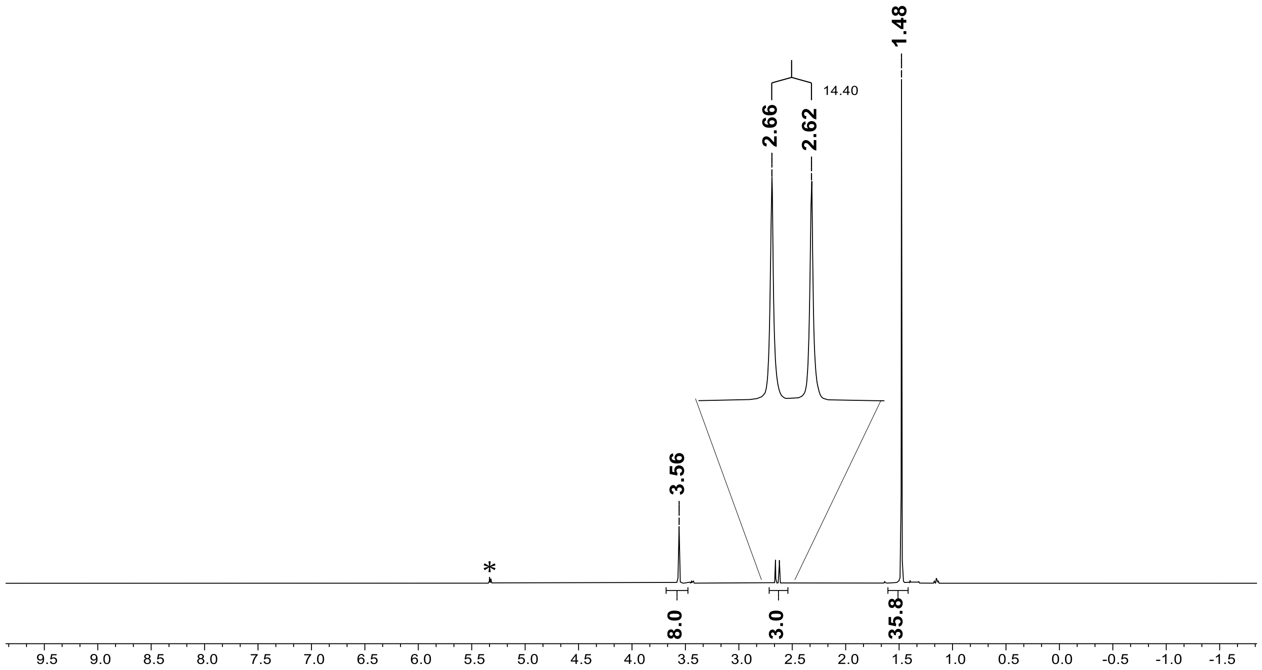

Figure S27: ^{1}H NMR (400 MHz, 300 K) spectrum of [**3a**]I_3 in CD_2Cl_2. The asterisk marks the solvent signal.

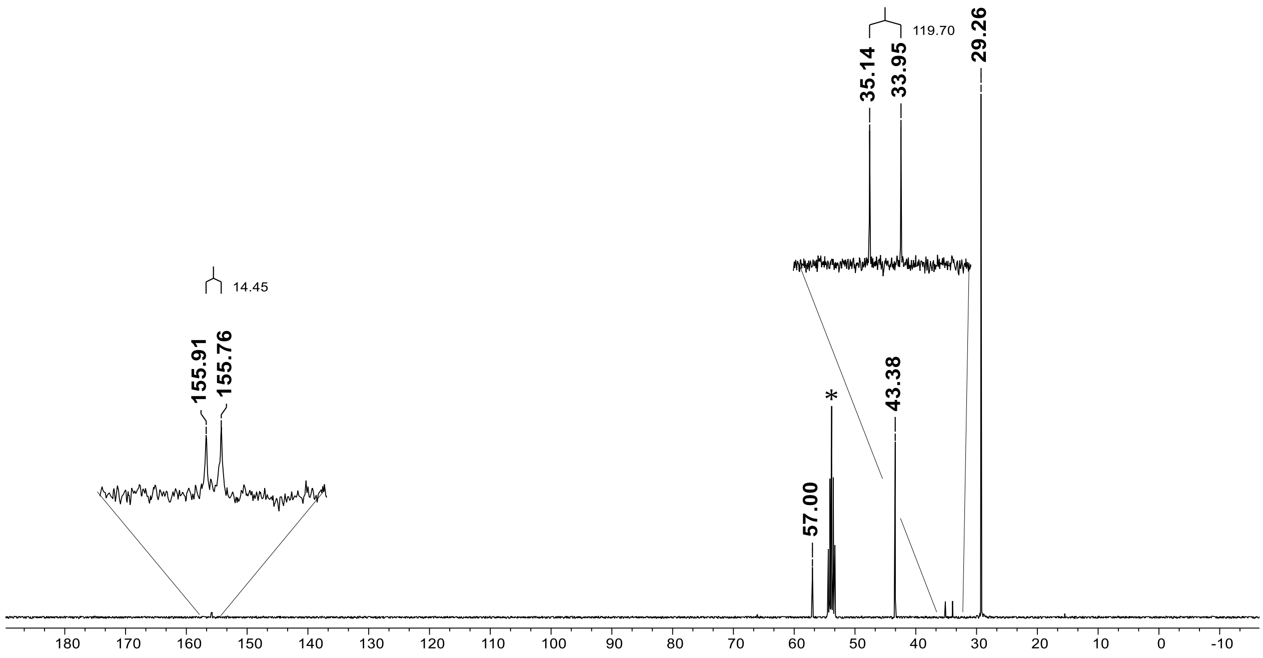

Figure S28: $^{13}C\{^{1}H\}$ NMR (101 MHz, 300 K) spectrum of [**3a**]I_3 in CD_2Cl_2. The asterisk marks the solvent signal.

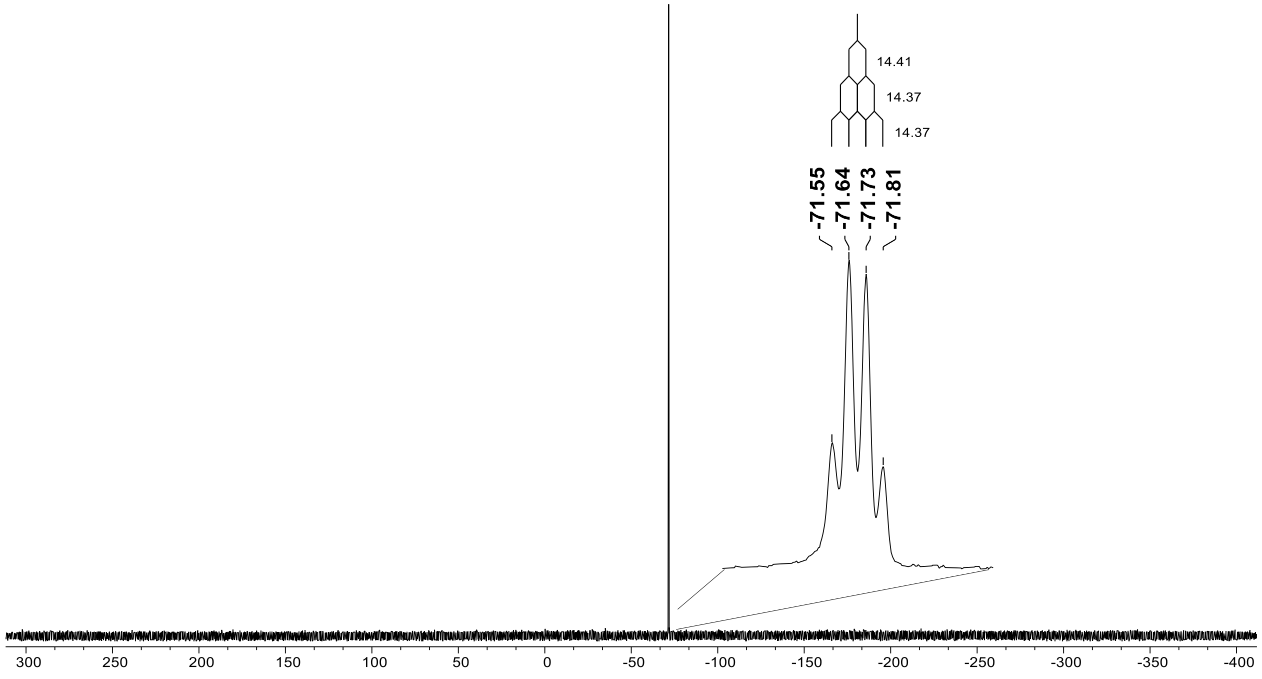

Figure S29: ^{31}P NMR (162 MHz, 300 K) spectrum of [**3a**]I_3 in CD_2Cl_2.

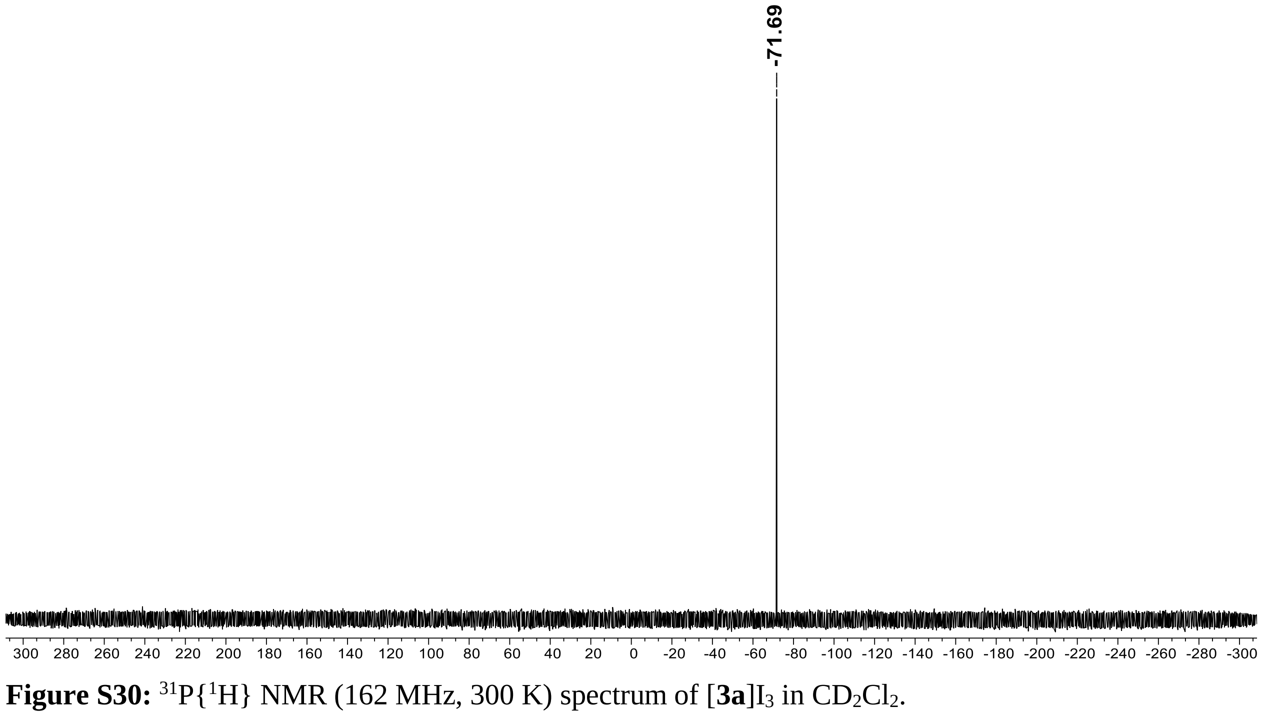

Figure S30: $^{31}P\{^1H\}$ NMR (162 MHz, 300 K) spectrum of [**3a**]I_3 in CD_2Cl_2.

1.7 Preparation of [3b]OTf

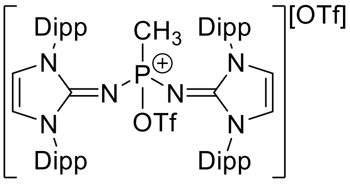

A solution of **2b** (0.17 mmol, 149 mg, 1.0 eq.) in fluorobenzene (3 mL) was added dropwise to a suspension of AgOTf (0.35 mmol, 90 mg, 2.0 eq.) in fluorobenzene (10 mL). After stirring for 12 h at 21 °C, the suspension was filtrated and all volatiles were removed *in vacuo*. The product was obtained as an off-white solid.

Yield: 99% (0.17 mmol, 199 mg).

^{1}H NMR (CD_2Cl_2, 400 MHz, 300 K): δ (ppm) = 7.78 (t, $^3J_{HH}$ = 7.8 Hz, 4 H, CH Dipp; *para*), 7.26-7.21 (m, 8 H, CH Dipp), 6.73 (s, 4 H, N-CH=CH-N), 2.56 (m, 8 H, CH *i*Pr), 1.09-1.03 (m, 48 H, CH_3 *i*Pr), 0.24 (d, $^2J_{PH}$ = 16.5 Hz, 3 H, CH_3 P-Me).

^{13}C{^{1}H} NMR (CD_2Cl_2, 101 MHz, 300 K): δ (ppm) = 146.6 - 146.4 (C_q Dipp; *ortho*), 142.4 (d, $^2J_{CP}$ = 31 Hz, C_q C=N-P), 131.7 (CH Dipp; *para*), 131.6 (C_q Dipp; *ipso*), 125.5 - 126.1 (C_q Dipp; *meta*), 119.4 (CH N-CH=CH-N), 29.1 - 29.0 (CH *i*Pr), 25.4 - 22.8 (CH_3 *i*Pr), 20.4 (d, $^1J_{CP}$ = 113 Hz, CH_3 P-Me).

^{19}F NMR (CD_2Cl_2, 376 MHz, 300 K): δ (ppm) = -73.67, -78.77.

^{31}P NMR (CD_2Cl_2, 162 MHz, 300 K): δ (ppm) = -11.9 (q, $^2J_{PH}$ = 16 Hz).

HR-MS (ESI): Calculated for $[C_{56}H_{75}N_6O_3PSF_3]^+$ ([**3b**]$^+$): m/z = 999.53056, found: m/z = 999.53159.

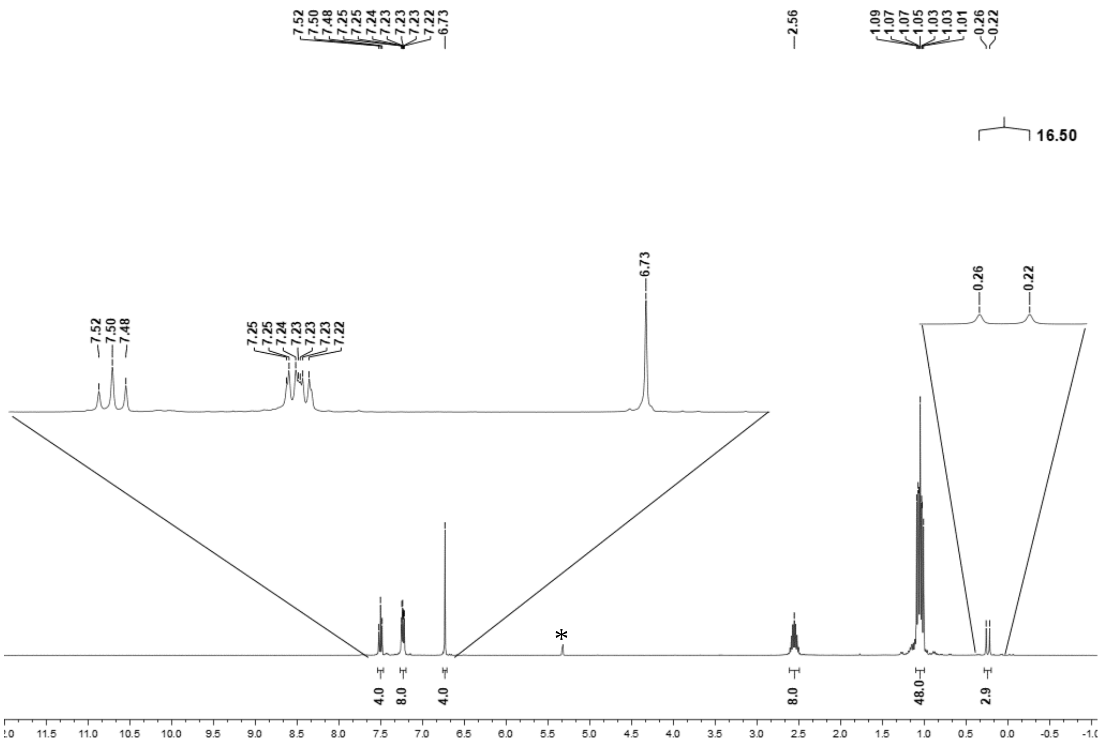

Figure S31: ^{1}H NMR (400 MHz, 300 K) spectrum of [**3b**]OTf in CD_2Cl_2. The asterisk marks the solvent signal.

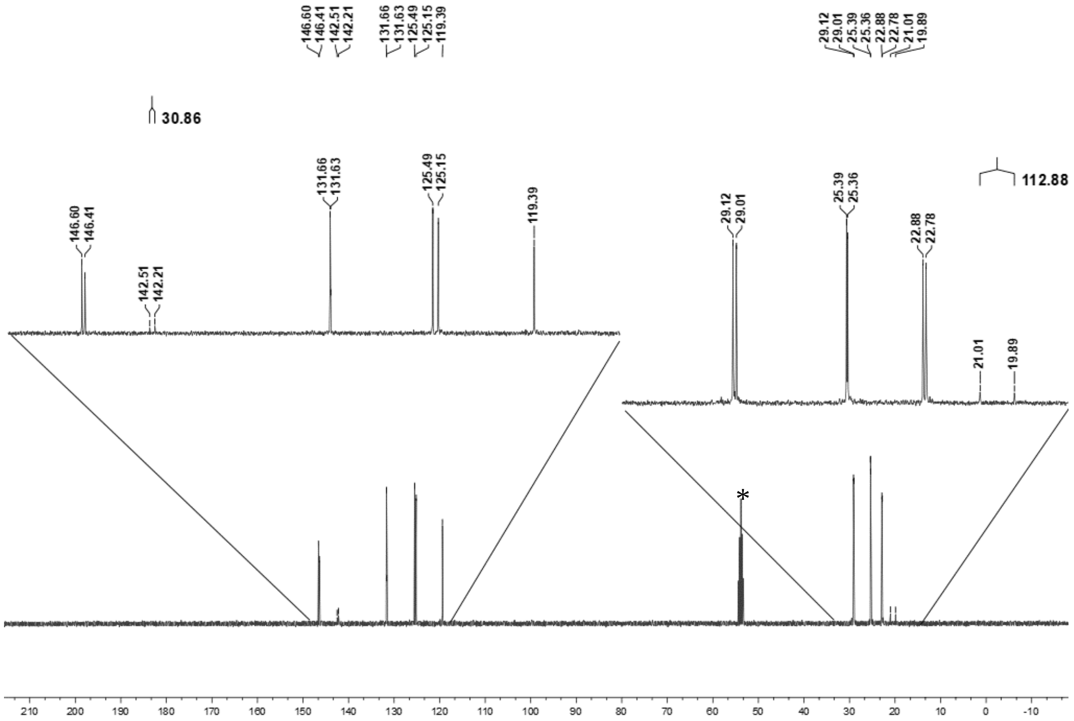

Figure S32: $^{13}C\{^{1}H\}$ NMR (101 MHz, 300 K) spectrum of [**3b**]OTf in CD_2Cl_2. The asterisk marks the solvent signal.

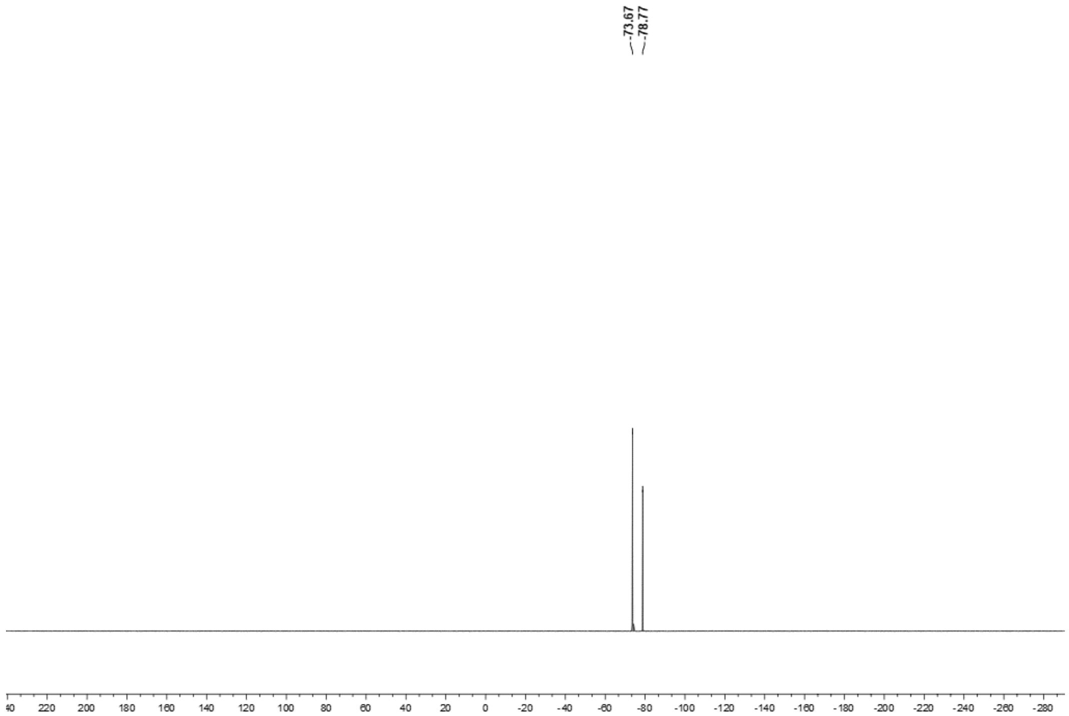

Figure S33: ^{19}F NMR (376 MHz, 300 K) spectrum of [**3b**]OTf in CD_2Cl_2.

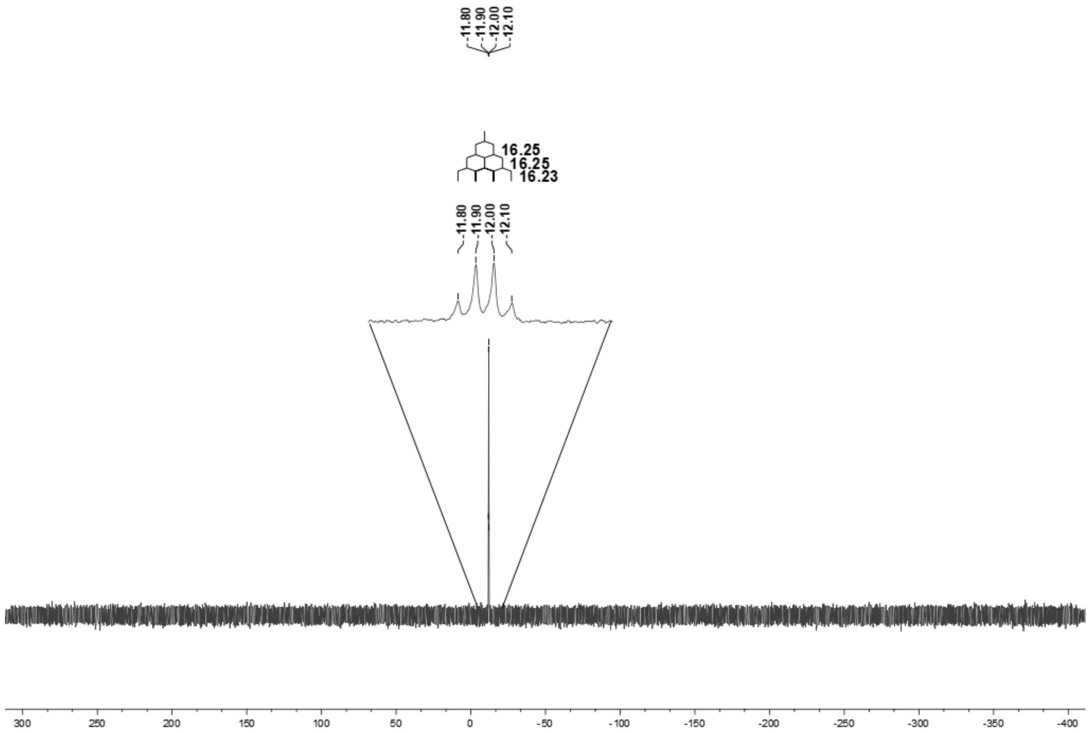

Figure S34: ^{31}P NMR (162 MHz, 300 K) spectrum of [**3b**]OTf in CD_2Cl_2.

1.8 Preparation of [4a]I

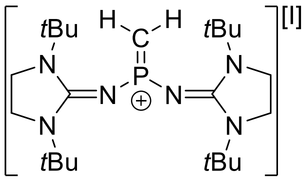

[**3a**]I (2.88 mmol, 2.00 g, 1.00 eq.) was suspended in tetrahydrofuran (50 mL) and cooled to -78°C. While stirring the suspension, a solution of KHMDS (588 mg, 2.95 mmol, 1.02 eq) in tetrahydrofuran (3 mL) was quickly added and the mixture was immediately allowed to warm up to 21 °C. While warming up, the yellow mixture turned white. The mixture was further stirred for 3 h at 21 °C. Afterwards, all volatiles were removed *in vacuo*. The crude product was extracted in CH_2Cl_2 (5 mL). To crystallize the product, diethyl ether was diffused into the solution for 16 h to afford colorless blocks, which were isolated by pipetting off the mother liquor and drying the crystals *in vacuo* at 21 °C for 2 h.

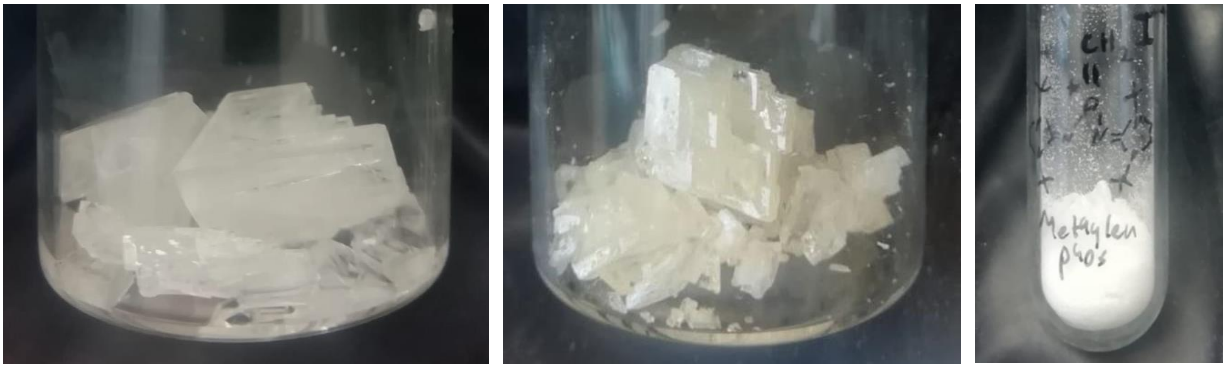

Figure S 35: Two separate batches of [**4a**]I, both around 1.3 g (left and middle); ground [**4a**]I in ampoule (right).

Note: The NMR signals were assigned using 2D-NMR experiments (*vide infra*).

Yield: 1.33 g (2.36 mmol, 82 %).

^{1}H NMR (CD_2Cl_2, 400 MHz, 300 K): δ (ppm) = 3.60 (d, $^5J_{HP}$ = 1.8 Hz 8 H, N-CH_2-CH_2-N), 2.04 (d, $^2J_{HP}$ = 14.9 Hz, 2 H, P=CH_2), 1.46 (s, 36 H, *t*Bu).

^{13}C{^{1}H} NMR (CD_2Cl_2, 101 MHz, 300 K): δ (ppm) = 157.0 (d, $^2J_{CP}$ = 22 Hz, N-C-N), 56.5 (<u>C</u>-Me_3), 43.3 (N-CH_2-CH_2-N), 32.2 (d, $^1J_{CP}$ = 186 Hz, P=CH_2), 28.6 (C-<u>C</u>H_3).

^{31}P NMR (CD_2Cl_2, 162 MHz, 300 K): δ (ppm) = 89.6 (t, $^2J_{PH}$ = 15 Hz).

^{127}I NMR (CD_2Cl_2, 80 MHz, 300 K): δ (ppm) = 77 (br, $w_{1/2}$ = 2500 Hz).

Elemental analysis: Calculated for $C_{23}H_{46}IN_6P$ ([**4a**]I): C 48.93%, H 8.21%, N 14.89%; found: C 48.75%, H 8.16%, N 15.03%.

HR-MS(ESI): Calculated for $[C_{23}H_{46}N_6PI]^+$ ([**4a**+HI]$^+$): m/z = 565.26390, found: m/z = 565.26011.

Melting point: 201°C (decomposition).

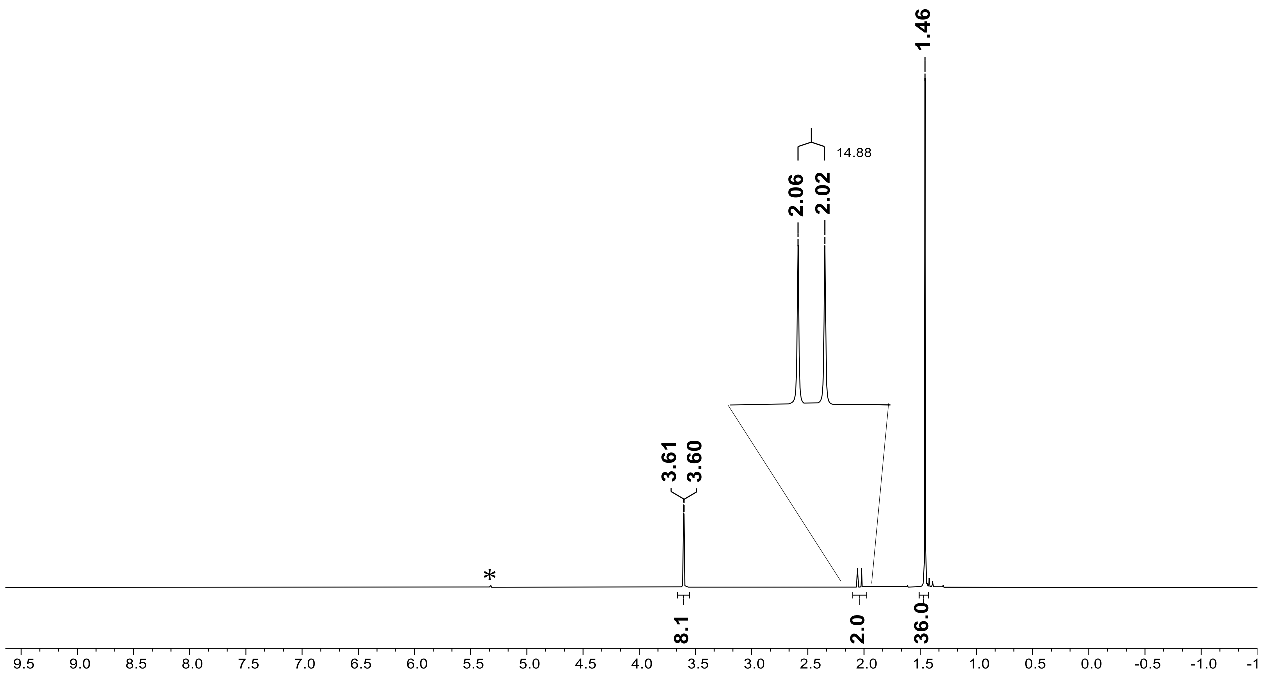

Figure S36: ^{1}H NMR (400 MHz, 300 K) spectrum of [**4a**]I in CD_2Cl_2. The asterisk marks the solvent signal (too weak to be seen at the given amplification level).

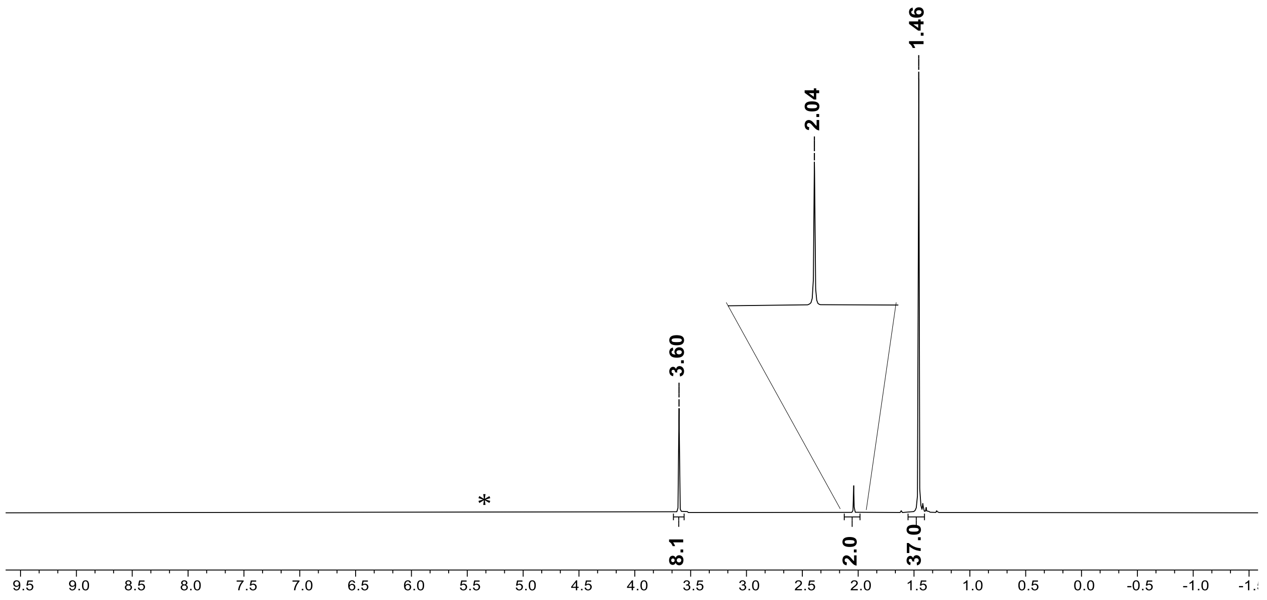

Figure S37: $^{1}H\{^{31}P\}$ NMR (400 MHz, 300 K) spectrum of [**4a**]I in CD_2Cl_2. The asterisk marks the solvent signal (too weak to be seen at the given amplification level).

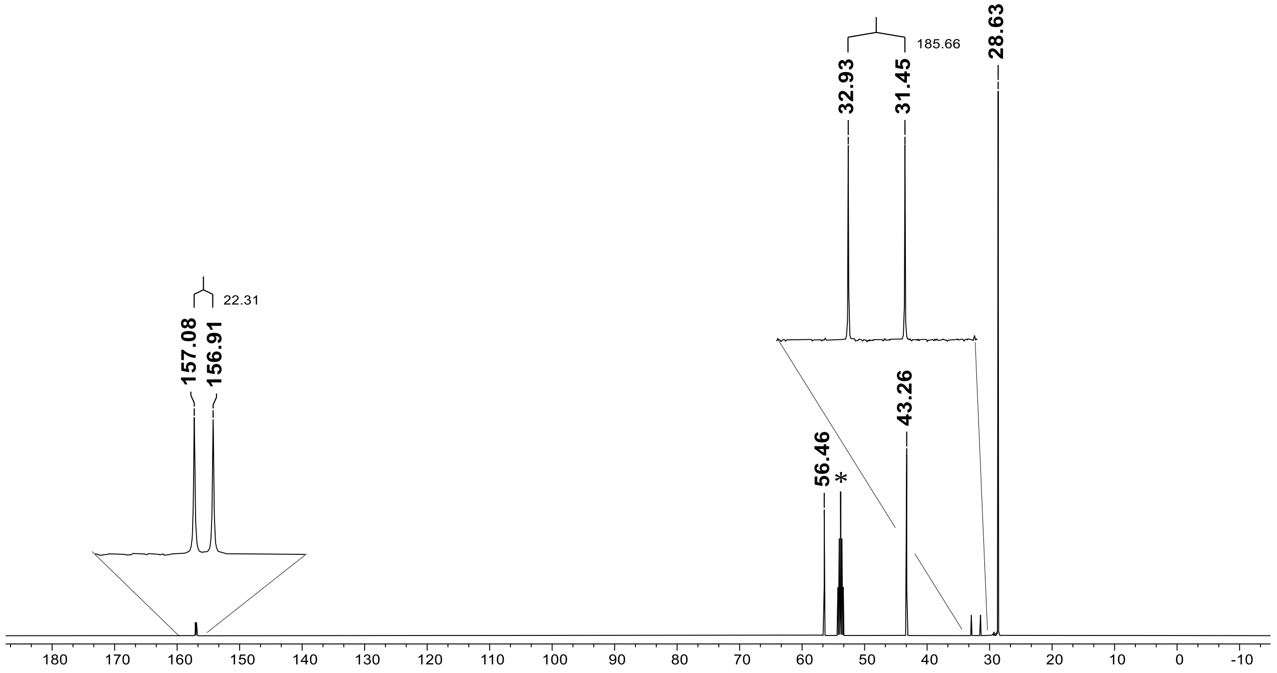

Figure S38: $^{13}C\{^1H\}$ (101 MHz, 300 K) NMR spectrum of [**4a**]I in CD_2Cl_2. The asterisk marks the solvent signal.

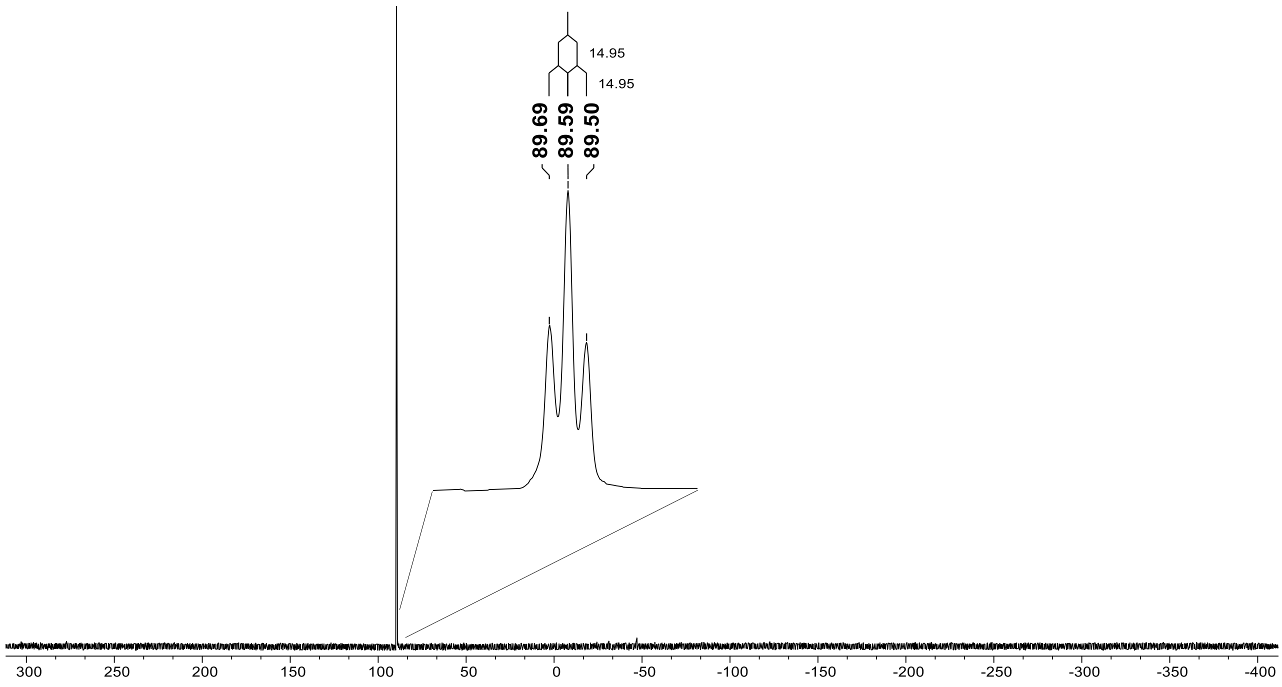

Figure S39: ^{31}P NMR (162 MHz, 300 K) spectrum of [**4a**]I in CD_2Cl_2.

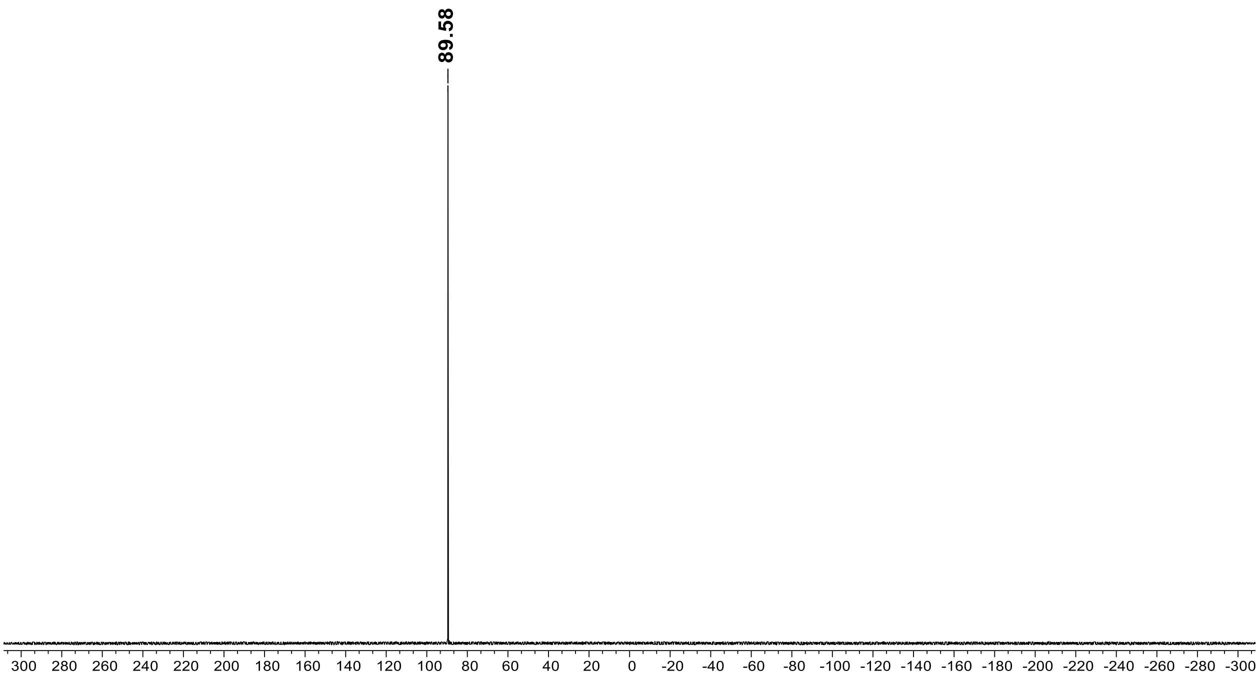

Figure S40: $^{31}P\{^{1}H\}$ (162 MHz, 300 K) NMR spectrum of [**4a**]I in CD_2Cl_2.

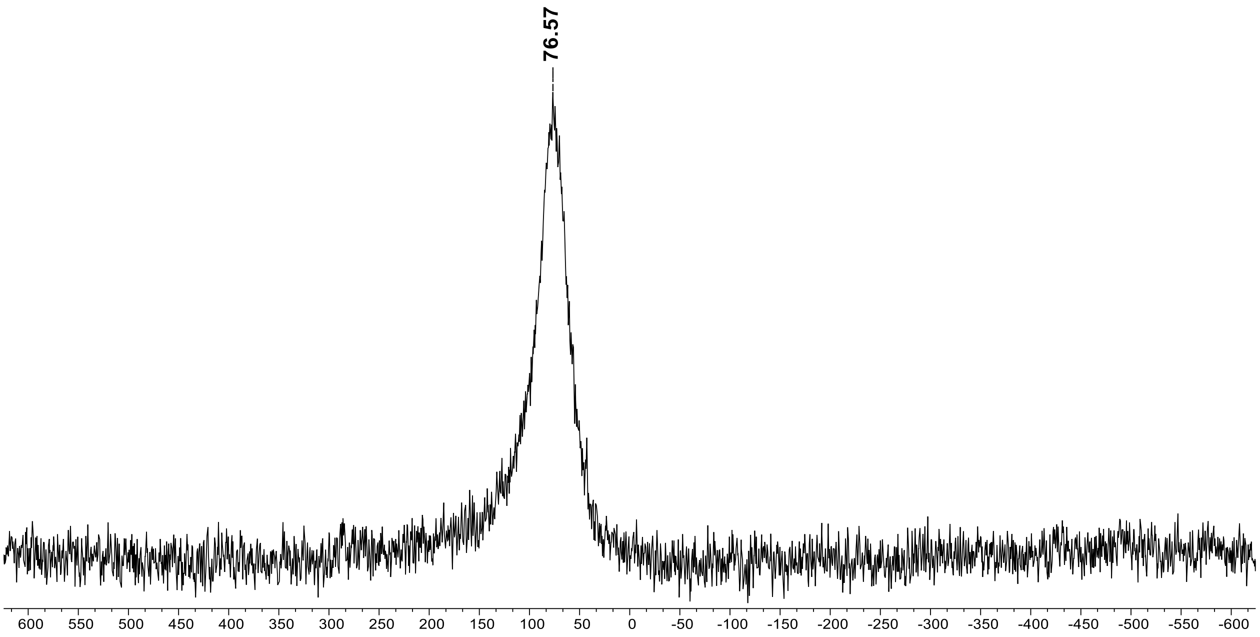

Figure S41: ^{127}I NMR (80 MHz, 300 K) spectrum of [**4a**]I in CD_2Cl_2.

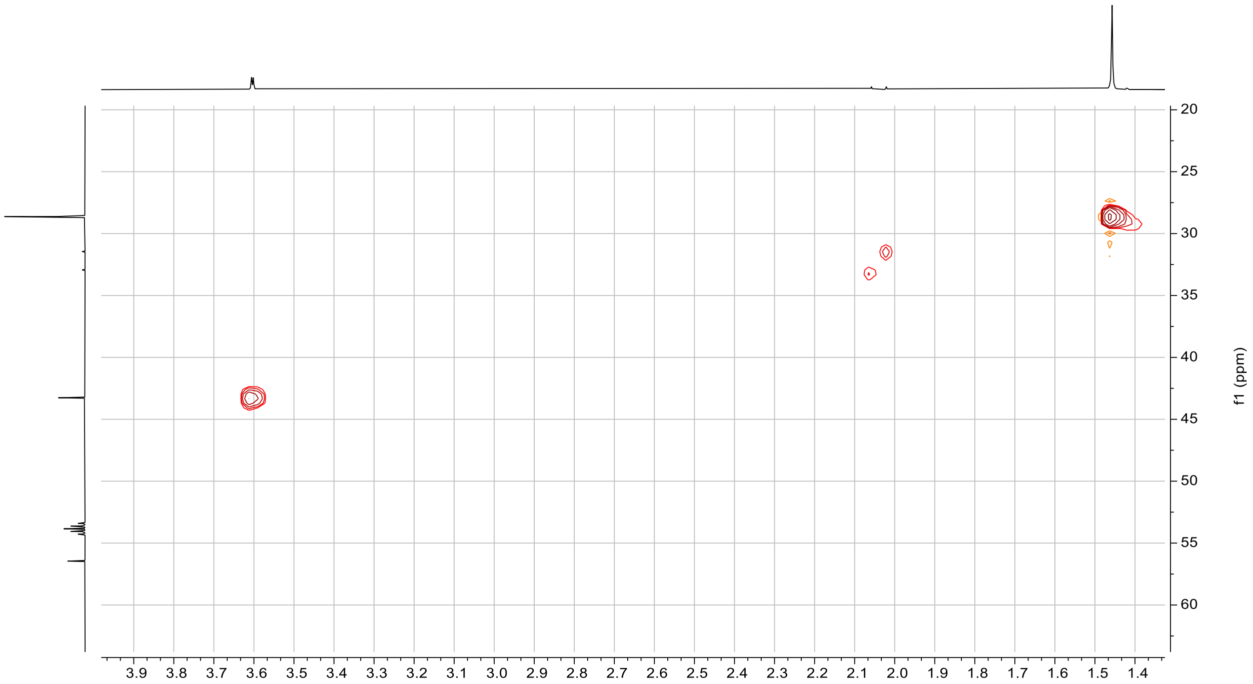

Figure S42: $^1H/^{13}C\{^1H\}$ HSQC NMR spectrum of [**4a**]I in CD_2Cl_2.

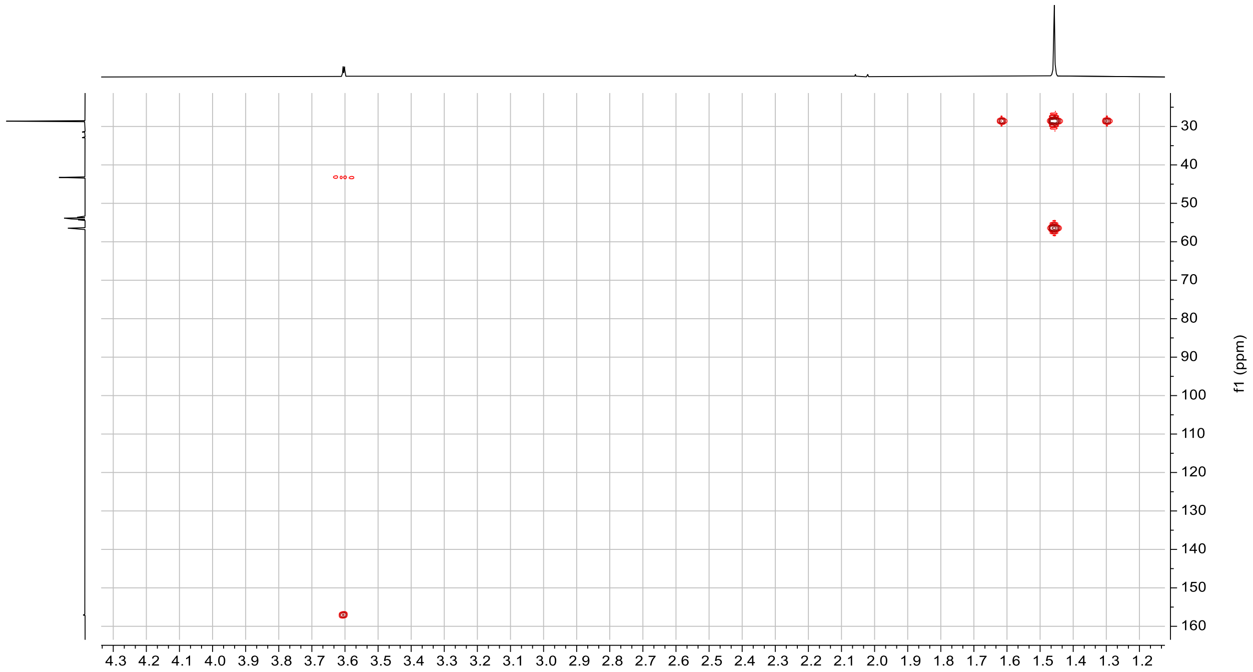

Figure S43: $^1H/^{13}C\{^1H\}$ HMBC NMR spectrum of [**4a**]I in CD_2Cl_2.

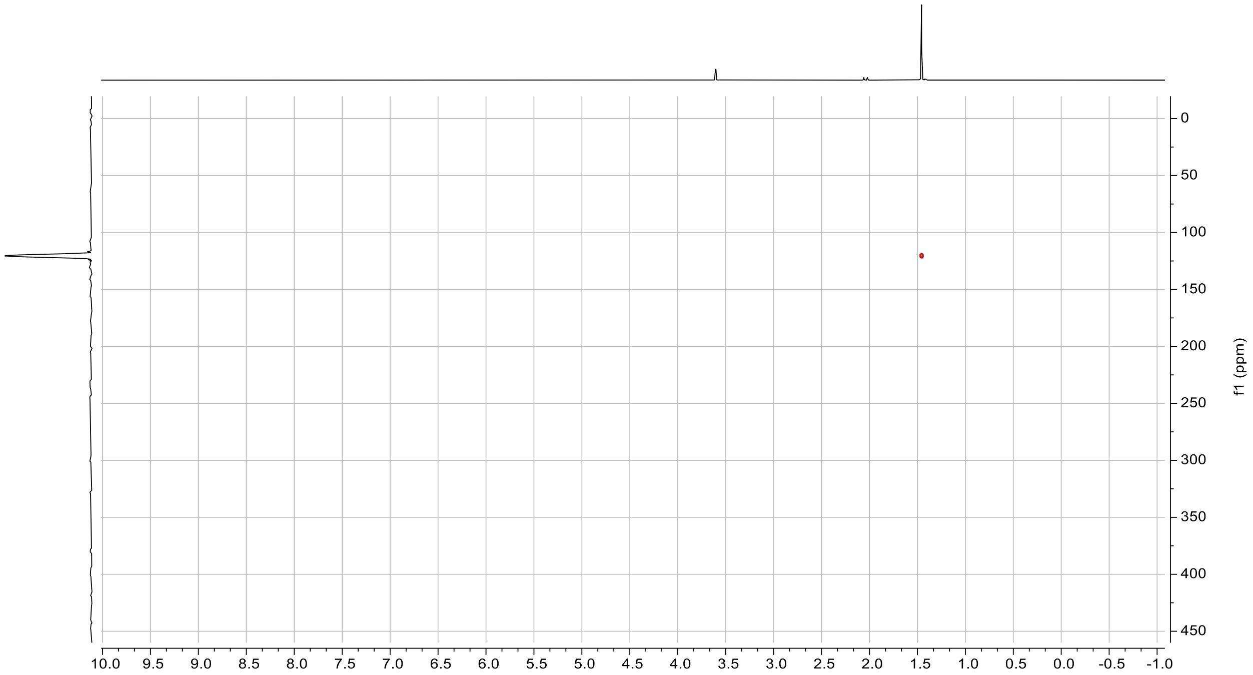

Figure S44: $^{1}H/^{15}N\{^{1}H\}$ HMBC NMR spectrum of [**4a**]I in CD_2Cl_2.

1.9 Preparation of [4a]BArF$_{24}$

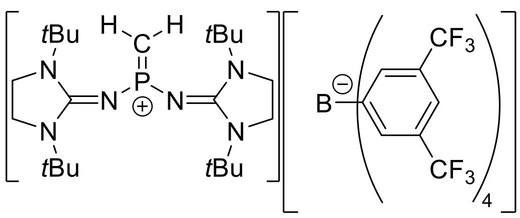

[**4a**][I] (1.58 mmol, 890 mg, 1.00 eq.) was dissolved in CH_2Cl_2 (5 mL) and added to NaBArF$_{24}$ (1.58 mmol, 1.40 g, 1.00 eq.). The mixture was stirred at 21°C for 3 h. Afterwards, the suspension was passed through a glass filter and the precipitate was discarded. All volatiles of the solution were removed *in vacuo*. The product was obtained as a white, powdery solid.

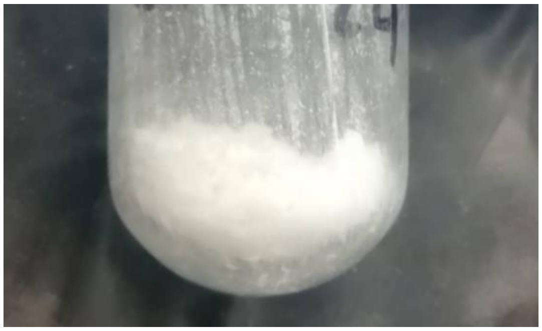

Figure S 45: [**4a**]BArF$_{24}$ in Schlenk flask.

Note: The NMR signals were assigned using 2D-NMR experiments (*vide infra*).

Yield: 1.58 g (1.21 mmol, 77 %).

^{1}H-NMR (CD_2Cl_2, 500 MHz, 300 K): δ (ppm) = 7.72 (m, 8 H, BArF$_{24}$; *ortho*), 7.57 (m, 4 H, BArF$_{24}$; *para*), 3.53 (d, $^{5}J_{HP}$ = 1.7 Hz 8 H, N-CH$_2$-CH$_2$-N), 2.03 (d, $^{2}J_{HP}$ = 15.0 Hz, 2 H, P=CH$_2$), 1.46 (s, 36 H, *t*Bu).

^{1}H{^{31}P}-NMR (CD_2Cl_2, 500 MHz, 300 K): δ (ppm) = 7.72 (m, 8 H, BArF$_{24}$; *ortho*), 7.57 (m, 4 H, BArF$_{24}$; *para*), 3.53 (s, 8 H, N-CH$_2$-CH$_2$-N), 2.03 (s, 2 H, P=CH$_2$), 1.46 (s, 36 H, *t*Bu).

^{13}C{^{1}H}-NMR (CD_2Cl_2, 101 MHz, 300 K): δ (ppm) = 162.2 (q, $^{1}J_{CB}$ = 50 Hz, BArF$_{24}$; *ipso*), 157.0 (d, $^{2}J_{CP}$ = 22 Hz, N-C-N), 135.2 (BArF$_{24}$; *ortho*), 129.3 (qq, $^{2}J_{CF}$ = 32 Hz $^{4}J_{CF}$ = 3 Hz, BArF$_{24}$; *meta*), 125.5 (q, $^{1}J_{CF}$ = 272 Hz, BArF$_{24}$; CF$_3$), 118.0 (sept, $^{3}J_{CB}$ = 4 Hz, BArF$_{24}$; *para*), 56.5 (C-Me$_3$), 43.3 (N-CH$_2$-CH$_2$-N), 32.2 (d, $^{1}J_{CP}$ = 186 Hz, P=CH$_2$), 28.6 (C-CH$_3$).

^{11}B-NMR (CD_2Cl_2, 160 MHz, 300 K): δ (ppm) = -6.6.

^{19}F-NMR (CD_2Cl_2, 471 MHz, 300 K): δ (ppm) = -62.9.

^{31}P-NMR (CD_2Cl_2, 162 MHz, 300 K): δ (ppm) = 88.7 (t, $^{2}J_{PH}$ = 15 Hz).

^{31}P{^{1}H}-NMR (CD_2Cl_2, 202 MHz, 300 K): δ (ppm) = 88.7.

HR-MS(ESI): Calculated for $[C_{23}H_{45}N_6P]^+$ ([**4a**]$^+$): m/z = 437.35161, found: m/z = 437.35215. Calculated for $[C_{46}H_{94}N_{12}OP_2]^{2+}$ ([(**4a**)$_2$+H$_2$O]$^+$): m/z = 437.35161, found: m/z = 437.35215.

Melting point: 129°C (clear liquid).

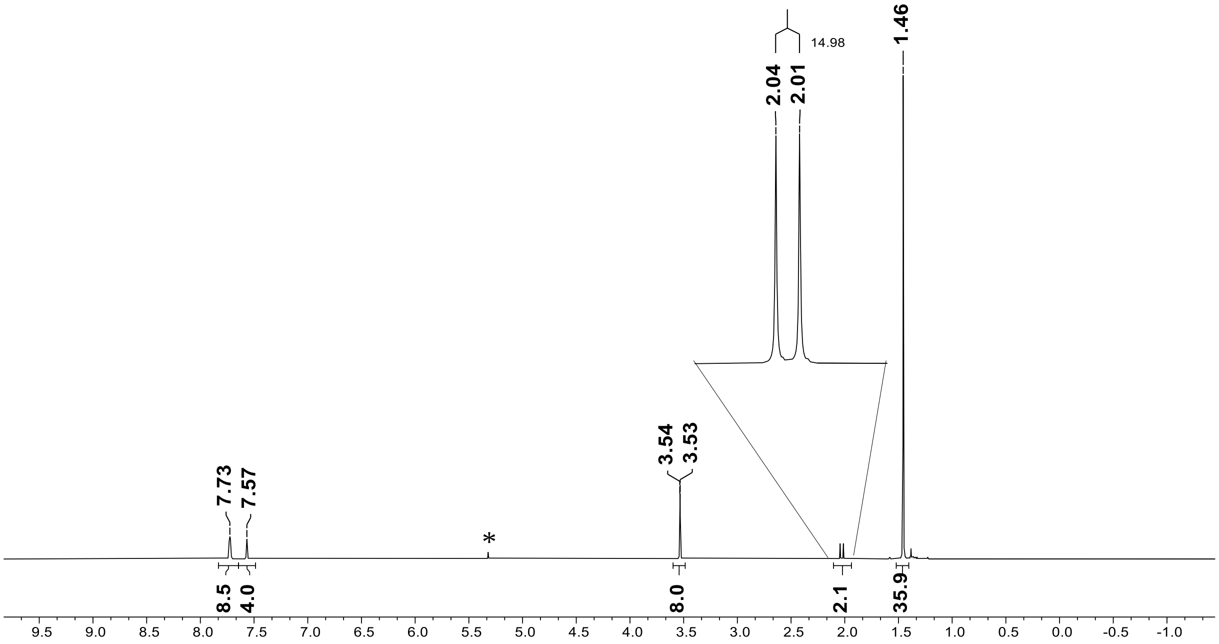

Figure S46: ^{1}H NMR (500 MHz, 300 K) spectrum of [**4a**]$BArF_{24}$ in CD_2Cl_2. The asterisk marks the solvent signal.

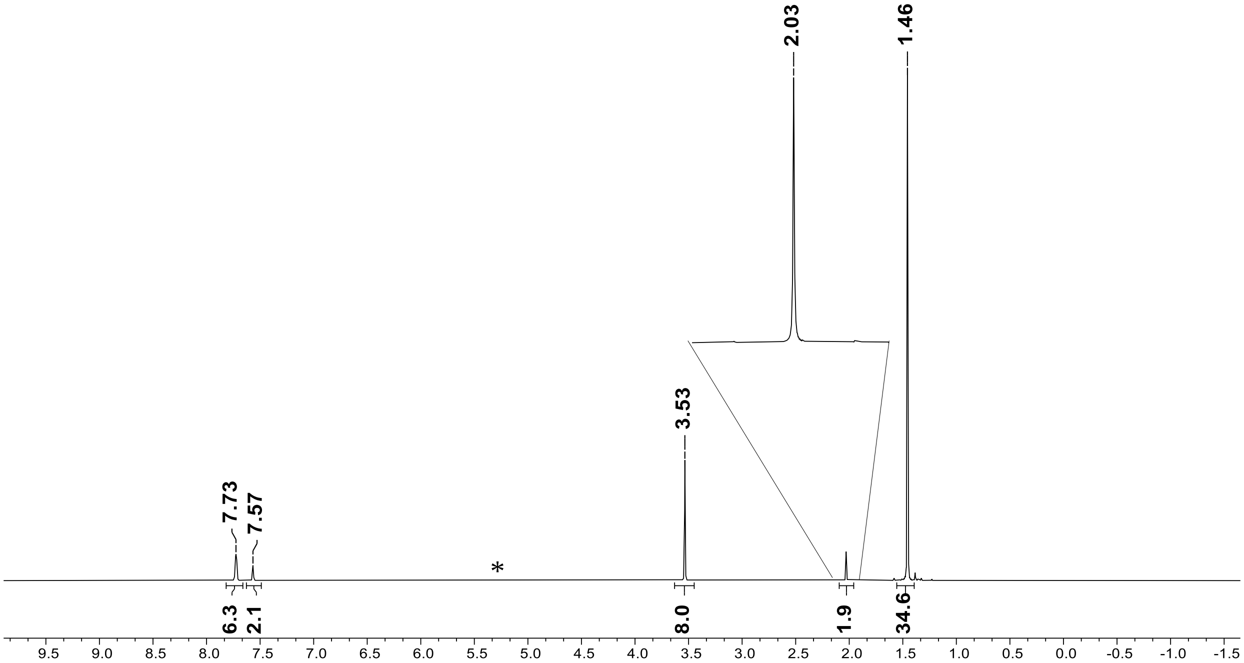

Figure S47: $^{1}H\{^{31}P\}$ NMR (500 MHz, 300 K) spectrum of [**4a**]$BArF_{24}$ in CD_2Cl_2. The asterisk marks the solvent signal (too weak to be seen at the given amplification level).

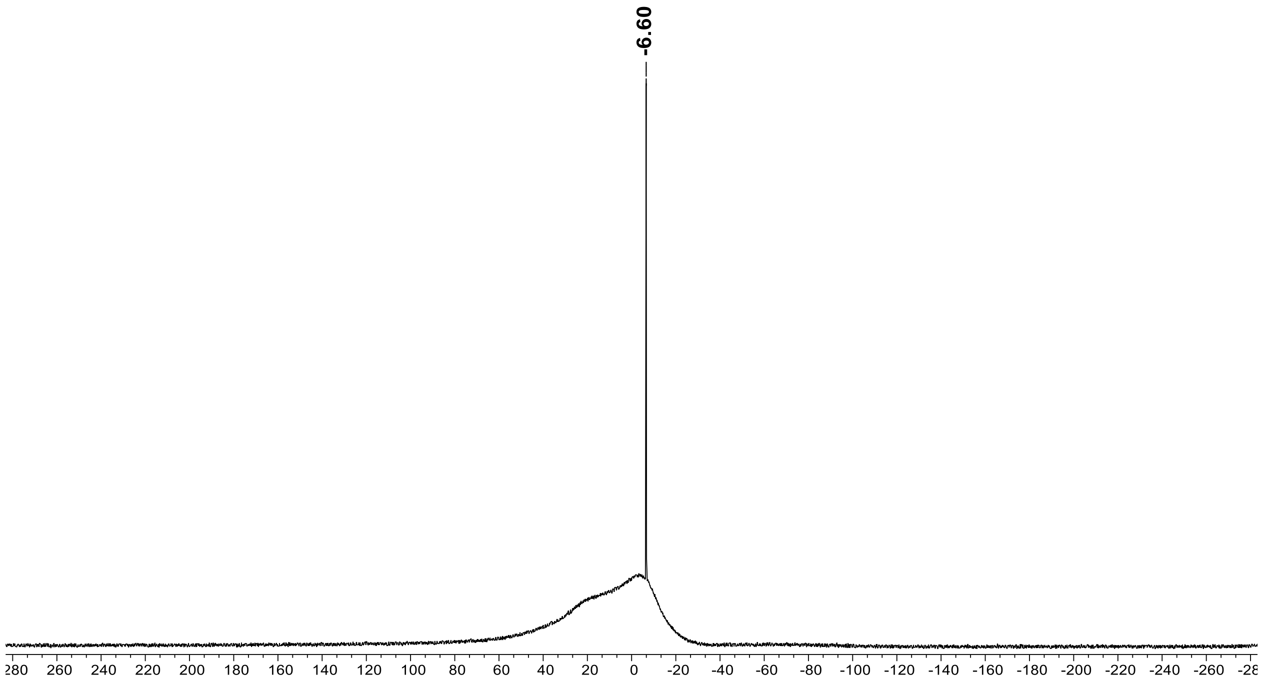

Figure S48: ^{11}B NMR (160 MHz, 300 K) spectrum of [**4a**]BArF$_{24}$ in CD_2Cl_2.

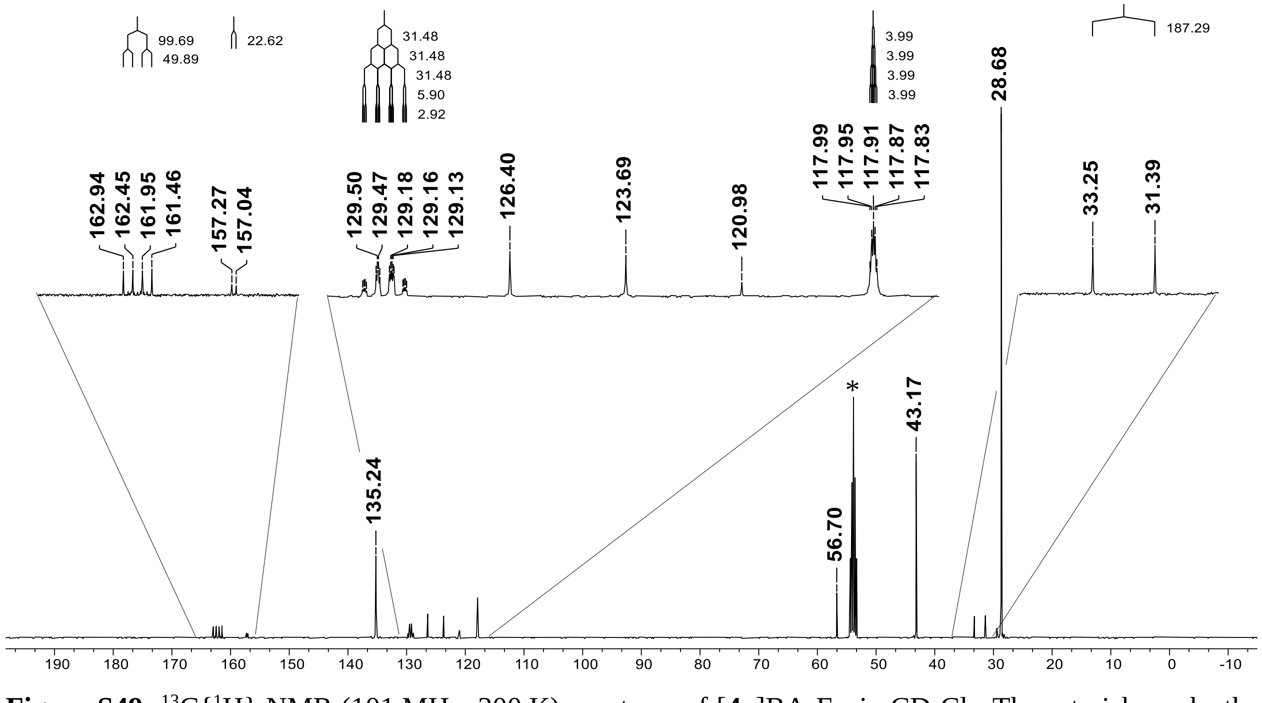

Figure S49: $^{13}C\{^{1}H\}$ NMR (101 MHz, 300 K) spectrum of [**4a**]BArF$_{24}$ in CD_2Cl_2. The asterisk marks the solvent signal.

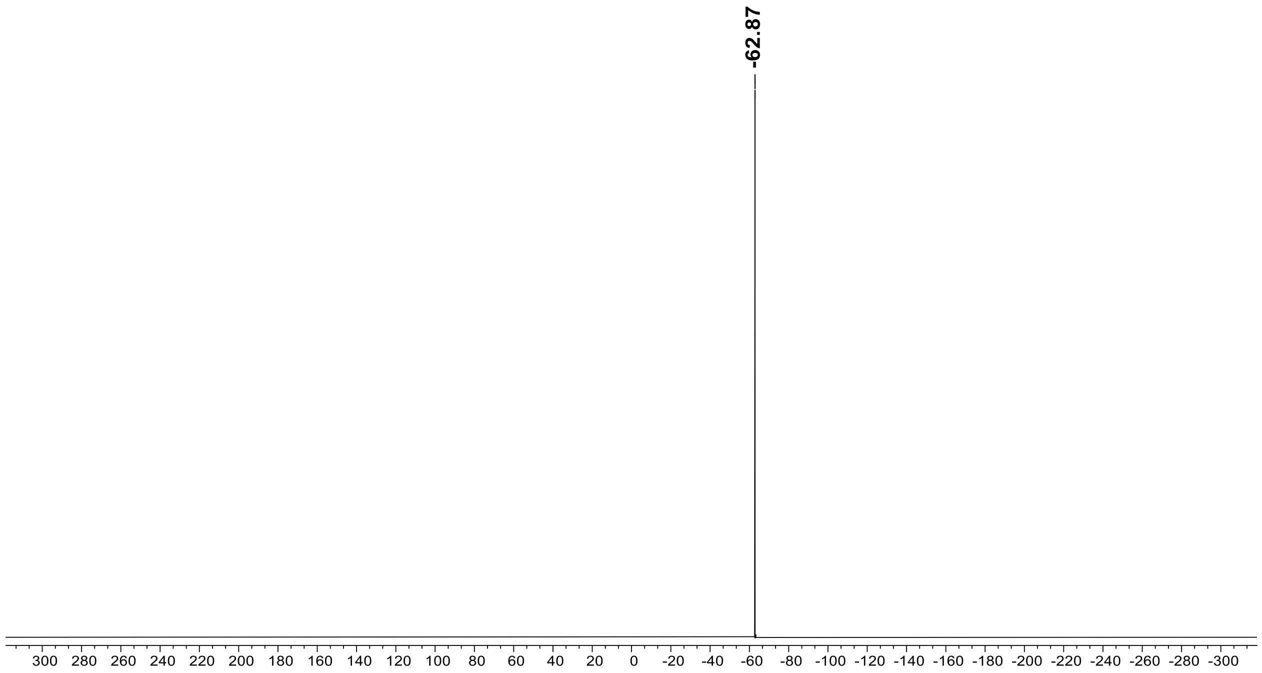

Figure S50: ^{19}F NMR (471 MHz, 300 K) spectrum of [**4a**]BArF$_{24}$ in CD_2Cl_2.

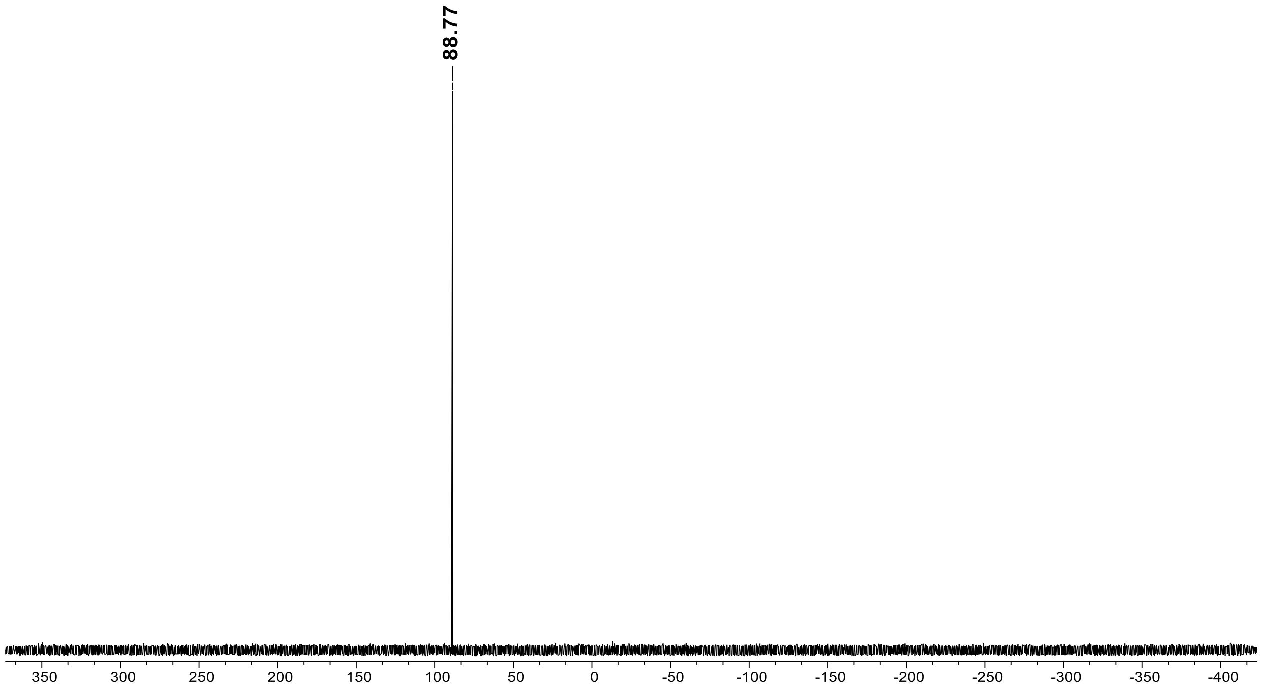

Figure S51: $^{31}P\{^1H\}$ NMR (202 MHz, 300 K) spectrum of [**4a**]BArF$_{24}$ in CD_2Cl_2.

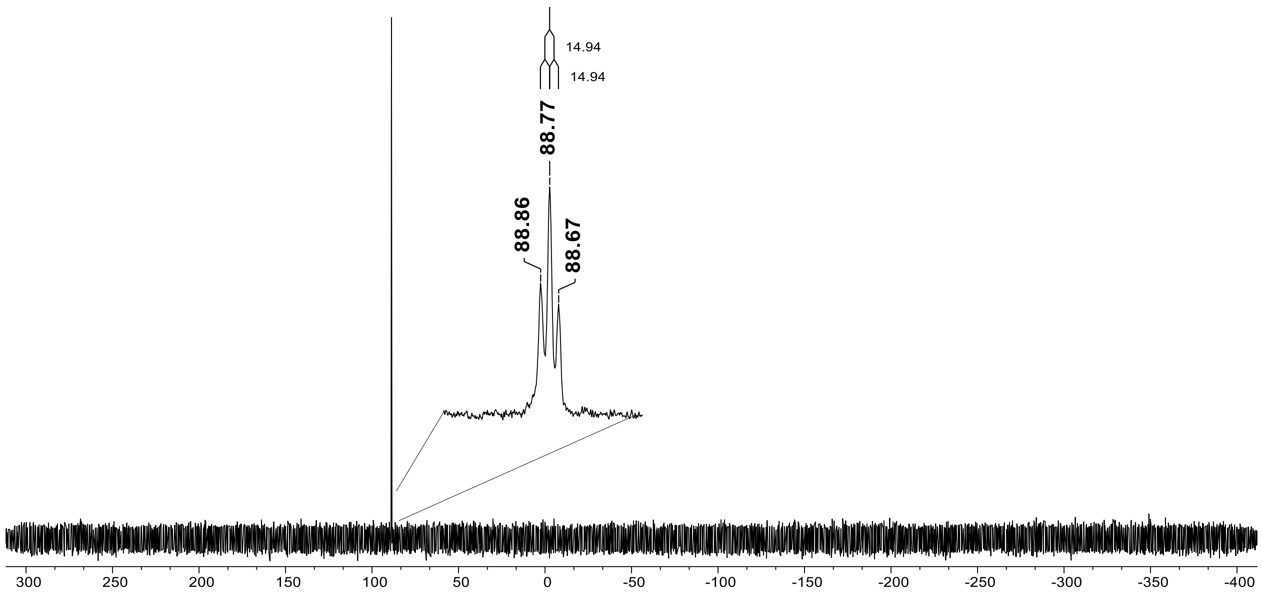

Figure S52: ^{31}P NMR (162 MHz, 300 K) spectrum of [**4a**]BArF$_{24}$ in CD_2Cl_2.

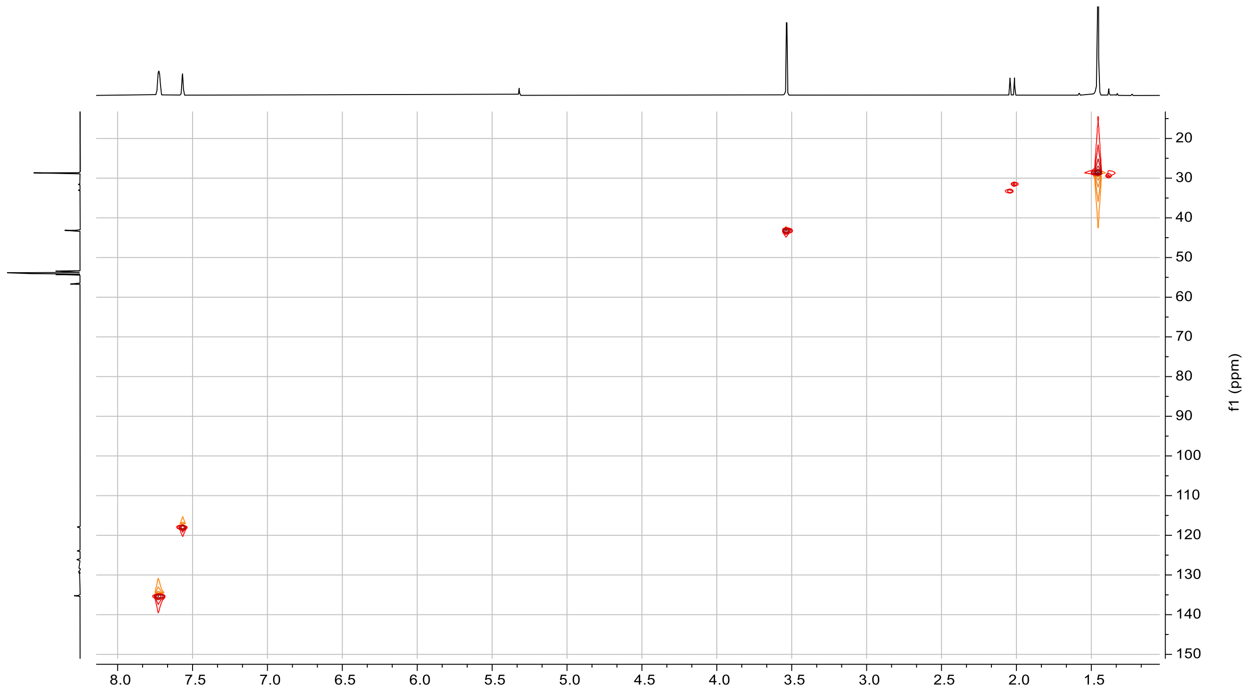

Figure S53: $^{1}H/^{13}C$ HSQC NMR spectrum of [**4a**]BArF$_{24}$ in CD_2Cl_2.

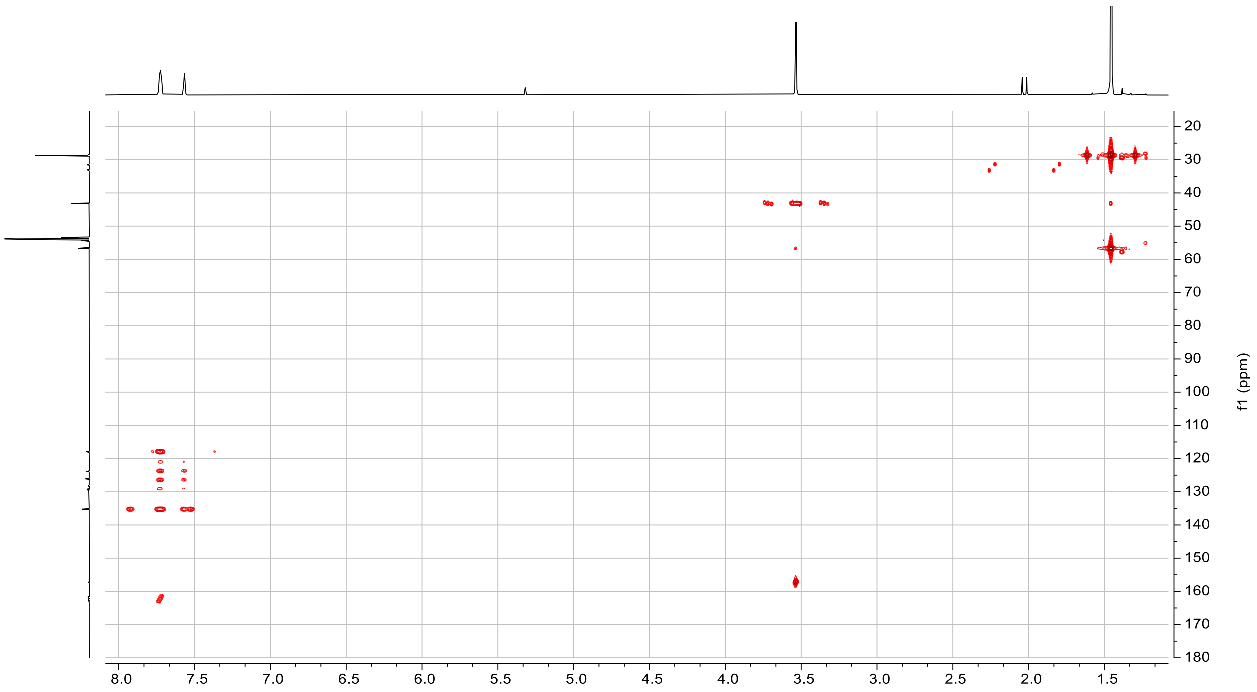

Figure S54: $^{1}H/^{13}C$ HMBC NMR spectrum of [**4a**]BArF24 in CD_2Cl_2.

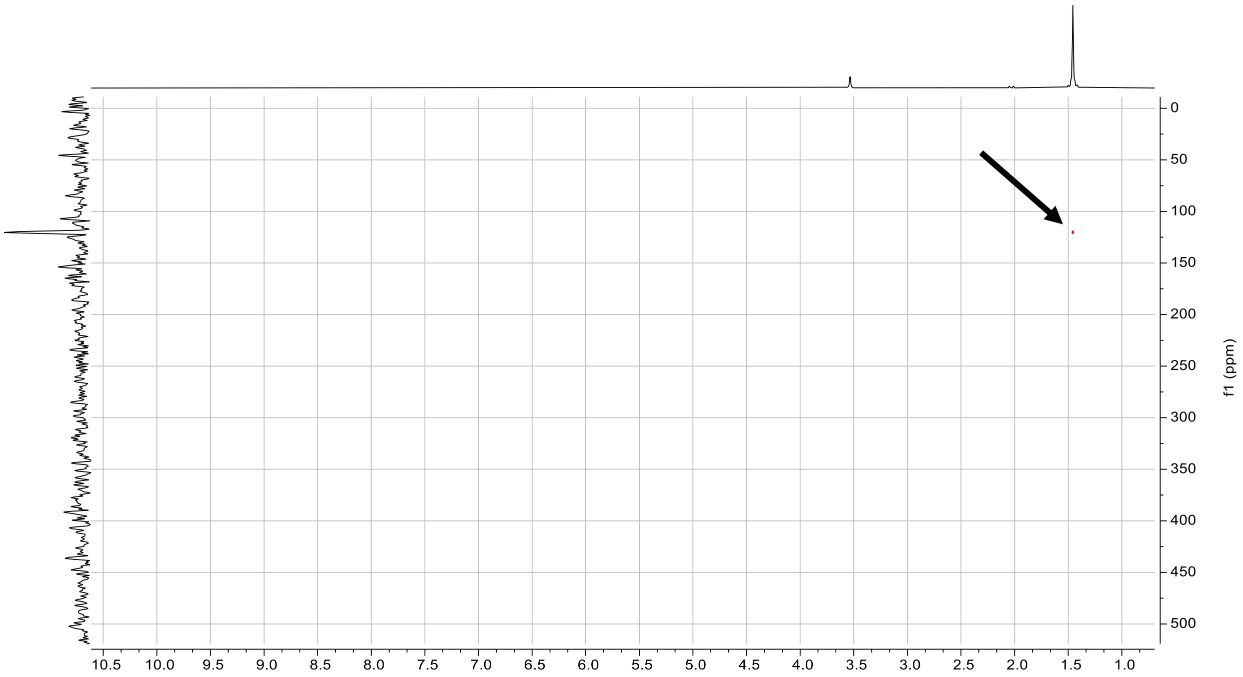

Figure S55: $^{1}H/^{15}N$ HMBC NMR spectrum of [**4a**]BArF24 in CD_2Cl_2. The arrow marks the only signal.

1.10 Preparation of $[4a'](BArF_{24})_2$

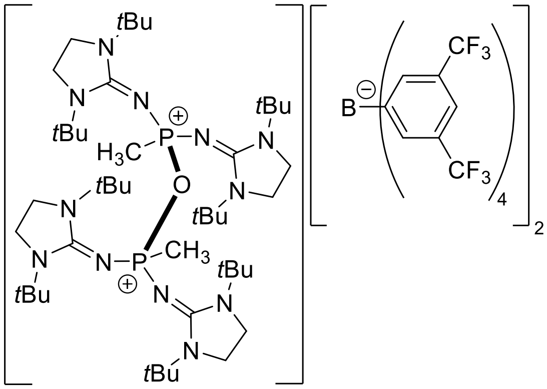

[**4a**]$BArF_{24}$ (25.8 mg, 19.8 µmol, 1.00 eq.) was added into a glass vial and dissolved in diethyl ether (0.3 mL). The vial was sealed with a screw cap. After this step the procedure was continued under ambient air. The vial was opened and deionized water (2.3 mg, 128 µmol, 6.44 eq.) was added to the vial. It was closed again and left undisturbed for 16 h. Colorless crystals formed, which were isolated by removing the mother liquor and washed with diethyl ether (0.1 mL). The crystals were dried *in vacuo*.

Yield: 8.6 mg (3.28 µmol, 33 %).

^{1}H NMR (CD_2Cl_2, 400 MHz, 300 K): δ (ppm) =7.72 (m, 16 H, $BArF_{24}$; *ortho*), 7.56 (m, 8 H, $BArF_{24}$; *para*), 3.53–3.33 (m, 16 H, N-CH_2-CH_2-N), 2.04-2.00 (m, 6 H, CH_3, P-CH_3), 1.39 (s, 72 H, *t*Bu).

^{1}H{^{31}P} NMR (CD_2Cl_2, 400 MHz, 300 K): δ (ppm) =7.72 (m, 16 H, $BArF_{24}$; *ortho*), 7.56 (m, 8 H, $BArF_{24}$; *para*), 3.53–3.33 (m, 16 H, N-CH_2-CH_2-N), 2.02 (s, 6 H, CH_3, P-CH_3), 1.39 (s, 72 H, *t*Bu).

^{13}C{^{1}H} NMR (CD_2Cl_2, 101 MHz, 300 K): δ (ppm) = 162.2 (q, $^{1}J_{CB}$ = 50 Hz, $BArF_{24}$; *ipso*), 157.3 (t, $^{2}J_{CP}$ = 8 Hz, N-C-N), 135.2 ($BArF_{24}$; *ortho*), 129.3 (qq, $^{2}J_{CF}$ = 32 Hz $^{4}J_{CF}$ = 3 Hz, $BArF_{24}$; *meta*), 125.0 (q, $^{1}J_{CF}$ = 272 Hz, $BArF_{24}$; CF_3), 117.9 (sept, $^{3}J_{CB}$ = 4 Hz, $BArF_{24}$; *para*), 57.8 ($\underline{C}$-Me_3), 43.5 (N-CH_2-CH_2-N), 29.4 (C-$\underline{C}H_3$), 19.5 (dd, $^{1}J_{CP}$ = 143 Hz, $^{3}J_{CP}$ = 6 Hz, P-CH_3).

^{11}B NMR (CD_2Cl_2, 128 MHz, 300 K): δ (ppm) = -6.6.

^{31}P NMR (CD_2Cl_2, 162 MHz, 300 K): δ (ppm) = -13.1 – -13.2 (m).

^{31}P{^{1}H} NMR (CD_2Cl_2, 162 MHz, 300 K): δ (ppm) = -13.2.

HR-MS(ESI): Calculated for $[C_{46}H_{94}N_{12}OP_2]^{2+}$ ([**4a'**]$^{2+}$): m/z = 446.3569, found: m/z = 446.3572.

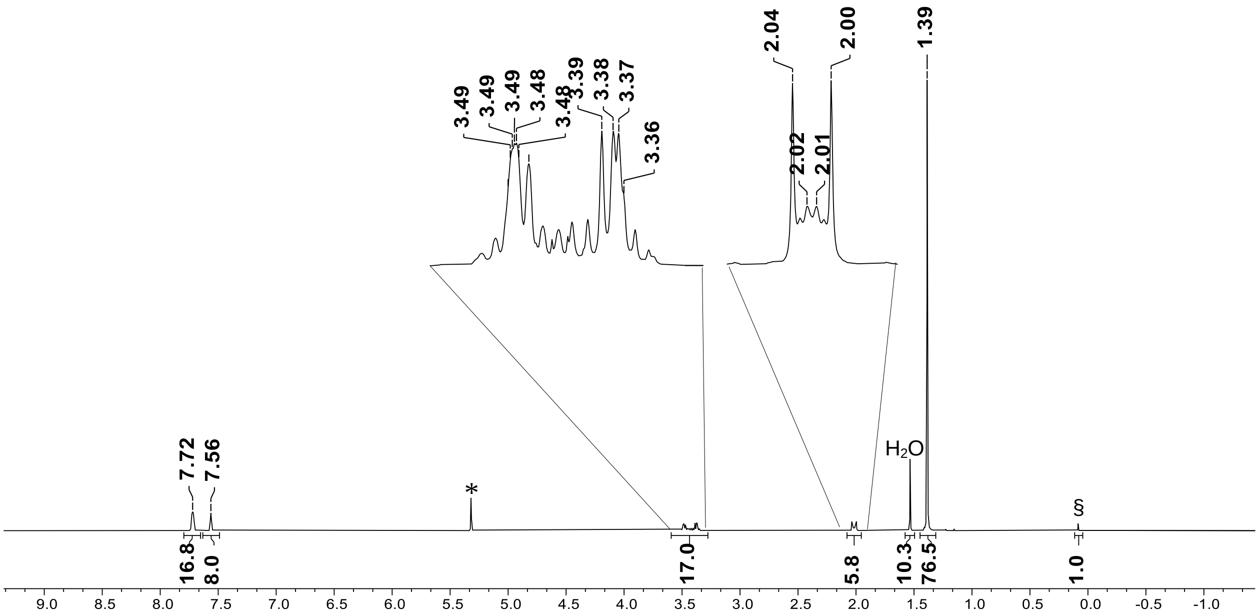

Figure S56: ^{1}H NMR (400 MHz, 300 K) spectrum of [**4a’**]($BArF_{24}$)$_2$ in CD_2Cl_2. The asterisk marks the solvent signal. The section sign (§) marks the signal corresponding to vacuum grease.

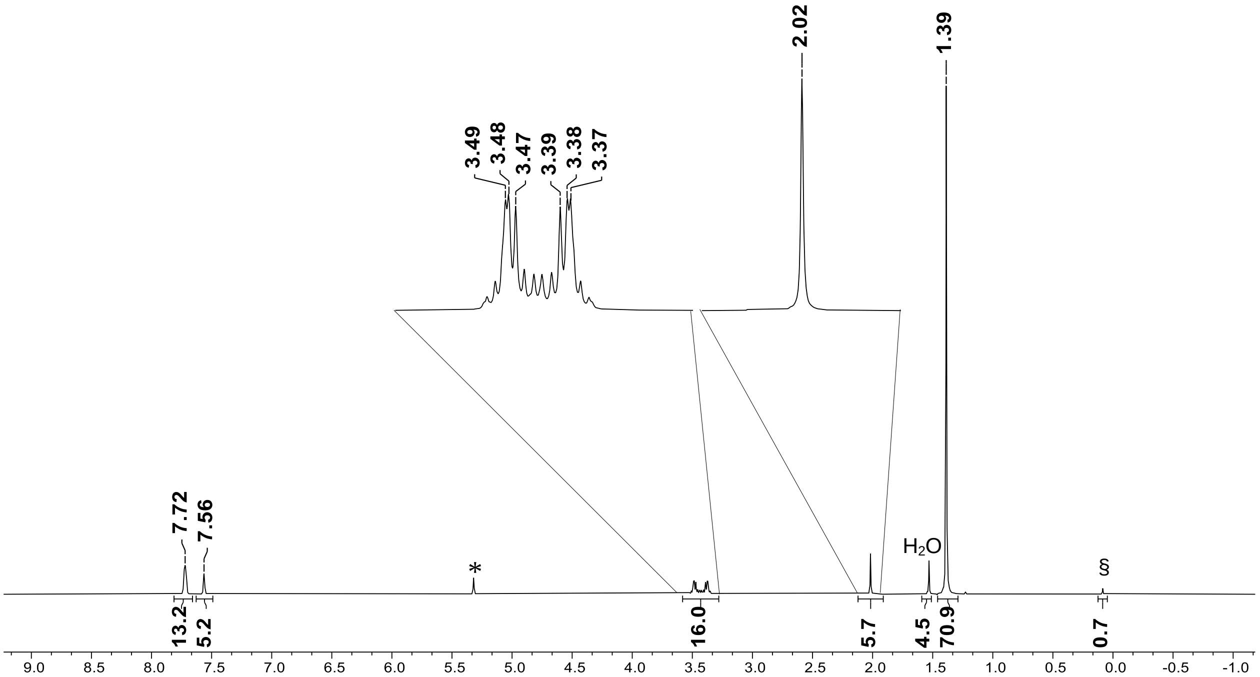

Figure S57: $^{1}H\{^{31}P\}$ NMR (400 MHz, 300 K) spectrum of [**4a’**]($BArF_{24}$)$_2$ in CD_2Cl_2. The asterisk marks the solvent signal. The section sign marks the signal corresponding to vacuum grease.

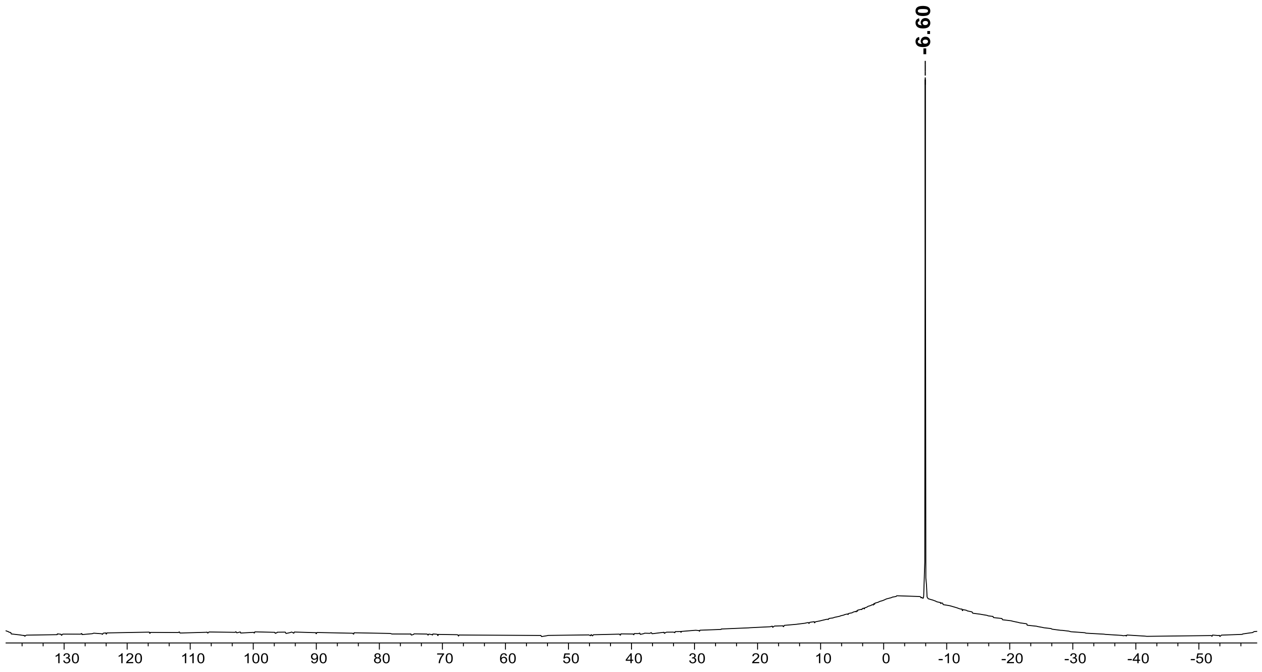

Figure S58: ^{11}B NMR (128 MHz, 300 K) spectrum of [**4a'**]($BArF_{24}$)$_2$ in CD_2Cl_2.

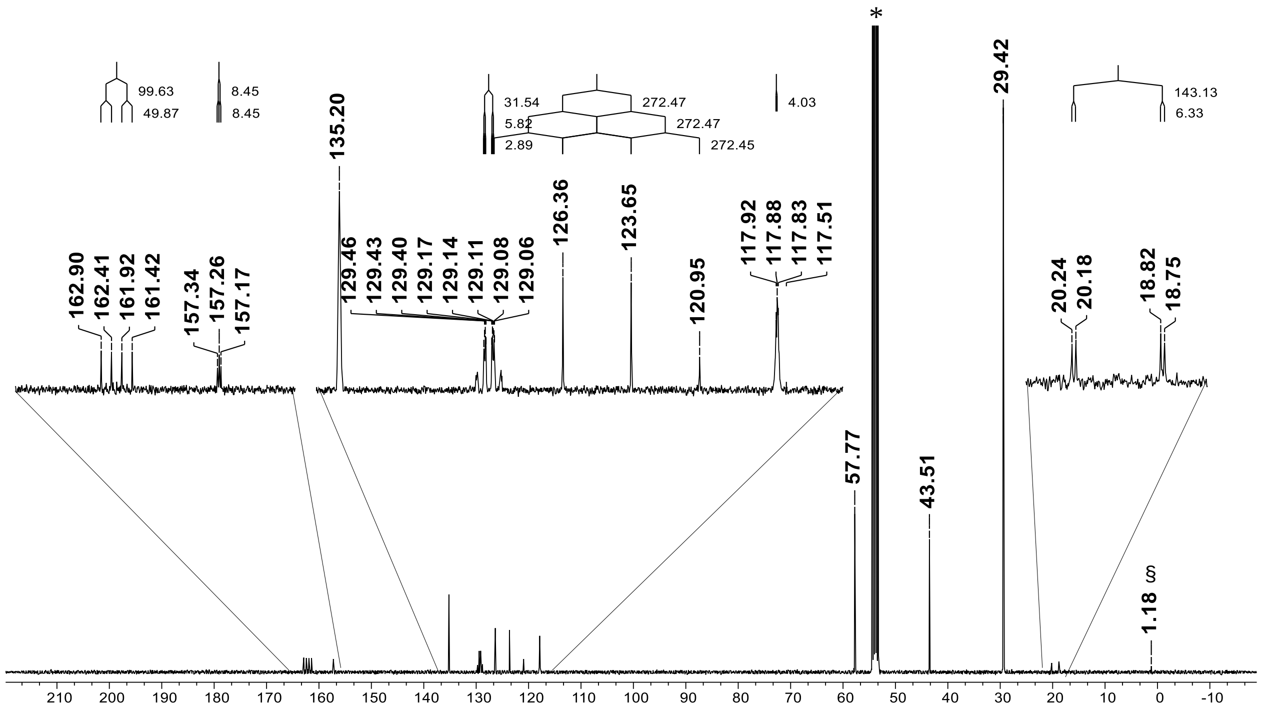

Figure S59: ^{13}C{^{1}H} NMR spectrum of [**4a'**]($BArF_{24}$)$_2$ in CD_2Cl_2. The asterisk marks the solvent signal. The solvent signal is cut off due to low sample signal intensity. The section sign marks the signal corresponding to vacuum grease.

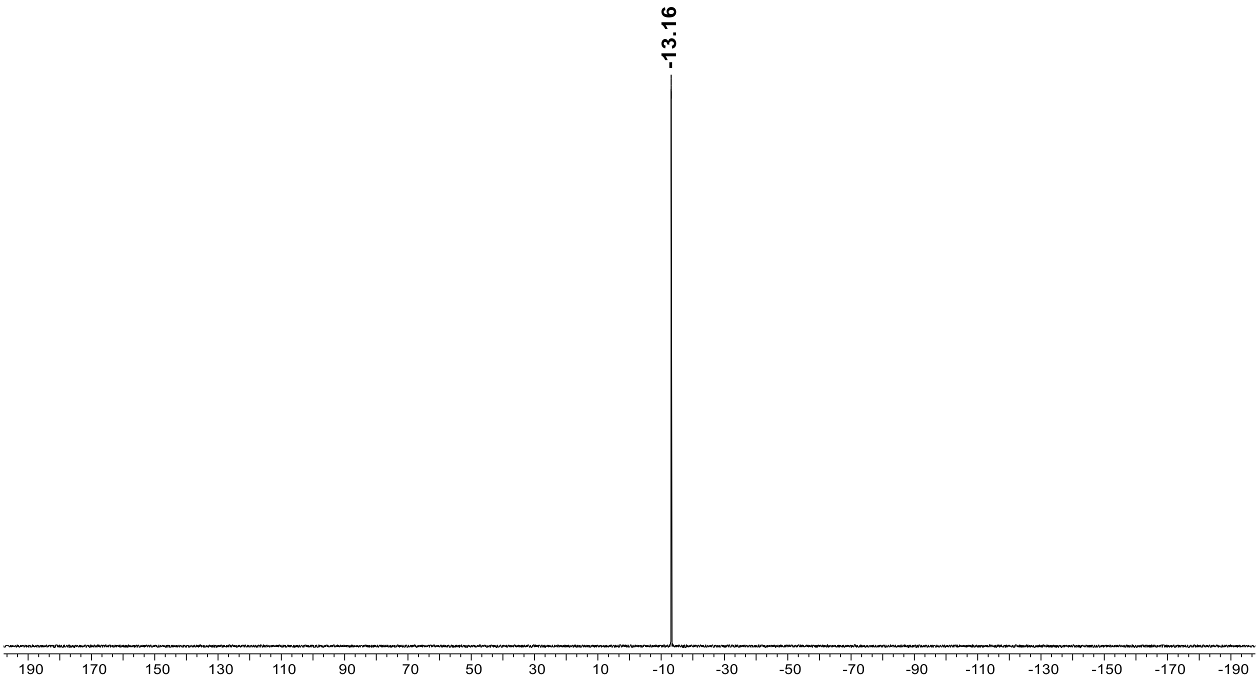

Figure S60: $^{31}P\{^{1}H\}$ NMR (162 MHz, 300 K) spectrum of [**4a'**]$(BArF_{24})_2$ in CD_2Cl_2.

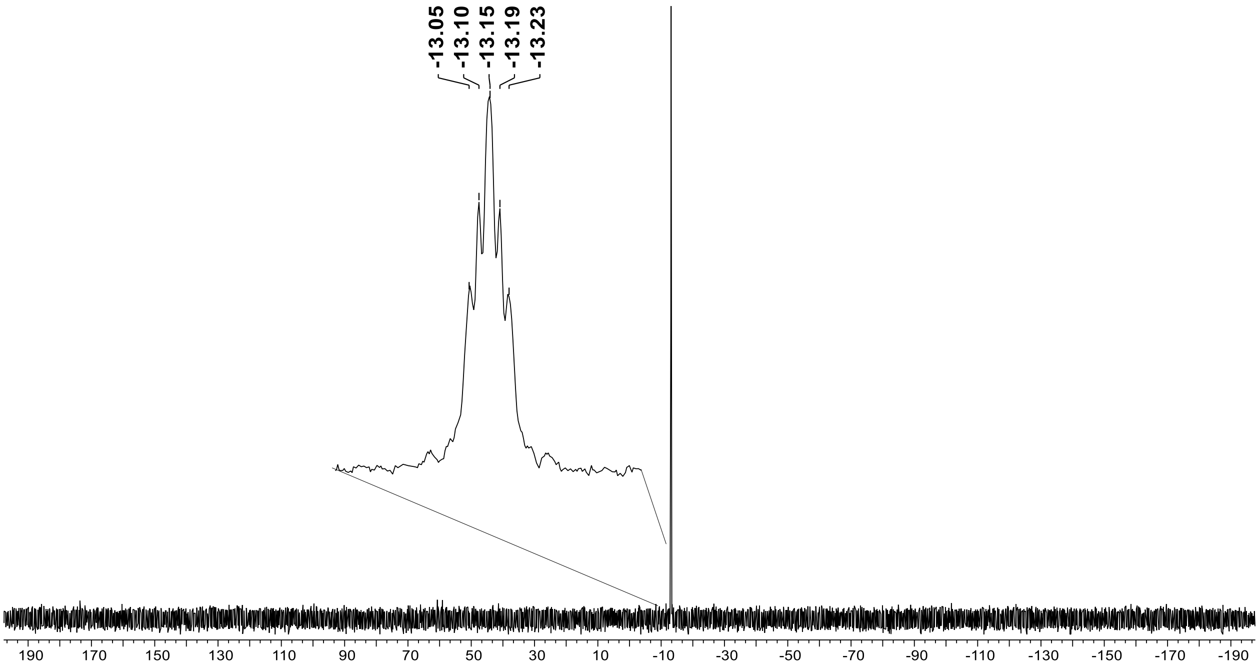

Figure S61: ^{31}P NMR (162 MHz, 300 K) spectrum of [**4a'**]$(BArF_{24})_2$ in CD_2Cl_2.

1.11 Characterization data of [4a'*](BArF24)2

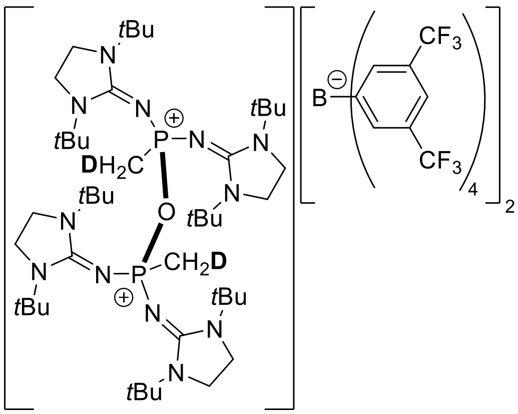

[**4a**][BArF$_{24}$] (82.6 mg, 63.5 µmol, 1.00 eq.) was added into a glass vial and dissolved in diethyl ether (0.3 mL). The vial was sealed with a screw cap. After this step the procedure was continued under ambient air. The vial was opened and D_2O (6.6 mg, 33 µmol, 5.2 eq.) was added to the vial. It was closed again and left for 2 days. The vial was opened and the volatiles were left to evaporate for 16 h. The residue was redissolved in diethyl ether (0.5 mL). After the vial was capped and shaken, two layers have formed. The top layer was pipetted off and discarded. The bottom layer was dried *in vacuo*.

Yield: 66.6 mg (25.4 µmol, 80 %).

^{1}H NMR (CD_2Cl_2, 400 MHz, 300 K): δ (ppm) =7.75 (m, 16 H, BArF$_{24}$; *ortho*), 7.58 (m, 8 H, BArF$_{24}$; *para*), 3.53–3.33 (m, 16 H, N-CH_2-CH_2-N), 2.05-1.99 (m, 4 H, CH_3, P-CH_2D), 1.39 (s, 72 H, *t*Bu).

^{1}H{^{31}P} NMR (CD_2Cl_2, 400 MHz, 300 K): δ (ppm) =7.75 (m, 16 H, BArF$_{24}$; *ortho*), 7.59 (m, 8 H, BArF$_{24}$; *para*), 3.53–3.33 (m, 16 H, N-CH_2-CH_2-N), 2.03-2.02 (m, 4 H, CH_3, P- CH_2D), 1.39 (s, 72 H, *t*Bu).

^{13}C{^{1}H} NMR (CD_2Cl_2, 101 MHz, 300 K): δ (ppm) = 162.2 (q, $^{1}J_{CB}$ = 50 Hz, BArF$_{24}$; *ipso*), 157.3 (t, $^{2}J_{CP}$ = 8 Hz, N-C-N), 135.2 (BArF$_{24}$; *ortho*), 129.3 (qq, $^{2}J_{CF}$ = 32 Hz $^{4}J_{CF}$ = 3 Hz, BArF$_{24}$; *meta*), 125.0 (q, $^{1}J_{CF}$ = 272 Hz, BArF$_{24}$; CF_3), 117.9 (sept, $^{3}J_{CB}$ = 4 Hz, BArF$_{24}$; *para*), 57.8 (C-Me$_3$), 43.5 (N-CH_2-CH_2-N), 29.4 (C-CH_3), 19.5 (dtd, $^{1}J_{CP}$ = 143 Hz, $^{1}J_{CD}$ = 20 Hz, $^{3}J_{CP}$ = 7 Hz, P-CH_2D).

^{11}B NMR (CD_2Cl_2, 128 MHz, 300 K): δ (ppm) = -6.6.

^{19}F NMR (CD_2Cl_2, 377 MHz, 300 K): δ (ppm) = -62.8.

^{31}P NMR (CD_2Cl_2, 162 MHz, 300 K): δ (ppm) = -12.99–-13.09 (m).

^{31}P{^{1}H} NMR (CD_2Cl_2, 162 MHz, 300 K): δ (ppm) = -13.1.

HR-MS(ESI): Calculated for $[C_{46}D_2H_{92}N_{12}OP_2]^{2+}$ ([**4a'***]$^{2+}$): m/z = 447.3632, found: m/z = 447.3625.

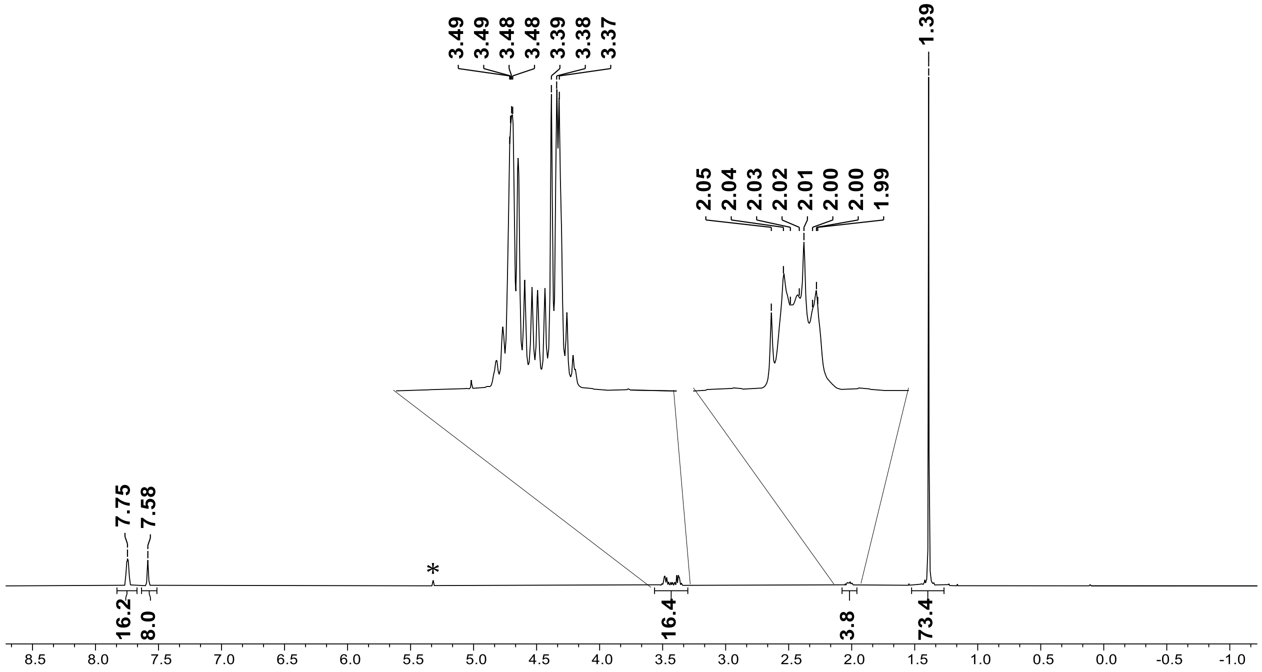

Figure S62: ^{1}H NMR (400 MHz, 300 K) spectrum of [**4a'***]($BArF_{24}$)$_2$ in CD_2Cl_2. The asterisk marks the solvent signal.

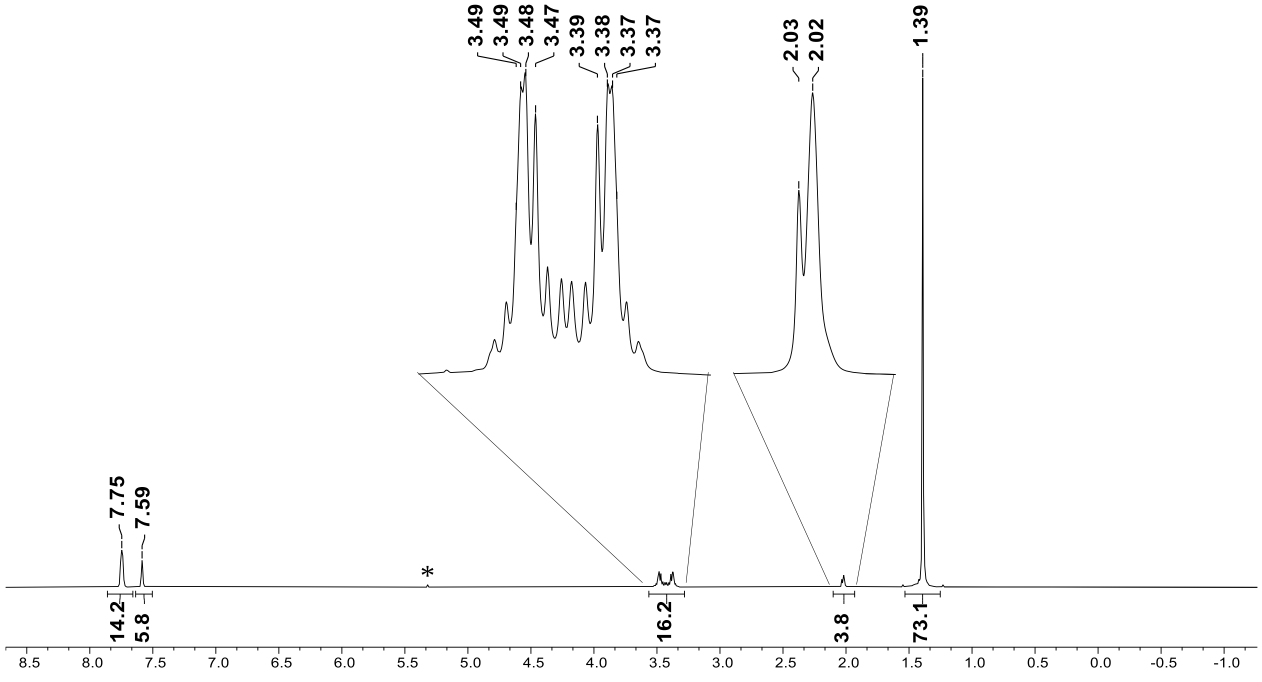

Figure S63: $^{1}H\{^{31}P\}$ NMR (400 MHz, 300 K) spectrum of [**4a'***]($BArF_{24}$)$_2$ in CD_2Cl_2. The asterisk marks the solvent signal.

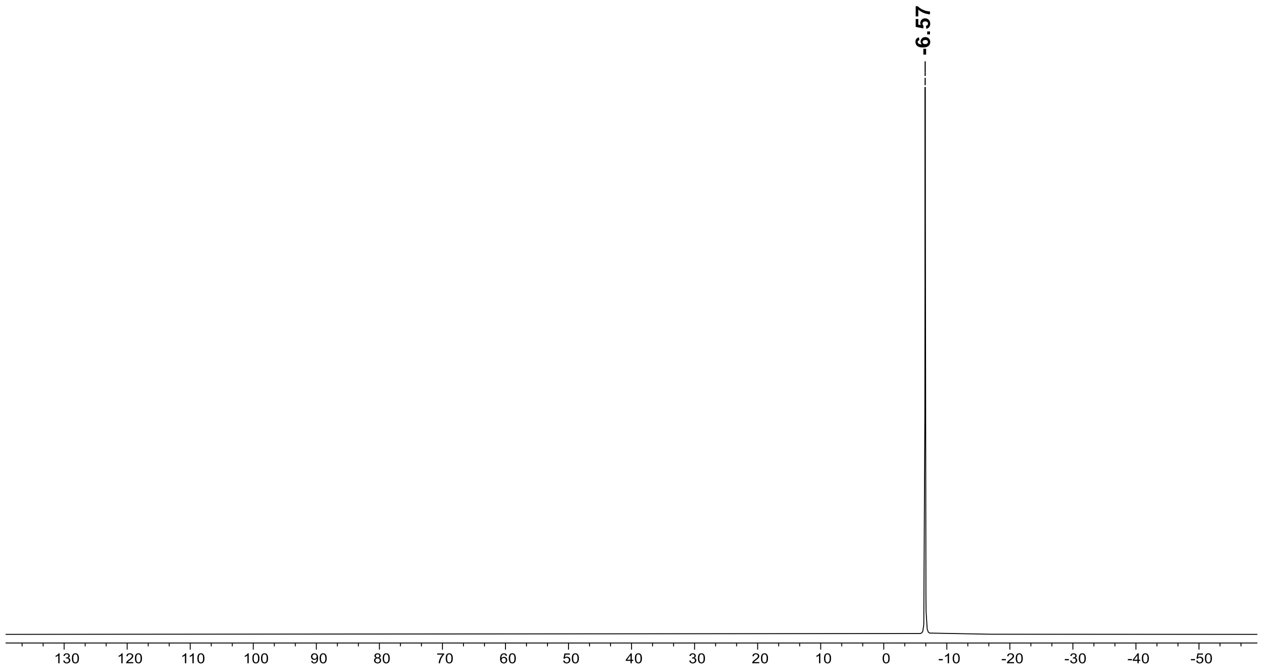

Figure S64: ^{11}B NMR (128 MHz, 300 K) spectrum of [**4a'***]($BArF_{24}$)$_2$ in CD_2Cl_2.

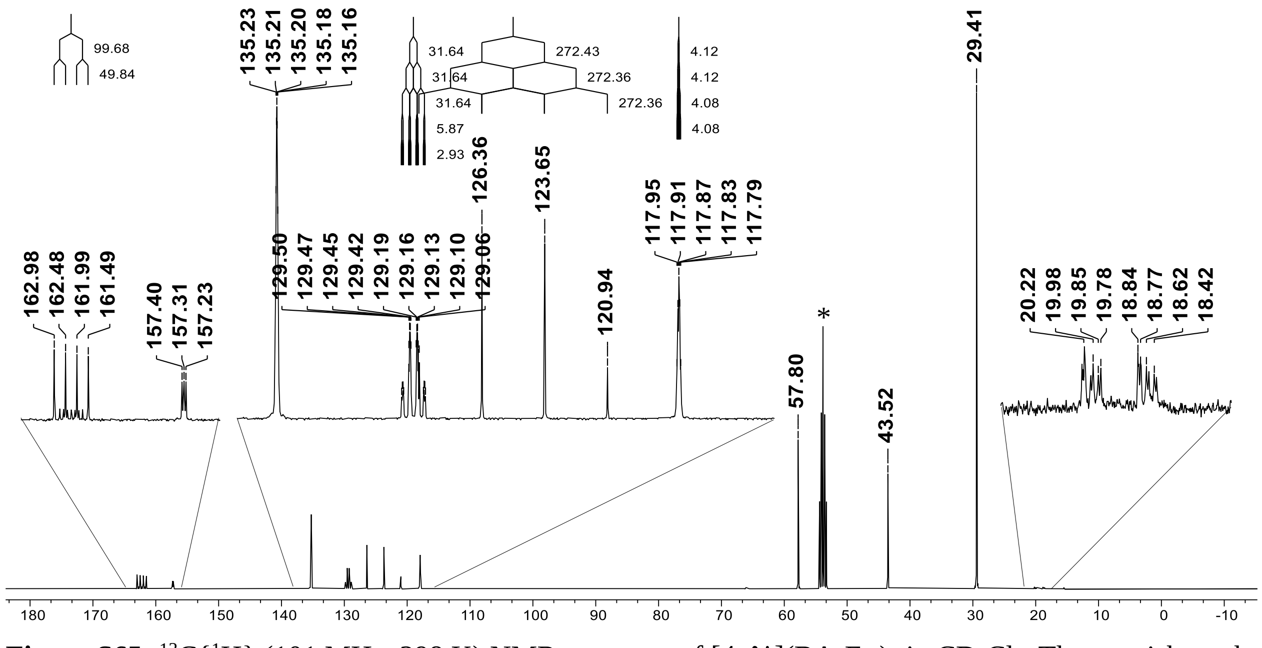

Figure S65: $^{13}C\{^1H\}$ (101 MHz, 298 K) NMR spectrum of [**4a'***]($BArF_{24}$)$_2$ in CD_2Cl_2. The asterisk marks the solvent signal.

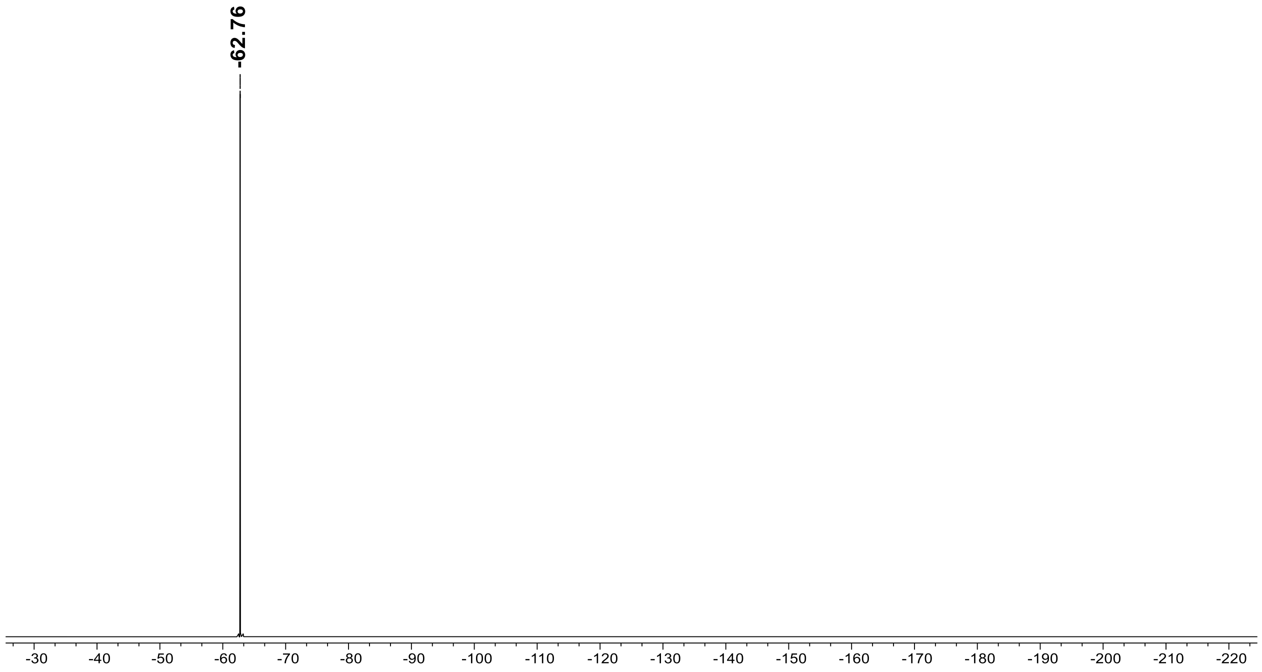

Figure S66: ^{19}F NMR (377 MHz, 300 K) spectrum of [**4a'***]($BArF_{24}$)$_2$ in CD_2Cl_2.

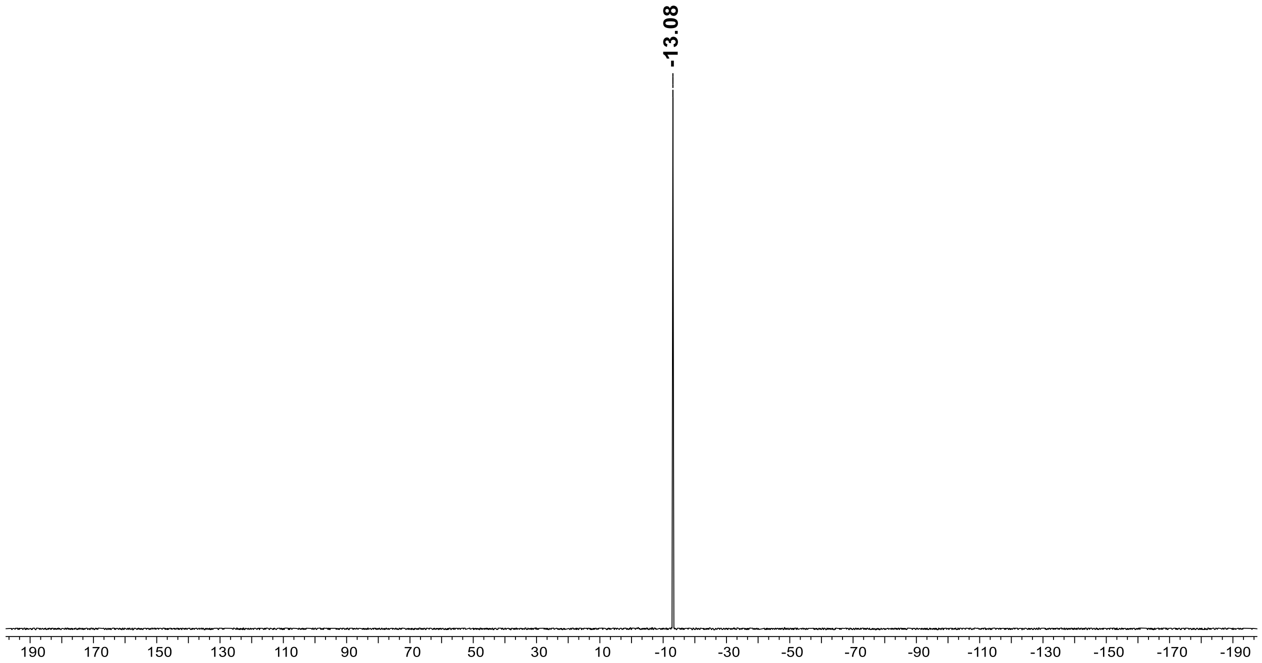

Figure S67: ^{31}P{^{1}H} NMR (192 MHz, 300 K) spectrum of [**4a'***]($BArF_{24}$)$_2$ in CD_2Cl_2.

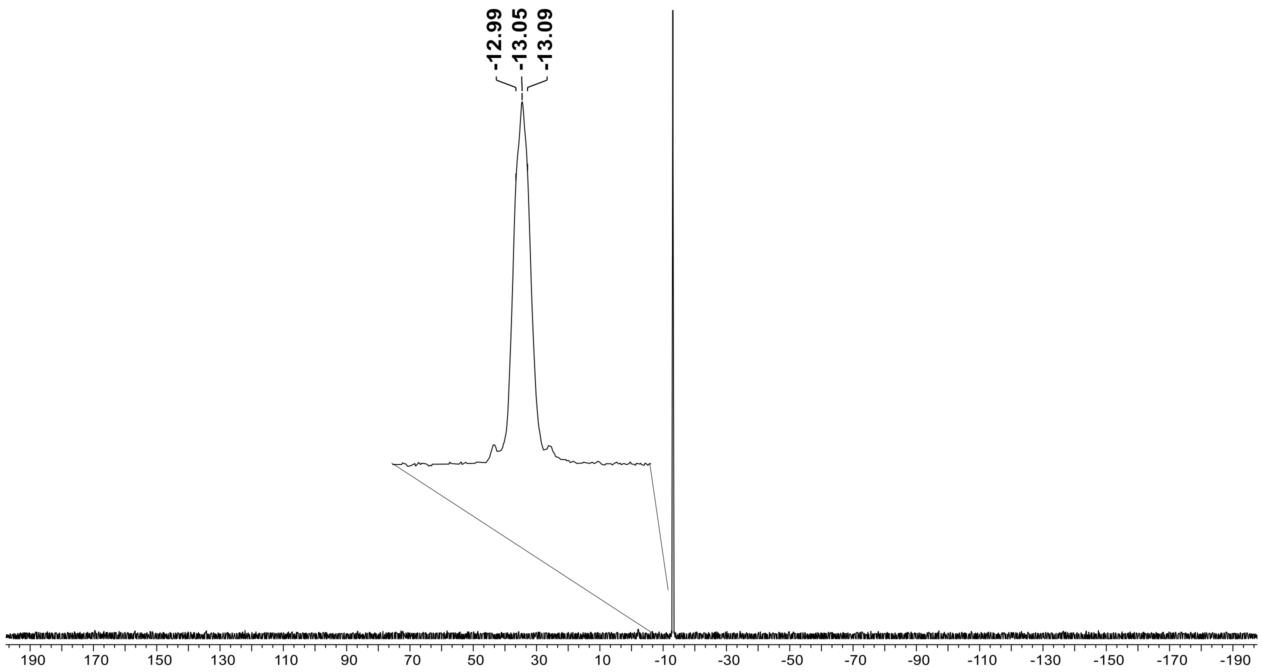

Figure S68: ^{31}P NMR (162 MHz, 300 K) spectrum of [**4a'***]($BArF_{24}$)$_2$ in CD_2Cl_2.

1.12 Crude ^{31}P NMR spectrum containing [4b]OTf

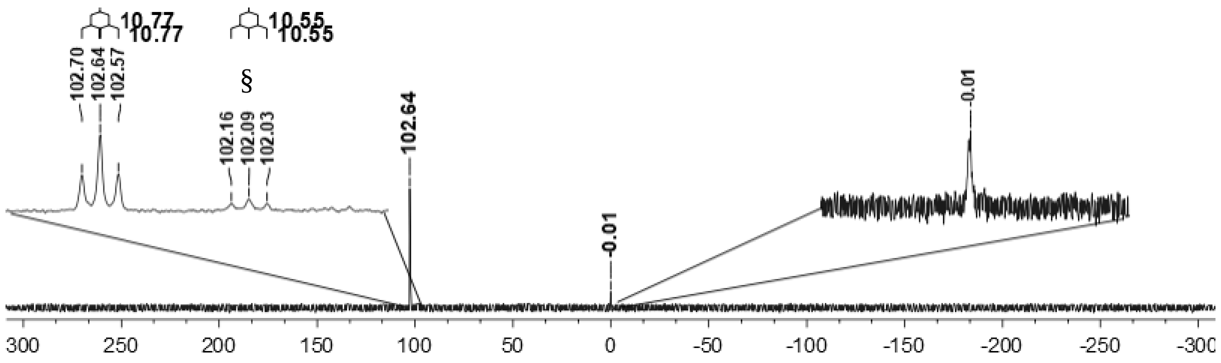

Figure S 69: ^{31}P NMR (162 MHz, 300 K) spectrum of the crude mixture containing [**4b**]OTf in C_6D_6/fluorobenzene. The signal marked with a section sign (§) can be assigned to structural isomers of [**4b**]OTf within the Dipp group due to isomeric impurities in the 2,6-diisopropylaniline employed for its synthesis. This observation was already reported in a previous study using the same starting materials.[2]

1.13 Characterization data of [7]BArF24

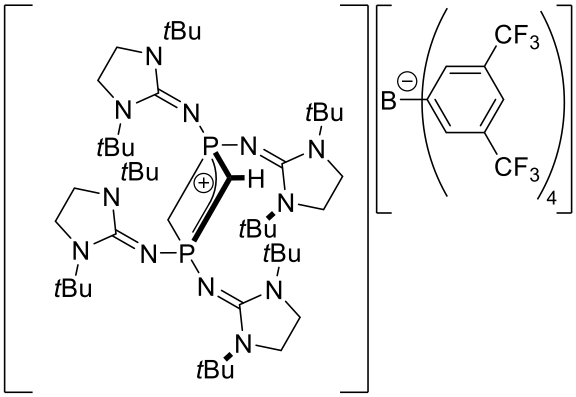

[**4a**]BArF24 (0.075 mmol, 98 mg, 1.0 eq.) was dissolved in tetrahydrofuran (2 mL) and filled into a Schlenk tube. The solution was cooled to -78°C. While stirring, KHMDS (0.075 mmol, 15 mg, 1.0 eq.) in tetrahydrofuran (2 mL) was added via a syringe. Immediately, the reaction was allowed to warm up to 21°C. The mixture was stirred at 21°C for 17 h. Afterwards, the volatiles were removed *in vacuo*. The product was not isolated. Mass of crude product: 73 mg.

Note: The NMR signals were assigned using 2D NMR experiments (*vide infra*).

^{1}H NMR (THF-*d*$_8$), 400 MHz, 300 K): δ (ppm) = 7.80 (m, 8 H, $BArF_{24}$; *ortho*), 7.58 (m, 4 H, $BArF_{24}$; *para*), 3.61 (t, $^{2}J_{HP}$ = 17.8 Hz, 2 H, P-CH_2-P), 3.30 (s, 16 H, N-CH_2-CH_2-N), 2.76 (s, 1 H, P-CH-P), 1.38 (s, 72 H, *t*Bu).

^{13}C{^{1}H} NMR (THF-*d*$_8$, 101 MHz, 300 K): δ (ppm) = 162.8 (q, $^{1}J_{CB}$ = 50 Hz, $BArF_{24}$; *ipso*), 147.6 (m, N-C-N), 135.6 ($BArF_{24}$; *ortho*), 130.1 (qq, $^{2}J_{CF}$ = 32 Hz $^{4}J_{CF}$ = 3 Hz, $BArF_{24}$; *meta*), 125.5 (q, $^{1}J_{CF}$ = 272 Hz, $BArF_{24}$; CF_3), 118.2 (sept, $^{3}J_{CB}$ = 4 Hz, $BArF_{24}$; *para*), 54.9 ($\underline{C}$-Me_3), 42.9 (N-CH_2-CH_2-N), 63.1 (t, $^{1}J_{CP}$ = 134 Hz, P-CH-P), 51.7 (t, $^{1}J_{CP}$ = 84 Hz, P-CH_2-P), 29.4 (C-$\underline{C}H_3$).

^{11}B NMR (THF-*d*$_8$, 160 MHz, 300 K): δ (ppm) = -6.5

^{19}F NMR (THF-*d*$_8$, 471 MHz, 300 K): δ (ppm) = -63.4.

^{31}P NMR (THF-*d*$_8$, 162 MHz, 300 K): δ (ppm) = -39.7 (t, $^{2}J_{PH}$ = 18 Hz).

^{31}P{^{1}H} NMR (THF-*d*$_8$, 162 MHz, 300 K): δ (ppm) = -39.7.

HR-MS(ESI): Calculated for $[C_{46}H_{91}N_{12}P_2]^+$ ([**7**]$^+$): m/z = 873.69594, found: m/z = 873.69443. Calculated for $[C_{46}H_{92}N_{12}P_2]^{2+}$ ([**7**+H]$^{2+}$): m/z = 437.35161, found: m/z = 437.35050 (Note: calculated m/z is identical to [**4a**]$^+$. z = 2 was confirmed by measurement).

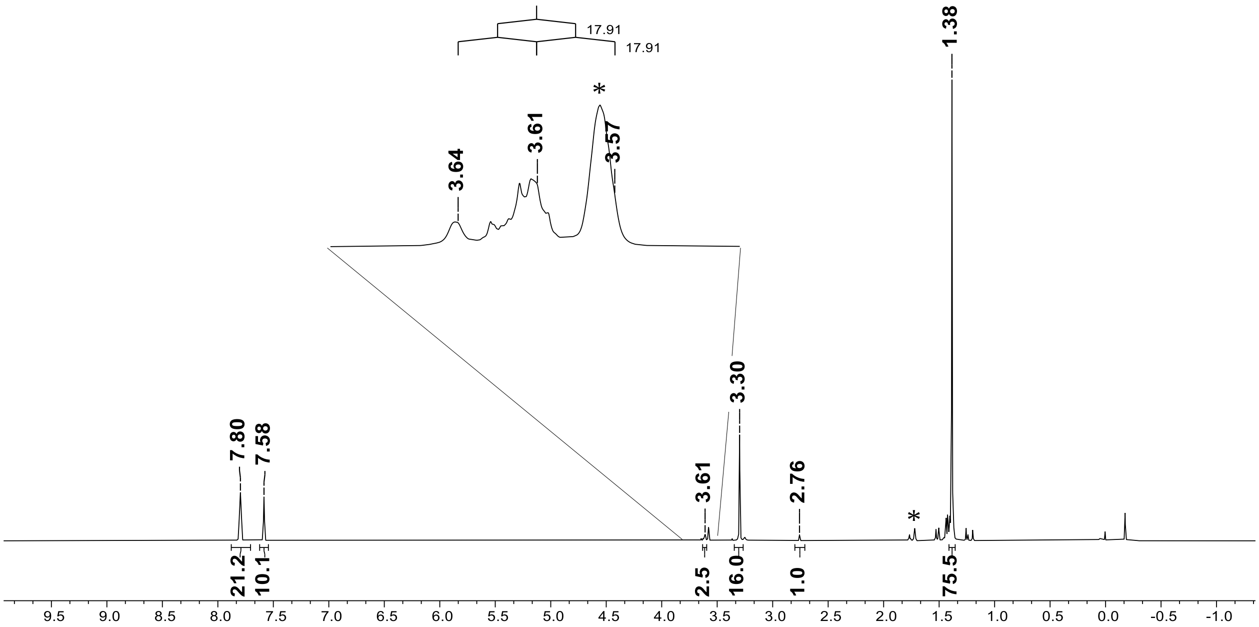

Figure S70: ^{1}H NMR (400 MHz, 300 K) spectrum of the crude mixture containing [**7**]BArF$_{24}$ in THF-d_8. The asterisks mark the solvent signals. Note: the triplet at 3.61 ppm is severely overlapped by impurities and the solvent.

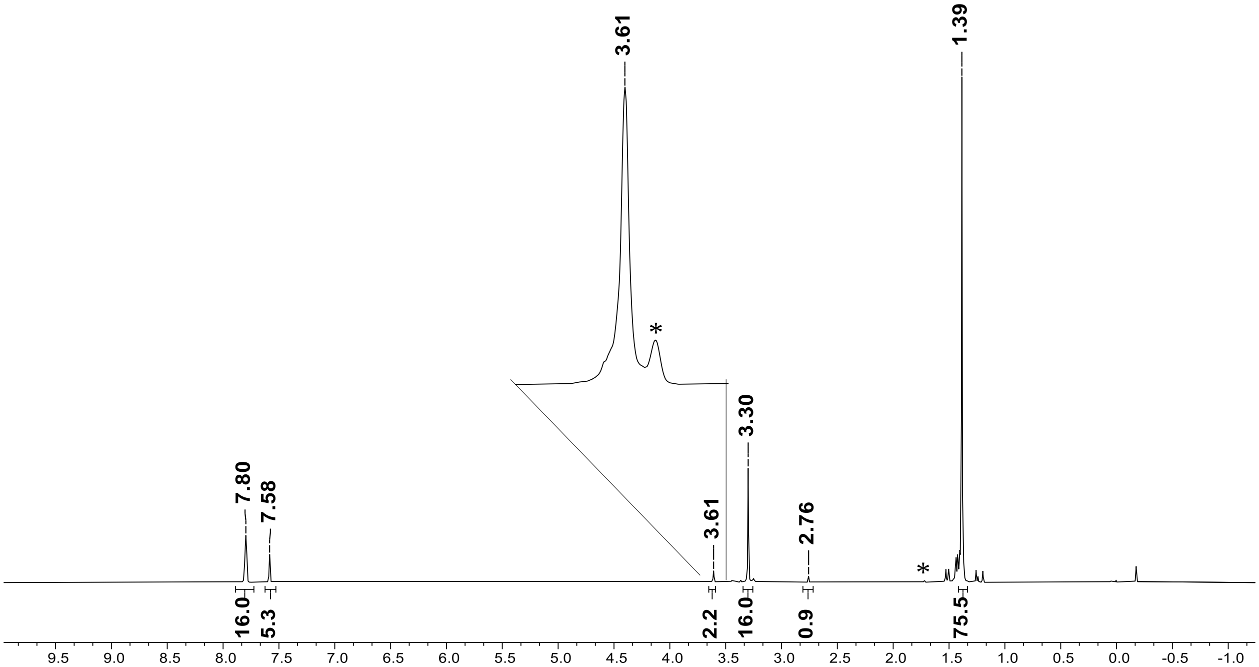

Figure S71: $^{1}H\{^{31}P\}$ NMR (400 MHz, 300 K) spectrum of the crude mixture containing [**7**]BArF$_{24}$ in THF-d_8. The asterisks mark the solvent signals (signal at 1.73 ppm too weak to be seen at the given amplification level).

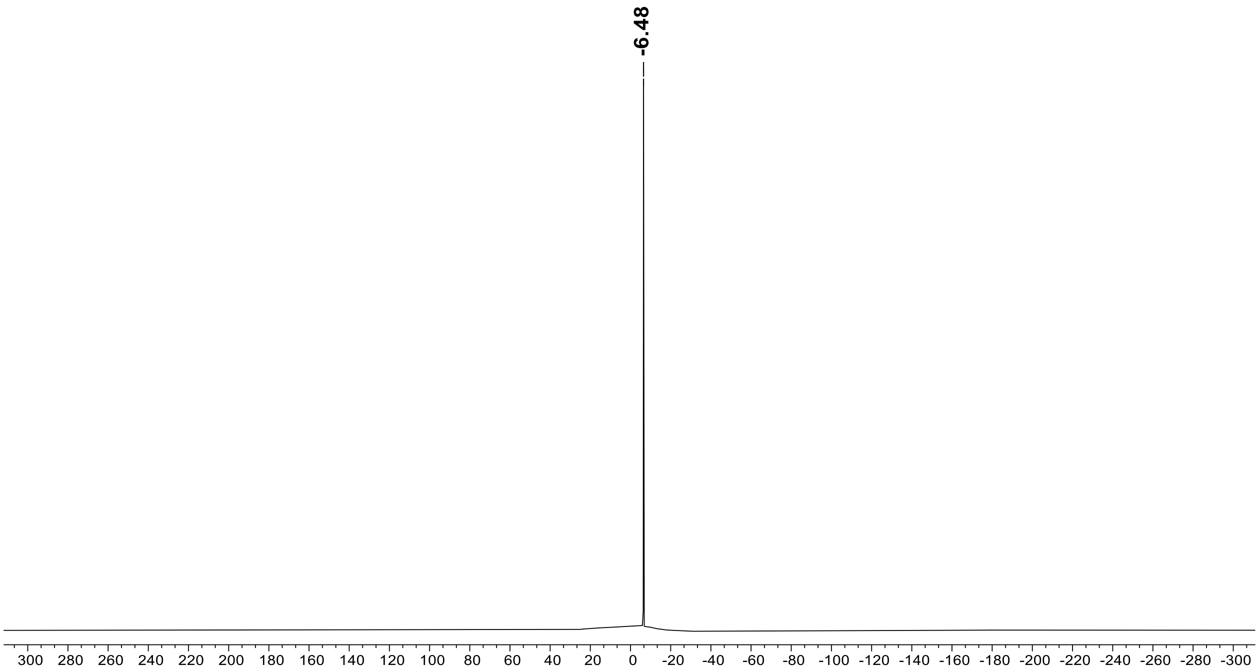

Figure S72: $^{11}B\{^1H\}$ NMR (160 MHz, 300 K) spectrum of the crude mixture containing [**7**]BArF$_{24}$ in THF-*d*$_8$.

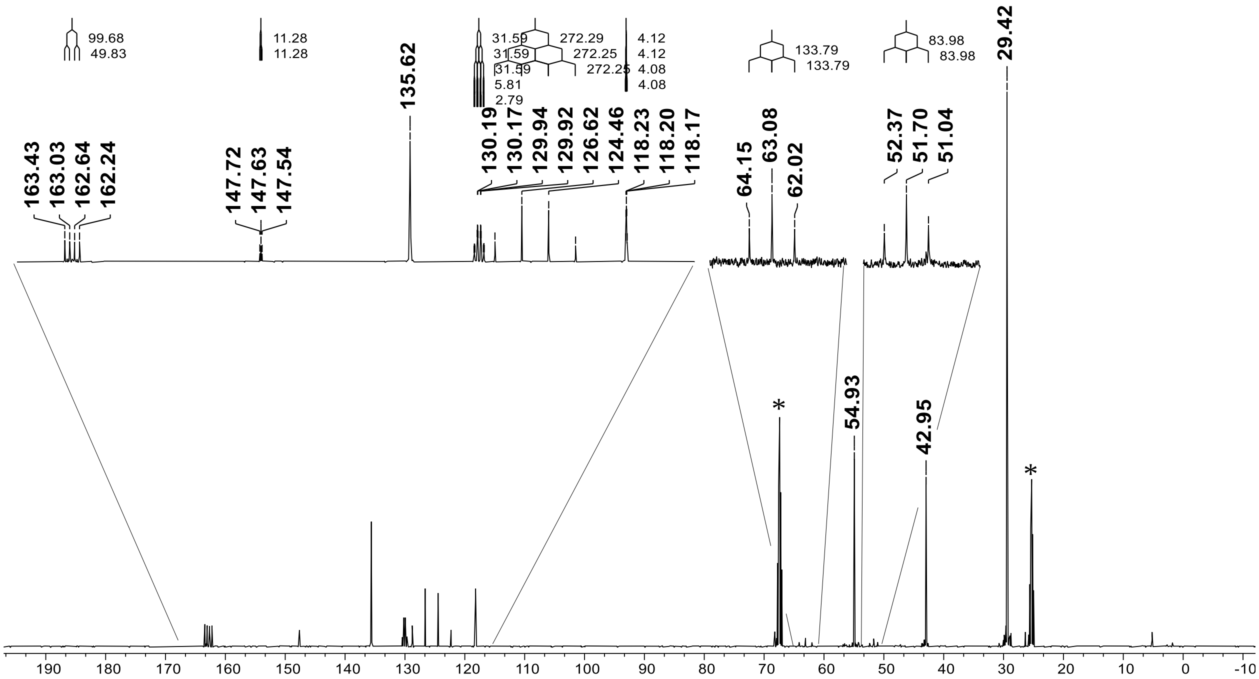

Figure S73: $^{13}C\{^1H\}$ NMR (101 MHz, 300 K) spectrum of the crude mixture containing [**7**]BArF$_{24}$ in THF-*d*$_8$. The asterisks mark the solvent signals.

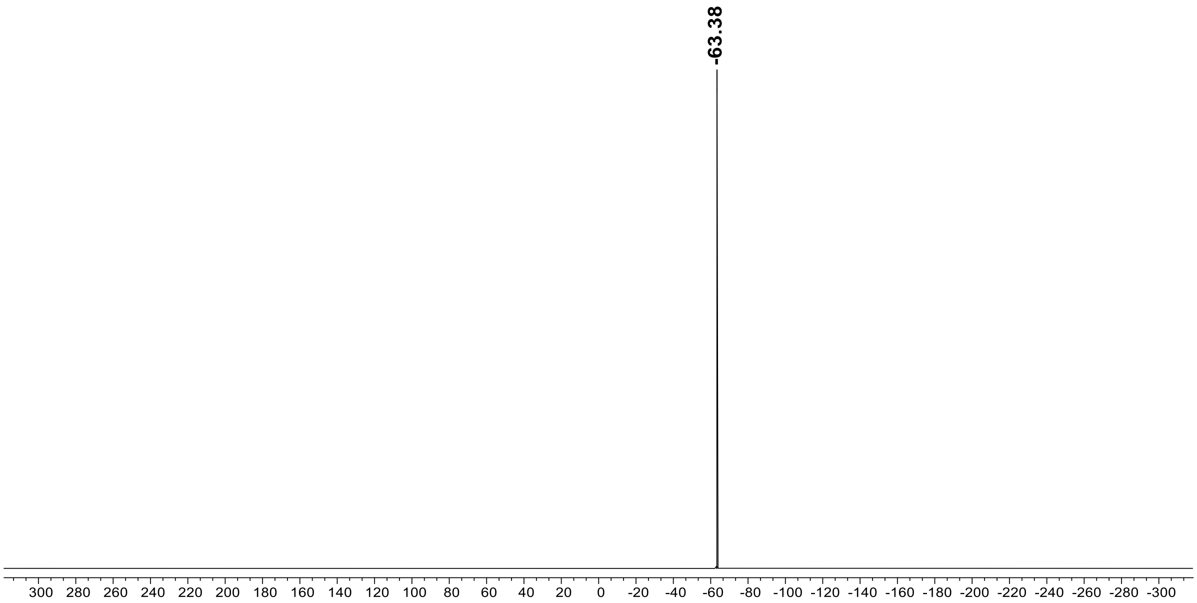

Figure S74: ^{19}F NMR (471 MHz, 300 K) spectrum of the crude mixture containing [**7**]BArF$_{24}$ in THF-d_8.

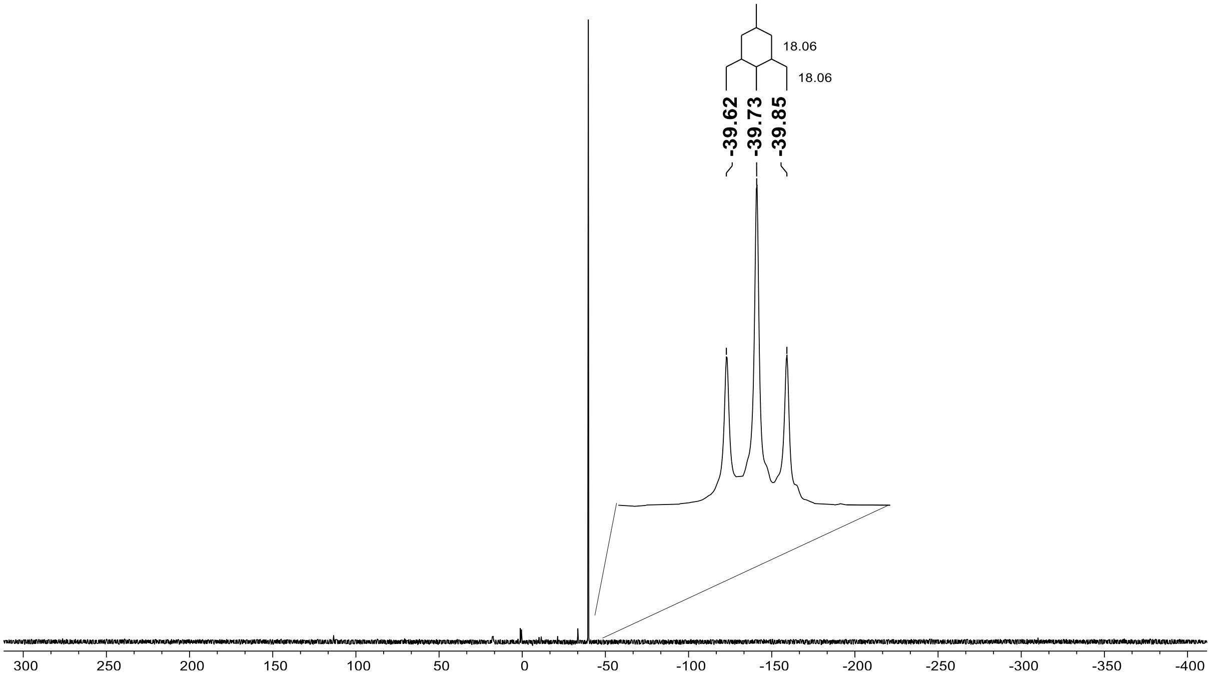

Figure S75: ^{31}P NMR (162 MHz, 300 K) spectrum of the crude mixture containing [**7**]BArF$_{24}$ in THF-d_8.

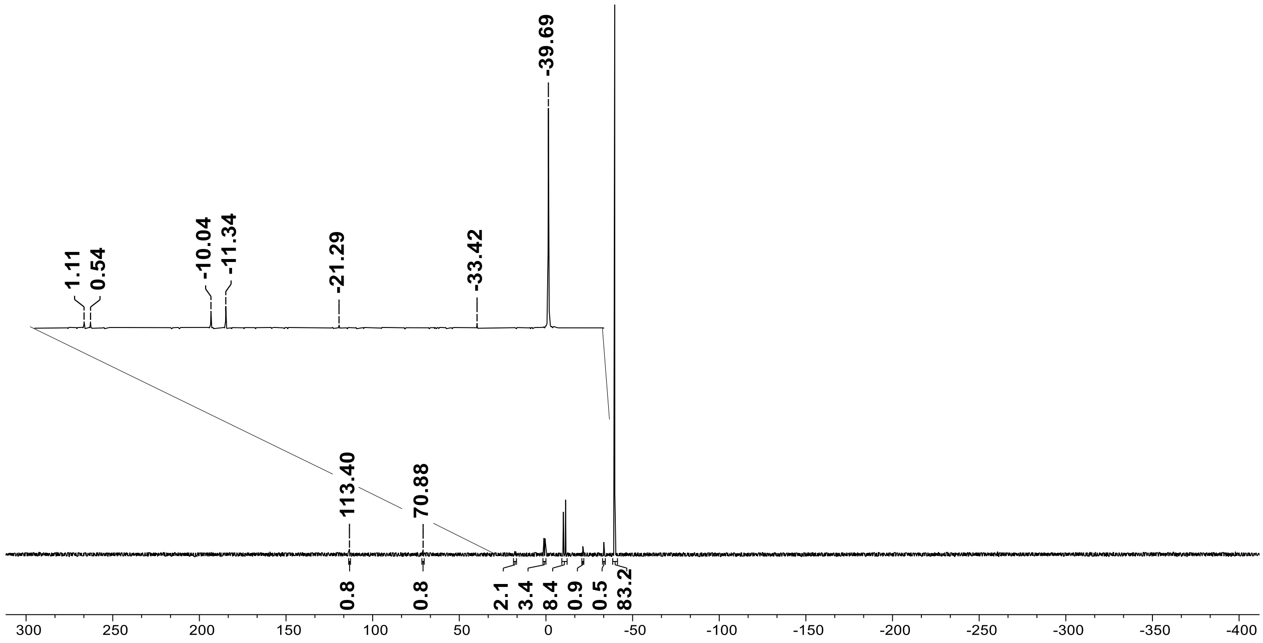

Figure S76: $^{31}P\{^{1}H\}$ NMR (162 MHz, 300 K) spectrum of the crude mixture containing [**7**]BArF$_{24}$ in THF-d_8.

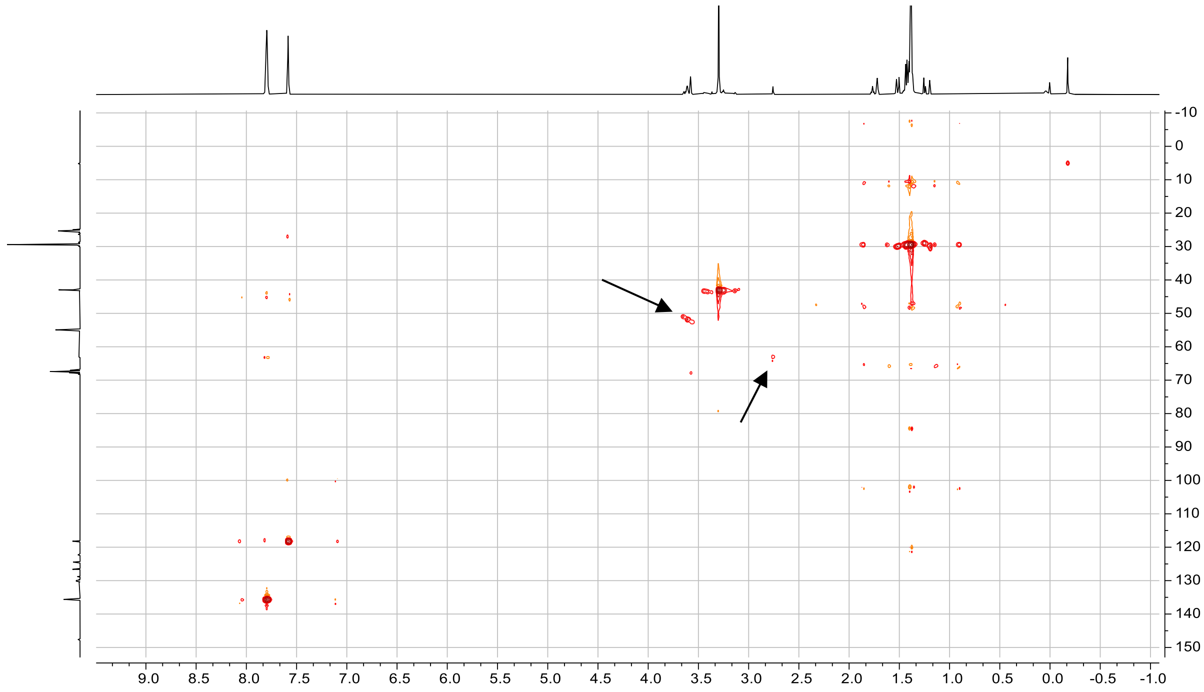

Figure S77: $^{1}H/^{13}C$ HSQC NMR spectrum of the crude mixture containing [**7**]BArF$_{24}$ in THF-d_8. The arrows mark the signals corresponding to the C_2P_2 heterocyclus.

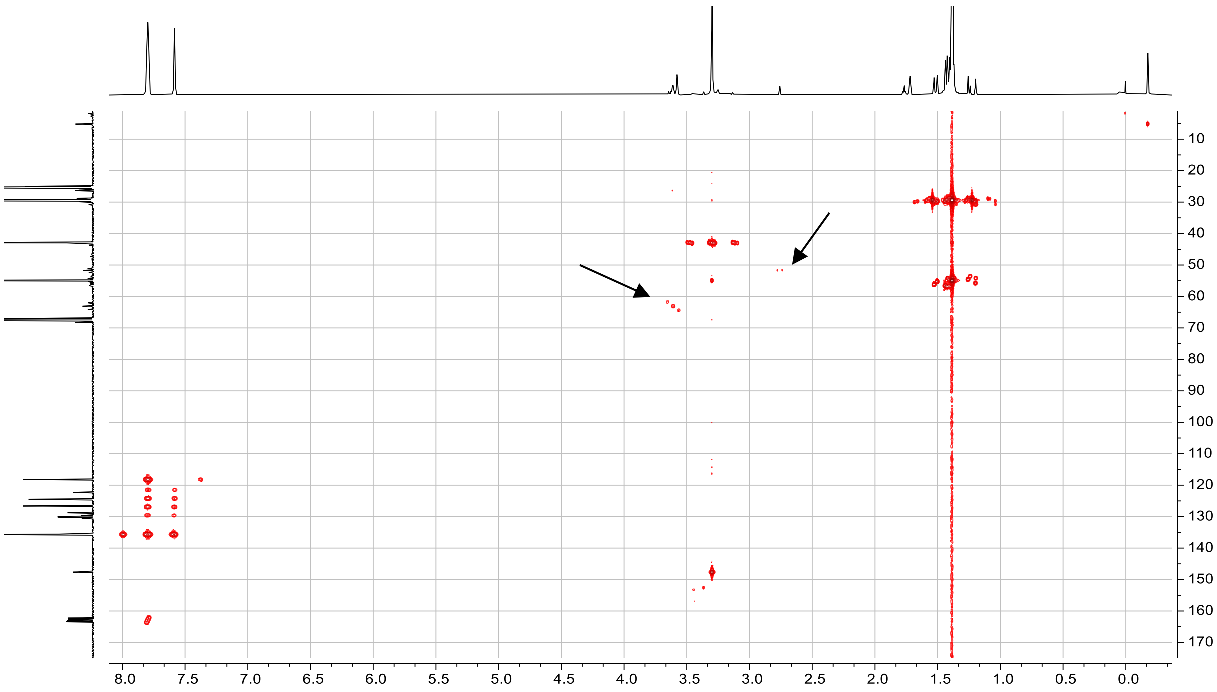

Figure S78: $^{1}H/^{13}C$ HMBC NMR spectrum of the crude mixture containing [**7**]$BArF_{24}$ in THF-d_8. The arrows mark the signals corresponding to the C_2P_2 heterocyclus.

1.14 Deprotonation study of [4a]BArF24

General procedure: [**4a**][BArF$_{24}$] was dissolved in the respective solvent. The solution was brought to the respective temperature. The base was quickly added and the reaction was allowed to warm up to 21°C. Afterwards, the reaction was analyzed via ^{31}P and ^{31}P{^{1}H} NMR. The results are summarized in Table S 1.

Table S 1: Results of the deprotonation study of [**4a**]BArF$_{24}$ with various bases. Eq.= equivalents of base. LiHMDS = lithium bis(trimethylsilyl)amide, LiHMDS = potassium bis(trimethylsilyl)amide, LDA = lithium diisopropylamide.

Base	Eq.	Temp.	Solvent	Detection of [**7**]BArF$_{24}$	Comments
n-butyllithium	1	-78 °C	Et$_2$O	Yes	Unselective; [**7**]BArF$_{24}$ is the major product
sec-butyllithium	1	-105 °C	Et$_2$O	Yes	Unselective; [**7**]BArF$_{24}$ is the major product
tert-butyllithium	1	-105 °C	Et$_2$O	No	Unselective; complex mixture
tert-butyllithium	1	-78 °C	*n*-hexane	Yes	Unselective; [**7**]BArF$_{24}$ is the major product
tert-butyllithium	1	-78 °C	THF	Yes	Unselective; [**7**]BArF$_{24}$ is the major product
LiHMDS	2	21 °C	Et$_2$O	Yes	Unselective; [**7**]BArF$_{24}$ is the major product
KHMDS	1	-78 °C	Et$_2$O	Yes	Unselective; [**7**]BArF$_{24}$ is the major product
KHMDS	1	-78 °C	THF	Yes	Unselective; [**7**]BArF$_{24}$ is the major product
KHMDS	4	-78 °C	Et$_2$O	Yes	Unselective; [**7**]BArF$_{24}$ is the major product
KHMDS	1	-78 °C	Et$_2$O	Yes	Inverse addition order ([**4a**]BArF$_{24}$ to KHMDS). Unselective; [**7**]BArF$_{24}$ is the major product
KO*t*Bu	4	-78 °C	Et$_2$O	Yes	Unselective; [**7**]BArF$_{24}$ is the major product
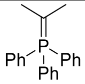	4	-78 °C	Et$_2$O	Yes	Unselective; complex mixture
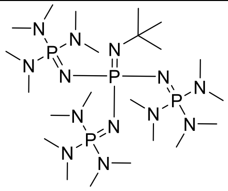	4	-78 °C	Et$_2$O	Yes	Unselective; [**7**]BArF$_{24}$ is the major product
LDA	4	-78 °C	Et$_2$O	No	Unselective; complex mixture

1.15 Preparation of 8

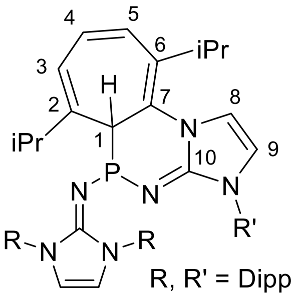

To a mixture of [**3b**]OTf (0.08 mmol, 92 mg, 1.0 eq.) and KO*t*Bu (0.36 mmol, 40 mg, 4.5 eq.), diethyl ether (3 mL) was added at 21 °C and the suspension was stirred for 16 h. Subsequently, all volatiles were removed *in vacuo*. The residue was extracted with *n*-hexane (2x5 mL) and the obtained yellow solution was concentrated until crystal formation. The mixture was heated again until dissolution of the crystals, then slowly cooled to 21 °C and then stored at -40 °C. The product was isolated as yellow crystals.

Note: The NMR signals were assigned using 2D NMR experiments (*vide infra*).

Yield: 21% (0.017 mmol, 15 mg).

^{1}H NMR (C_6D_6, 400 MHz, 300 K): δ (ppm) = 7.28 (t, $^{3}J_{HH}$ = 7.3 Hz, 1 H, CH R´; *para*), 7.22 (t, $^{3}J_{HH}$ = 7.1 Hz, 2 H, CH R; *para*), 7.16 - 7.17 (m, 2 H, CH R´; *meta*), 7.12 (d, $^{3}J_{HH}$ = 7.1 Hz, 2 H, CH R; *meta*), 7.08 (d, $^{3}J_{HH}$ = 7.1 Hz, 2 H, CH R; *meta*), 6.71 (dd, $^{3}J_{HH}$ = 10.7 Hz, $^{3}J_{HH}$ = 6.1 Hz, 1 H, CH 4), 6.55 (d, $^{3}J_{HH}$ = 10.7 Hz, 1 H, CH 5), 6.25 (d, $^{3}J_{HH}$ = 6.1 Hz, 1 H, CH 3), 6.24 (d, $^{3}J_{HH}$ = 3 Hz, 1 H, CH 8), 5.85 (s, 2 H, CH N-CH=CH-N), 5.68 (d, $^{3}J_{HH}$ = 3 Hz, 1 H, CH 9), 3.34 (m, 1 H, CH *i*Pr; 2), 3.26 (m, 1 H, CH *i*Pr; 6), 3.14 (m, 2 H, CH R; *i*Pr), 3.06 (m, 2 H, CH R; *i*Pr), 3.10 (m, 1 H, CH R´; *i*Pr) 2.80 (m, 1 H, CH R´; *i*Pr), 1.36 (d, $^{3}J_{HH}$ = 7.1 Hz, 12 H, CH_3 R; *i*Pr), 1.20 (d, $^{3}J_{HH}$ = 7.1 Hz, 6 H, CH_3 R; *i*Pr), 1.14 (m, 3 H, CH_3 R´; *i*Pr), 1.11 (m, 3 H, CH_3 R´; *i*Pr), 1.03 (m, 6 H, CH_3 R; *i*Pr), 1.03 (d, $^{2}J_{HP}$ = 11 Hz, 1 H, CH 1), 1.01 (m, 3 H, CH_3 R´; *i*Pr), 1.01 (m, 6 H, CH_3 *i*Pr; 6), 0.67 (d, $^{3}J_{HH}$ = 6.8 Hz, 3 H, CH_3 *i*Pr; 2), 0.56 (d, $^{3}J_{HH}$ = 6.8 Hz, 3 H, CH_3 *i*Pr; 2).

^{13}C NMR (C_6D_6, 101 MHz, 300 K): δ (ppm) = 151.1 (d, $^{2}J_{CP}$ = 32 Hz, C_q C=N-P), 149.2 (d, $^{2}J_{CP}$ = 2 Hz, C_q 2), 148.9 (C_q R´; *ortho*), 148.4 (C_q R´; *ortho*), 147.7 (d, $^{5}J_{CP}$ = 1 Hz, C_q R; *ortho*), 147.2 (d, $^{5}J_{CP}$ = 2 Hz, C_q R; *ortho*), 142.3 (d, $^{2}J_{CP}$ = 5 Hz, C_q C=N-P; 10), 135.2 (d, $^{4}J_{CP}$ = 1 Hz, C_q R; *ipso*), 134.1 (C_q R´; *ipso*), 129.4 (CH R; *para*), 129.2 (CH R´; *para*), 127.6 (d, $^{4}J_{CP}$ = 1 Hz, CH 4), 124.4 (CH 5), 124.3 (CH R; *meta*), 123.9 (CH R´; *meta*), 123.8 (d, $^{2}J_{CP}$ = 5 Hz, C_q 7), 123.7 (CH R/R´; *meta*), 120.4 (CH 3), 115.6 (CH N-CH=CH-N), 115.0 (CH 9), 112.8 (CH 8), 112.3 (d, $^{3}J_{CP}$ = 8 Hz, C_q 6), 42.1 (d, $^{1}J_{CP}$ = 28 Hz, CH 1), 30.2 (CH *i*Pr; 2) 29.3 (CH *i*Pr; R), 29.0 (CH *i*Pr; R), 28.7 (CH *i*Pr; R´), 28.7 (CH *i*Pr; 6), 28.4 (CH *i*Pr; R´), 26.1 (CH_3 *i*Pr; 2), 26.1 (CH_3 *i*Pr; 2), 24.9 (CH_3 *i*Pr; R´), 24.8 (CH_3 *i*Pr; R), 24.7 (CH_3 *i*Pr; R´), 24.5 (CH_3 *i*Pr; 6), 24.3 (CH_3 *i*Pr; R´), 24.2 (CH_3 *i*Pr; R´), 24.2 (CH_3 *i*Pr; R), 23.4 (CH_3 *i*Pr; R), 23.4 (CH_3 *i*Pr; R), 22.8 (CH_3 *i*Pr; 6), 22.4 (CH_3 *i*Pr; R), 21.6 (CH_3 *i*Pr; 2), 21.6 (CH_3 *i*Pr; 2).

^{31}P NMR (C_6D_6, 162 MHz, 300 K): δ (ppm) = 73.2 (d, $^{2}J_{PH}$ = 11 Hz).

HR-MS (ESI): Calculated for $[C_{55}H_{74}N_6P]^+$ ([**8**+H]$^+$): m/z = 849.57071, found: m/z = 849.57132.

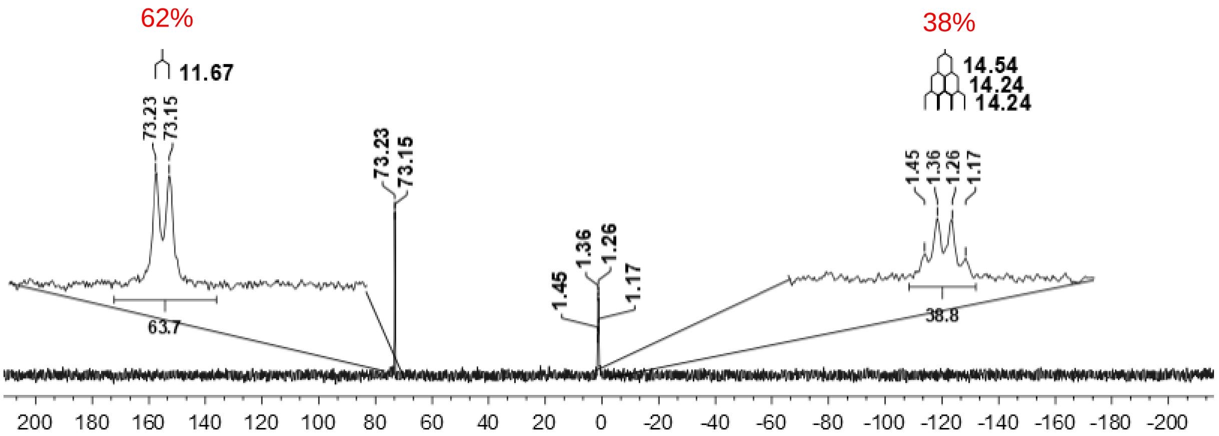

Figure S79: ^{31}P NMR (162 MHz, 300 K) spectrum of the crude reaction mixture containing **8** in C_6D_6/fluorobenzene. The NMR spectrum was acquired before the extraction step. NMR-yields of the corresponding species are shown in red.

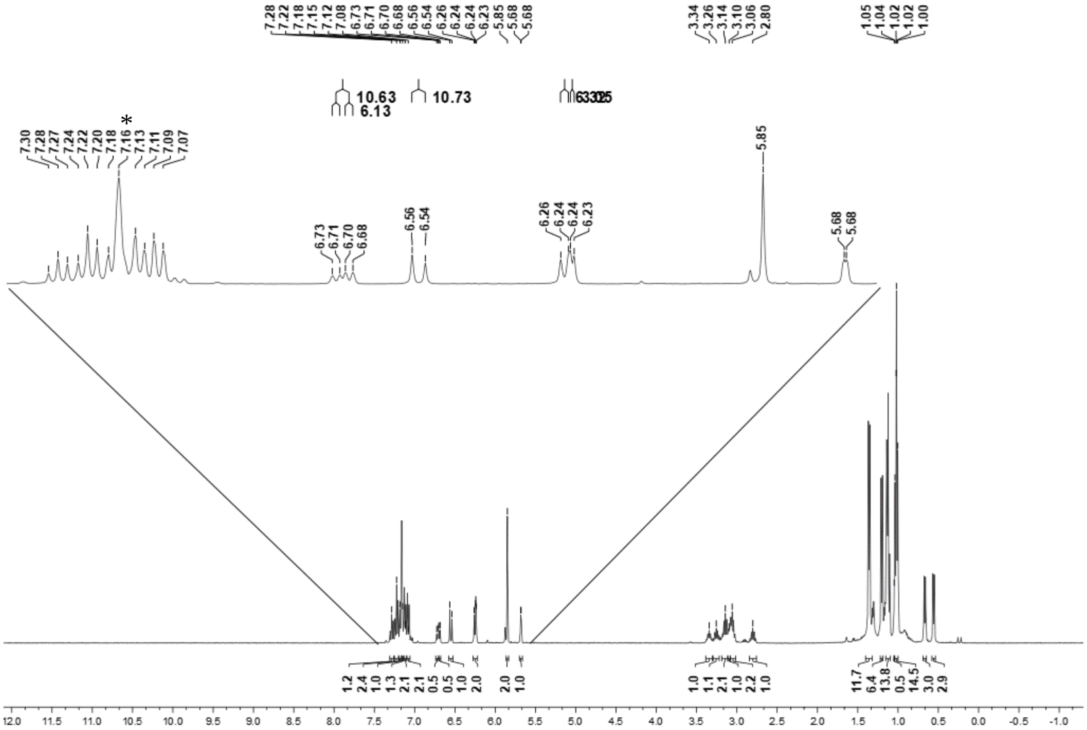

Figure S80: ^1H NMR (400 MHz, 300 K) spectrum of **8** in C_6D_6. The asterisk marks the solvent signal.

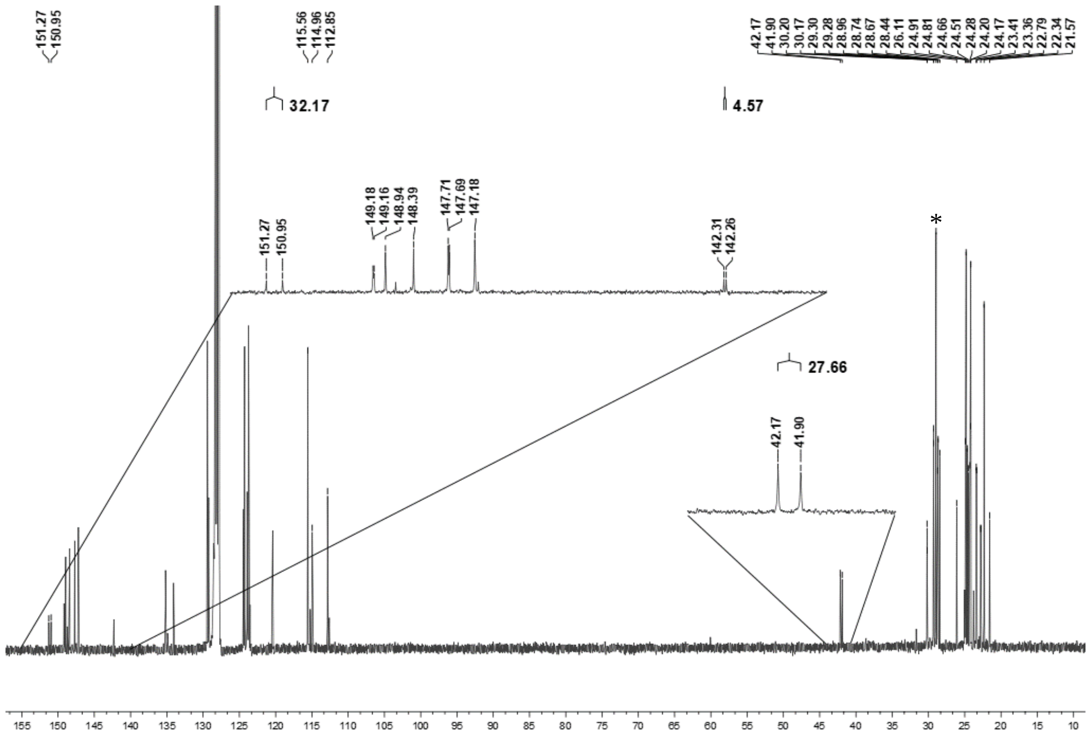

Figure S81: $^{13}C\{^{1}H\}$ NMR (101 MHz, 300 K) spectrum of **8** in C_6D_6. The asterisk marks the solvent signal.

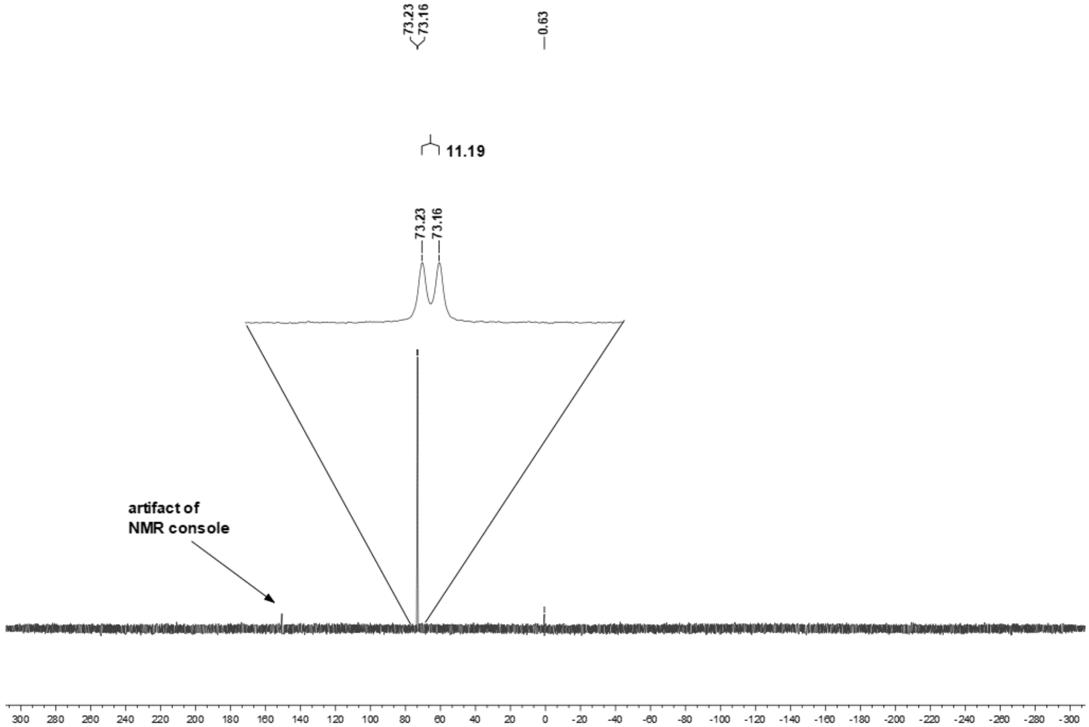

Figure S82: ^{31}P NMR (162 MHz, 300 K) spectrum of **8** in C_6D_6.

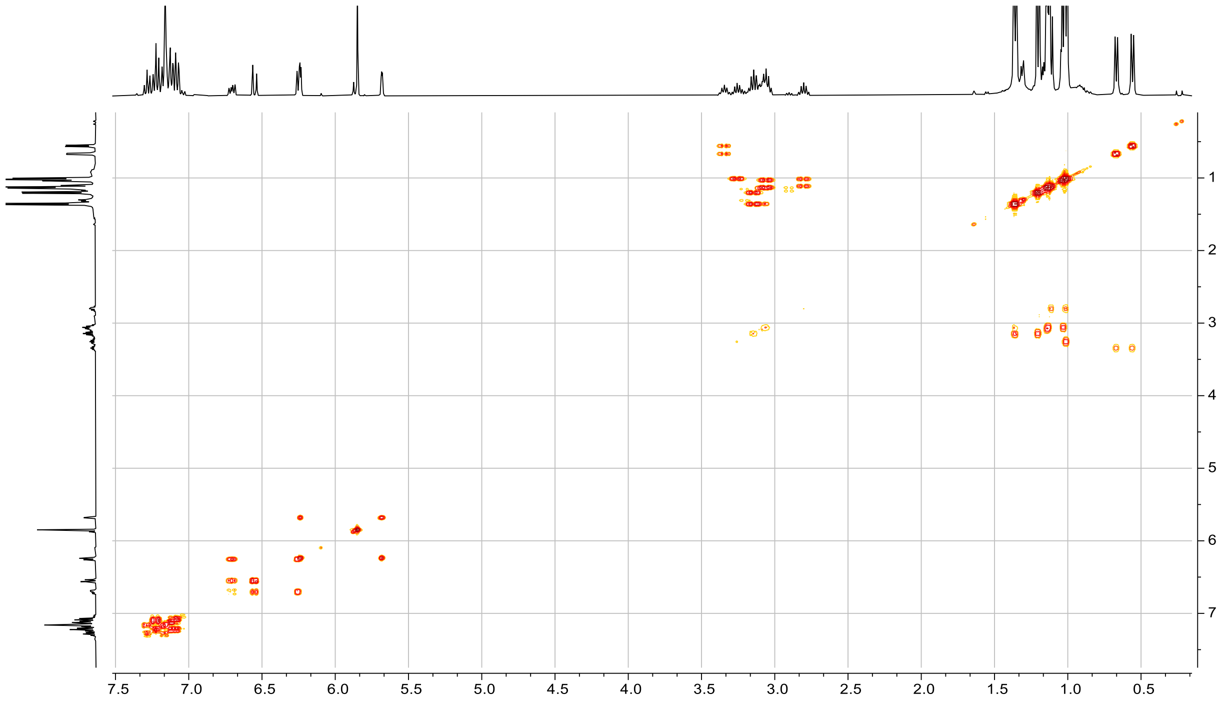

Figure S83: $^1H/^1H$ COSY NMR spectrum of **8** in C_6D_6.

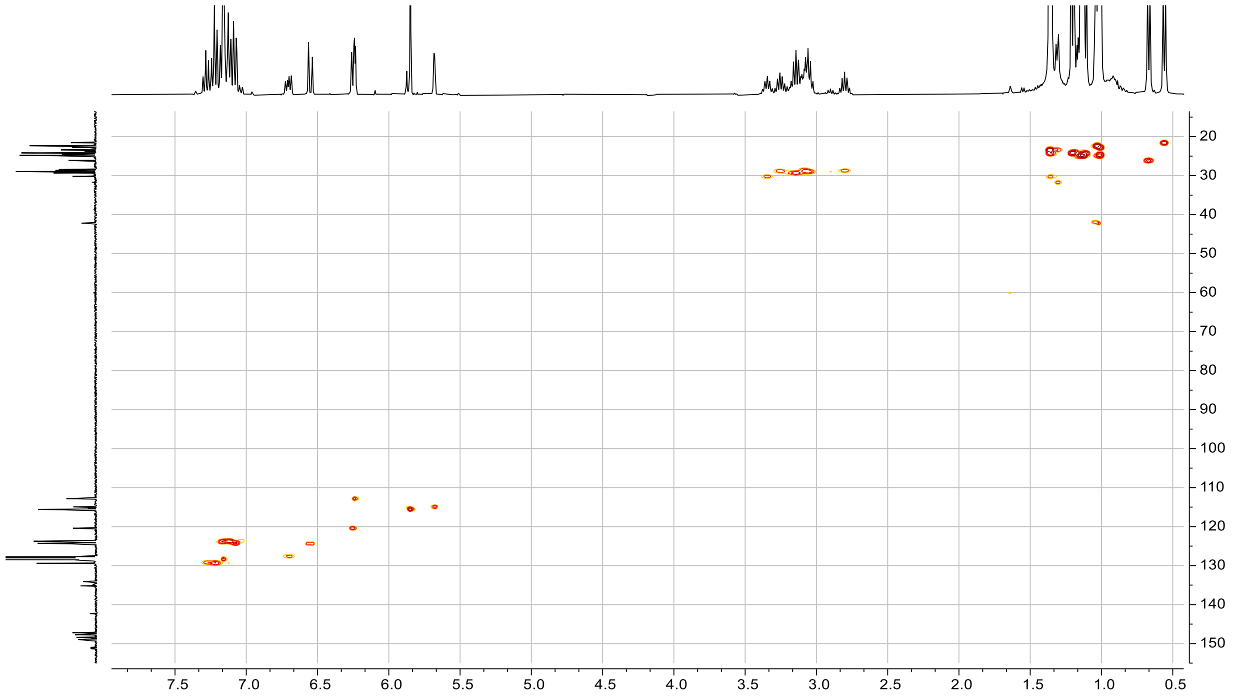

Figure S84: $^1H/^{13}C$ HSQC NMR spectrum of **8** in C_6D_6.

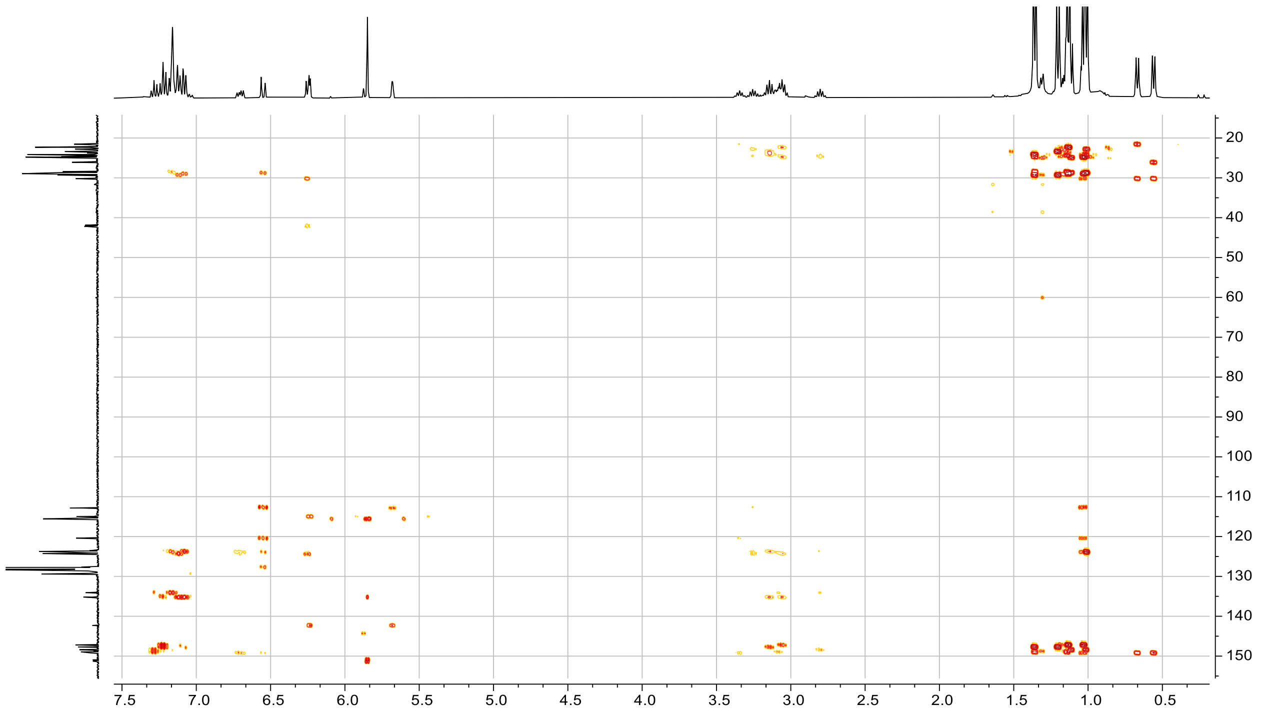

Figure S85: $^{1}H/^{13}C$ HMBC NMR spectrum of **8** in C_6D_6.

2 X-ray Diffraction Studies

General: Single-crystal X-ray diffraction data were collected on a Bruker AXS detector using Mo-K_α radiation (λ = 0.71073 Å). Crystals were selected under oil, mounted on glass capillaries and then immediately placed in a cold stream of N_2 on a diffractometer. Using Olex2,[3] the structures were solved with the Superflip[4] structure solution program using Charge Flipping, SIR2004[5] using direct methods or ShelXT[6] using Intrinsic Phasing and refined with the ShelXL[7] refinement package using Least Squares minimisation.[8]

Crystallographic data have been deposited with the Cambridge Crystallographic Data Centre as supplementary publication no. 2244191-2244199. These data can be obtained free of charge via www.ccdc.cam.uk/data_request/cif (or from the CCDC, 12 Union Road, Cambridge CB2 1EZ, UK; fax: (+44) 1223-336-033; or deposit@ccdc.cam.ac.uk).

2.1 Crystal structure data of compound 2a

CCDC deposition number	**2244198**	ρ_{calc}g/cm^3	1.136
Empirical formula	$C_{23}H_{47}N_6P$	μ/mm^{-1}	0.128
Formula weight	438.63	F(000)	968.0
Temperature/K	100	Crystal size/mm^3	0.742 × 0.589 × 0.442
Crystal system	monoclinic	Radiation	MoKα (λ = 0.71073)
Space group	*P*2$_1$/*c*	2Θ range for data collection/°	4.056 to 59.148
a/Å	12.9856(3)	Index ranges	-18 ≤ h ≤ 18, -13 ≤ k ≤ 13, -28 ≤ l ≤ 28
b/Å	9.8313(2)	Reflections collected	41012
c/Å	20.5049(5)	Independent reflections	7191 [R_{int} = 0.0326, R_{sigma} = 0.0194]
α/°	90	Data/restraints/parameters	7191/0/294
β/°	101.6430(10)	Goodness-of-fit on F^2	1.157
γ/°	90	Final R indexes [I>=2σ (I)]	R_1 = 0.0388, wR_2 = 0.1018
Volume/Å^3	2563.90(10)	Final R indexes [all data]	R_1 = 0.0399, wR_2 = 0.1026
Z	4	Largest diff. peak/hole / e Å^{-3}	0.48/-0.38

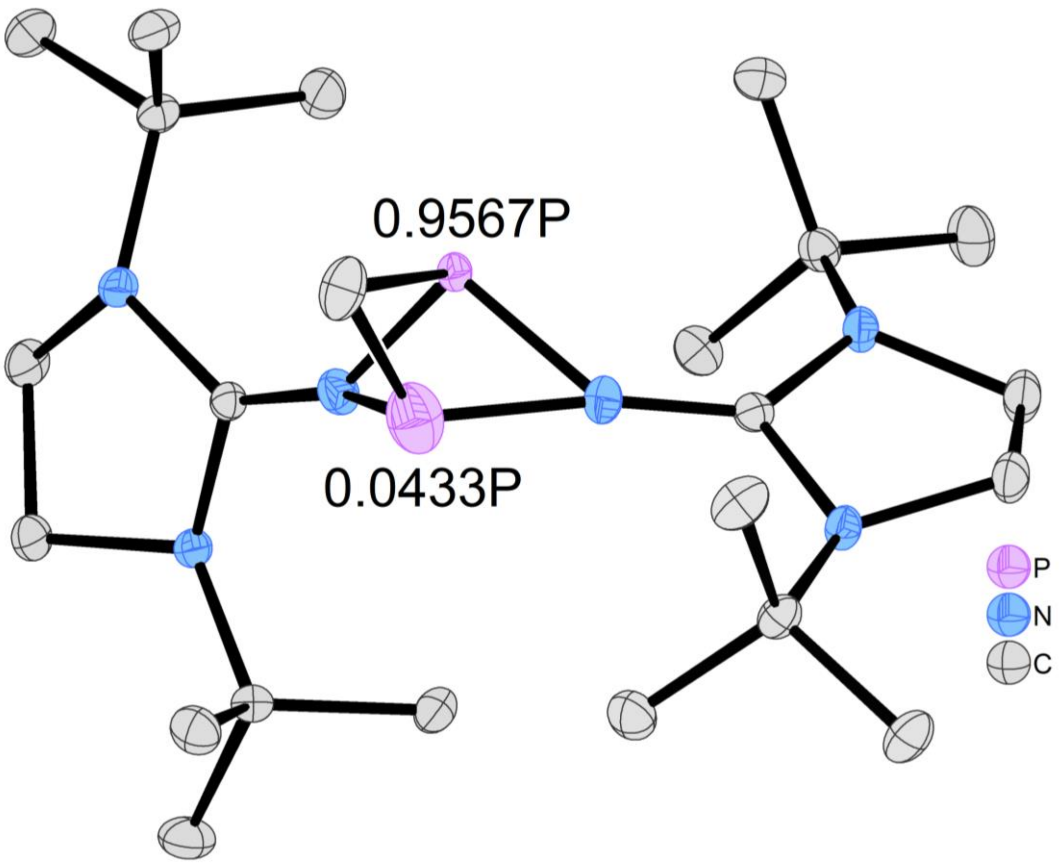

Figure S86: The asymmetric unit contains one molecule of **2a**. The phosphorus atom is disordered over two positions (see image).

2.2 Crystal structure data of compound 2b

CCDC deposition number	**2244195**
Empirical formula	$C_{55}H_{75}N_6P$
Formula weight	851.18
Temperature/K	100
Crystal system	orthorhombic
Space group	*Pca*2_1
a/Å	27.9781(6)
b/Å	12.6688(3)
c/Å	28.6912(6)
α/°	90
β/°	90
γ/°	90
Volume/Å^3	10169.6(4)
Z	8

ρ_{calc}mg/mm^3	1.112
μ/mm^{-1}	0.095
F(000)	3696.0
Crystal size/mm^3	0.43 × 0.16 × 0.15
Radiation	MoKα (λ = 0.71073)
2Θ range for data collection	2.912 to 51.39°
Index ranges	$-33 \le h \le 34$, $-15 \le k \le 15$, $-34 \le l \le 34$
Reflections collected	92278
Independent reflections	19296 [R_{int} = 0.0487, R_{sigma} = 0.0386]
Data/restraints/parameters	19296/38/1252
Goodness-of-fit on F^2	1.085
Final R indexes [I>=2σ (I)]	R_1 = 0.0641, wR_2 = 0.1602
Final R indexes [all data]	R_1 = 0.0748, wR_2 = 0.1679
Largest diff. peak/hole / e Å^{-3}	0.81/-0.37

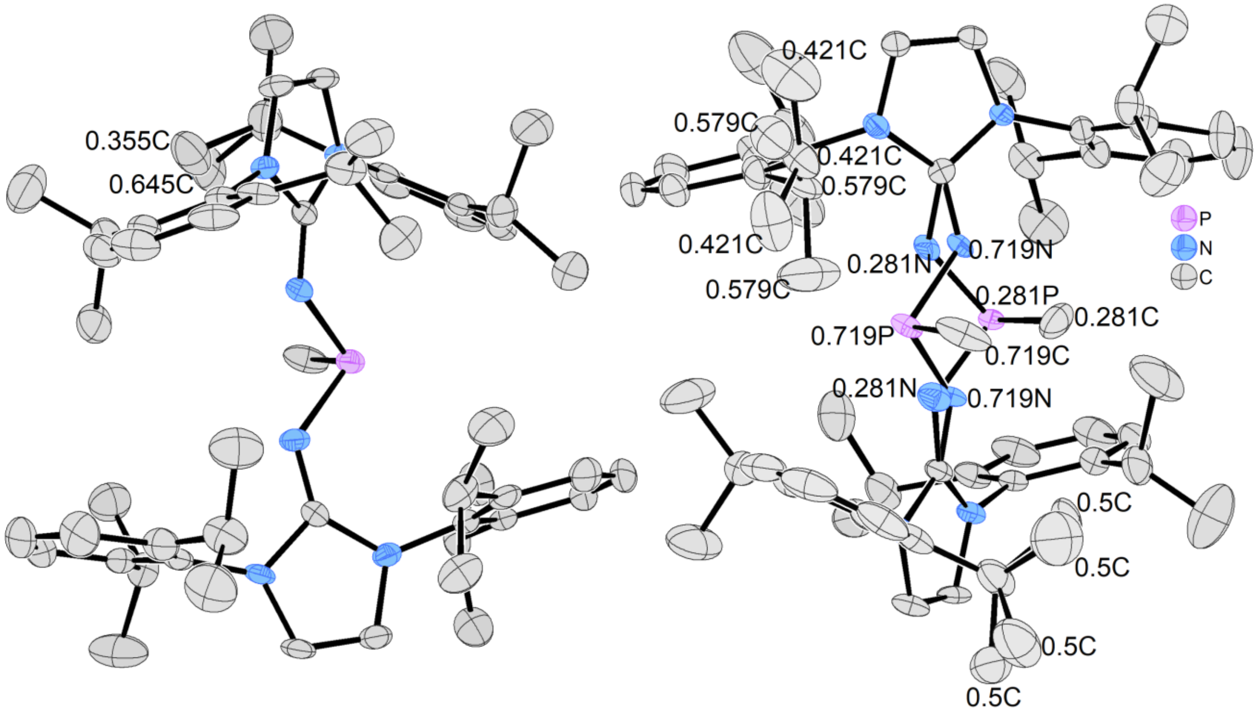

Figure S87: The structure is an inversion twin. The asymmetric unit contains two molecules of **2b**. One N_2PC fragment, two isopropyl groups and one methyl group are disordered over two positions (see atom labels).

2.3 Crystal structure data of compound [3a]I

CCDC deposition number	**2244192**
Empirical formula	$C_{23}H_{47}I_2N_6P$
Formula weight	692.43
Temperature/K	100
Crystal system	triclinic
Space group	$P\bar{1}$
a/Å	9.5526(3)
b/Å	12.6149(5)
c/Å	14.1053(5)
α/°	106.682(2)
β/°	106.508(2)
γ/°	103.779(2)
Volume/Å^3	1463.44(9)
Z	2

ρ_{calc}g/cm^3	1.571
μ/mm^{-1}	2.225
F(000)	696.0
Crystal size/mm^3	0.574 × 0.092 × 0.061
Radiation	MoKα (λ = 0.71073)
2Θ range for data collection/°	3.258 to 60.65
Index ranges	$-13 \leq h \leq 13$, $-17 \leq k \leq 17$, $-19 \leq l \leq 19$
Reflections collected	26020
Independent reflections	8672 [R_{int} = 0.0342, R_{sigma} = 0.0346]
Data/restraints/parameters	8672/0/311
Goodness-of-fit on F^2	1.086
Final R indexes [I>=2σ (I)]	R_1 = 0.0330, wR_2 = 0.0843
Final R indexes [all data]	R_1 = 0.0373, wR_2 = 0.0873
Largest diff. peak/hole / e Å^{-3}	1.95/-0.57

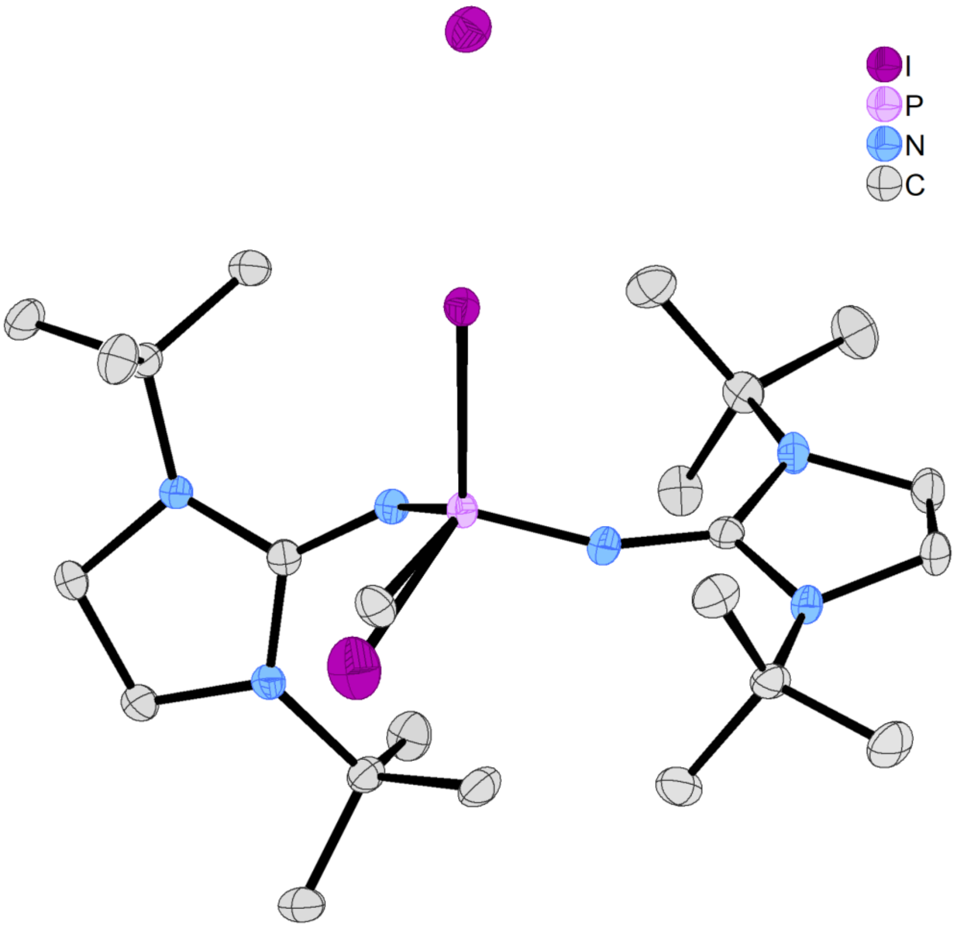

Figure S88: The asymmetric unit contains one molecule of [**3a**]I. The phosphorus bound methyl and iodine are disordered over two positions in a ratio of 24:1. The lesser populated methyl disorder (4%) could not be refined.

2.4 Crystal structure data of compound [3a]I_3

CCDC deposition number	**2244196**
Empirical formula	$C_{23}H_{47}I_4N_6P$
Formula weight	946.23
Temperature/K	100
Crystal system	monoclinic
Space group	$P2_1/c$
a/Å	19.1275(9)
b/Å	9.9593(5)
c/Å	17.3077(8)
α/°	90
β/°	92.711(3)
γ/°	90
Volume/Å^3	3293.4(3)
Z	4

ρ_{calc}g/cm^3	1.908
μ/mm^{-1}	3.855
F(000)	1816.0
Crystal size/mm^3	0.63 × 0.587 × 0.177
Radiation	MoKα (λ = 0.71073)
2Θ range for data collection/°	2.132 to 57.874
Index ranges	$-25 \leq h \leq 25$, $-13 \leq k \leq 13$, $-23 \leq l \leq 23$
Reflections collected	50087
Independent reflections	8587 [R_{int} = 0.0551, R_{sigma} = 0.0375]
Data/restraints/parameters	8587/7/347
Goodness-of-fit on F^2	1.076
Final R indexes [I>=2σ (I)]	R_1 = 0.0379, wR_2 = 0.1013
Final R indexes [all data]	R_1 = 0.0431, wR_2 = 0.1061
Largest diff. peak/hole / e Å^{-3}	1.94/-0.70

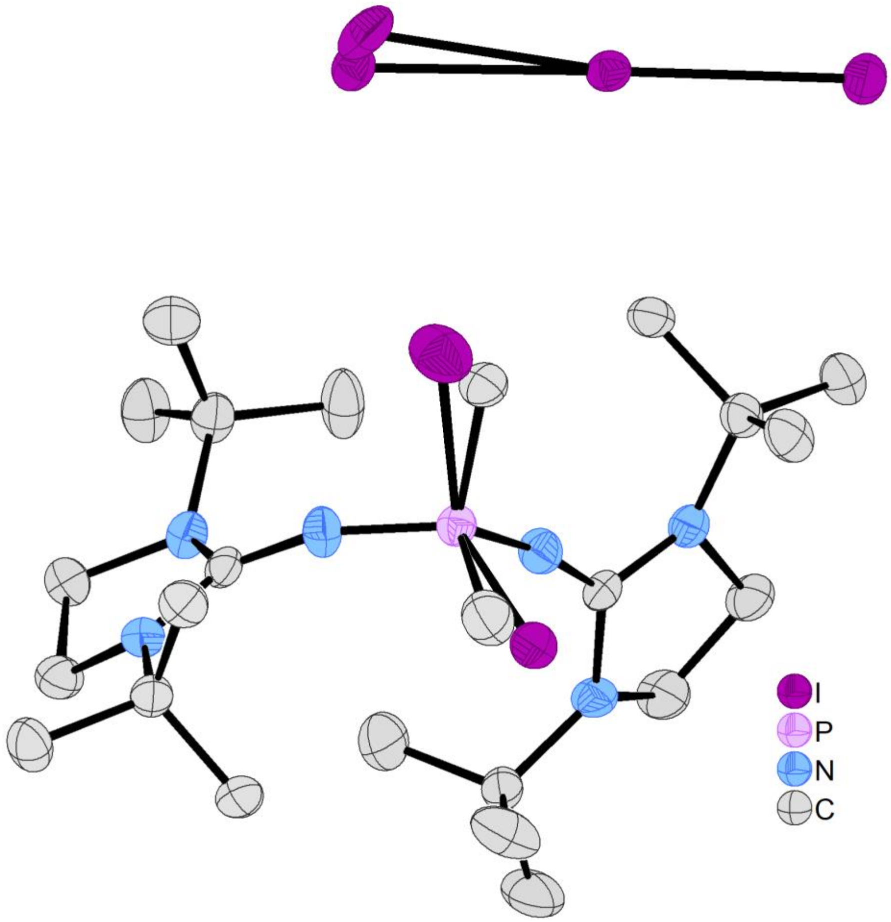

Figure S89: The asymmetric unit contains one molecule of [**3a**]I_3. The phosphorus bound methyl and iodine are disordered over two positions in a ratio of 24:1. One iodine atom from the I_3^- anion is also disordered over two positions in a ratio of 24:1

2.5 Crystal structure data of compound [3b]OTf

CCDC deposition number	**2244199**
Empirical formula	$C_{117.75}H_{157}F_{12}N_{12}O_{12.5}P_2S_4$
Formula weight	2358.72
Temperature/K	100(2)
Crystal system	monoclinic
Space group	$P2_1/c$
a/Å	13.5828(4)
b/Å	48.1844(12)
c/Å	19.0661(5)
α/°	90
β/°	92.090(2)
γ/°	90
Volume/Å^3	12470.1(6)
Z	4

ρ_{calc}g/cm^3	1.256
μ/mm^{-1}	0.181
F(000)	4948.0
Crystal size/mm^3	0.400 × 0.300 × 0.050
Radiation	MoKα (λ = 0.71073)
2Θ range for data collection	2.298 to 52.9°
Index ranges	-16 ≤ h ≤ 16, -60 ≤ k ≤ 60, -23 ≤ l ≤ 23
Reflections collected	120521
Independent reflections	25293 [R_{int} = 0.0677, R_{sigma} = 0.0643]
Data/restraints/parameters	25293/552/1707
Goodness-of-fit on F^2	1.130
Final R indexes [I>=2σ (I)]	R_1 = 0.0981, wR_2 = 0.2195
Final R indexes [all data]	R_1 = 0.1184, wR_2 = 0.2281
Largest diff. peak/hole / e Å^{-3}	0.98/-0.60

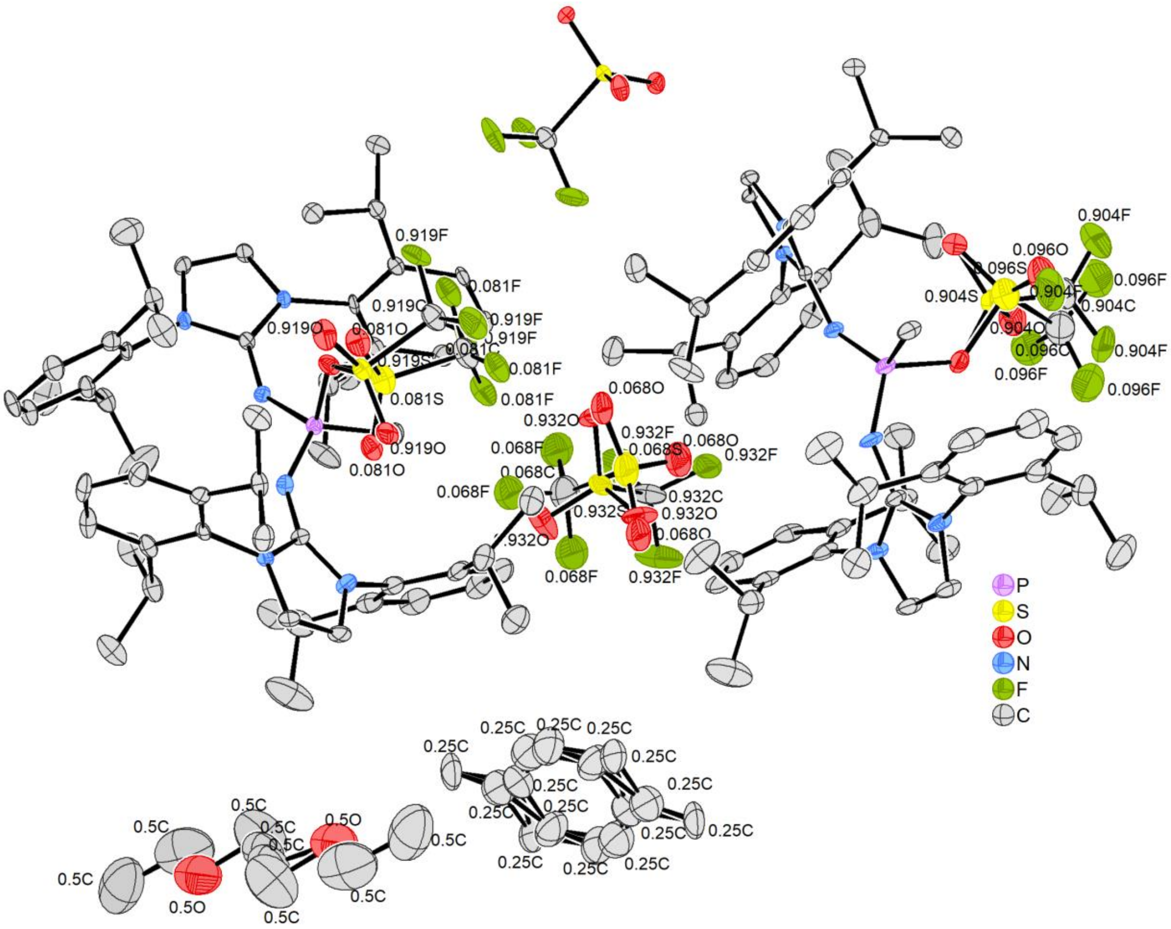

Figure S90: The asymmetric unit contains two molecules of [**3b**]OTf, half a molecule of toluene and a quarter molecule of diethyl ether. Both solvent molecules are located close to an inversion center. Some triflate and solvent fragments are disordered over two positions (see atom labels).

2.6 Crystal structure data of compound [4a]I

CCDC deposition number	**2244191**
Empirical formula	$C_{23}H_{48}IN_6P$
Formula weight	566.54
Temperature/K	100
Crystal system	monoclinic
Space group	*C*2/*c*
a/Å	23.1223(6)
b/Å	10.6267(3)
c/Å	11.3533(3)
α/°	90
β/°	98.0870(10)
γ/°	90
Volume/Å^3	2761.92(13)
Z	4
ρ_{calc}g/cm^3	1.362
μ/mm^{-1}	1.239
F(000)	1184.0
Crystal size/mm^3	0.369 × 0.275 × 0.14
Radiation	MoKα (λ = 0.71073)
2Θ range for data collection/°	3.558 to 58.936
Index ranges	$-31 \leq h \leq 31$, $-14 \leq k \leq 14$, $-15 \leq l \leq 15$
Reflections collected	22617
Independent reflections	3829 [R_{int} = 0.0407, R_{sigma} = 0.0245]
Data/restraints/parameters	3829/12/173
Goodness-of-fit on F^2	1.240
Final R indexes [I>=2σ (I)]	R_1 = 0.0405, wR_2 = 0.0971
Final R indexes [all data]	R_1 = 0.0431, wR_2 = 0.0985
Largest diff. peak/hole / e Å^{-3}	1.57/-0.97

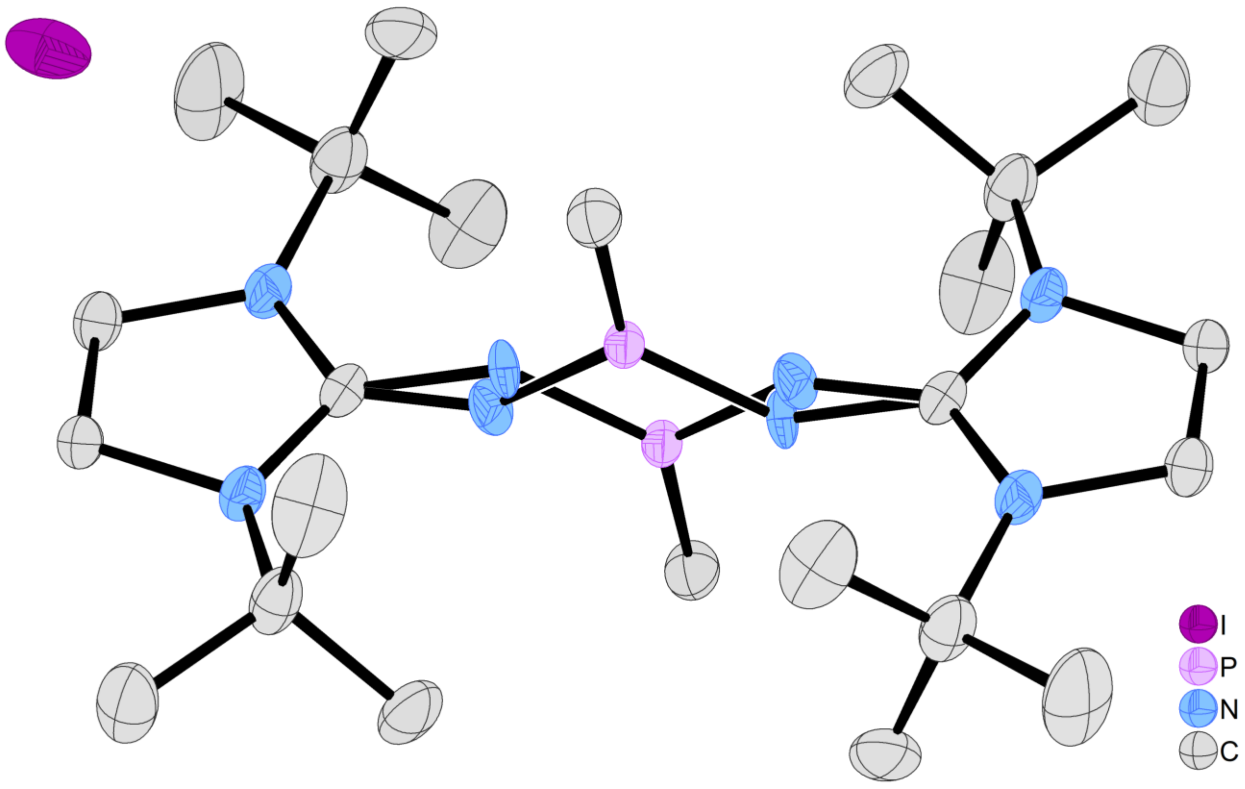

Figure S91: The asymmetric unit contains one molecule of [**4a**]I. The $N_2P{=}CH_2$ fragment is disordered over two positions in a ratio of 1:1.

2.7 Crystal structure data of compound [4a’]($BArF_{24}$)$_2$

CCDC deposition number	**2244197**	ρ_{calc}g/cm^3	1.428
Empirical formula	$C_{110}H_{118}B_2F_{48}N_{12}OP_2$	μ/mm^{-1}	0.161
Formula weight	2619.72	F(000)	2684.0
Temperature/K	100	Crystal size/mm^3	0.439 × 0.382 × 0.356
Crystal system	triclinic	Radiation	MoKα (λ = 0.71073)
Space group	$P\bar{1}$	2Θ range for data collection/°	3.972 to 52.898
a/Å	13.7117(5)	Index ranges	-16 ≤ h ≤ 16, -21 ≤ k ≤ 21, -36 ≤ l ≤ 36
b/Å	16.9823(9)	Reflections collected	67073
c/Å	29.5424(8)	Independent reflections	24114 [R_{int} = 0.0549, R_{sigma} = 0.0542]
α/°	74.076(3)	Data/restraints/parameters	24114/716/1809
β/°	82.009(3)	Goodness-of-fit on F^2	1.062
γ/°	67.187(4)	Final R indexes [I>=2σ (I)]	R_1 = 0.0545, wR_2 = 0.1386
Volume/Å^3	6093.6(5)	Final R indexes [all data]	R_1 = 0.0664, wR_2 = 0.1503
Z	2	Largest diff. peak/hole / e Å^{-3}	0.72/-0.53

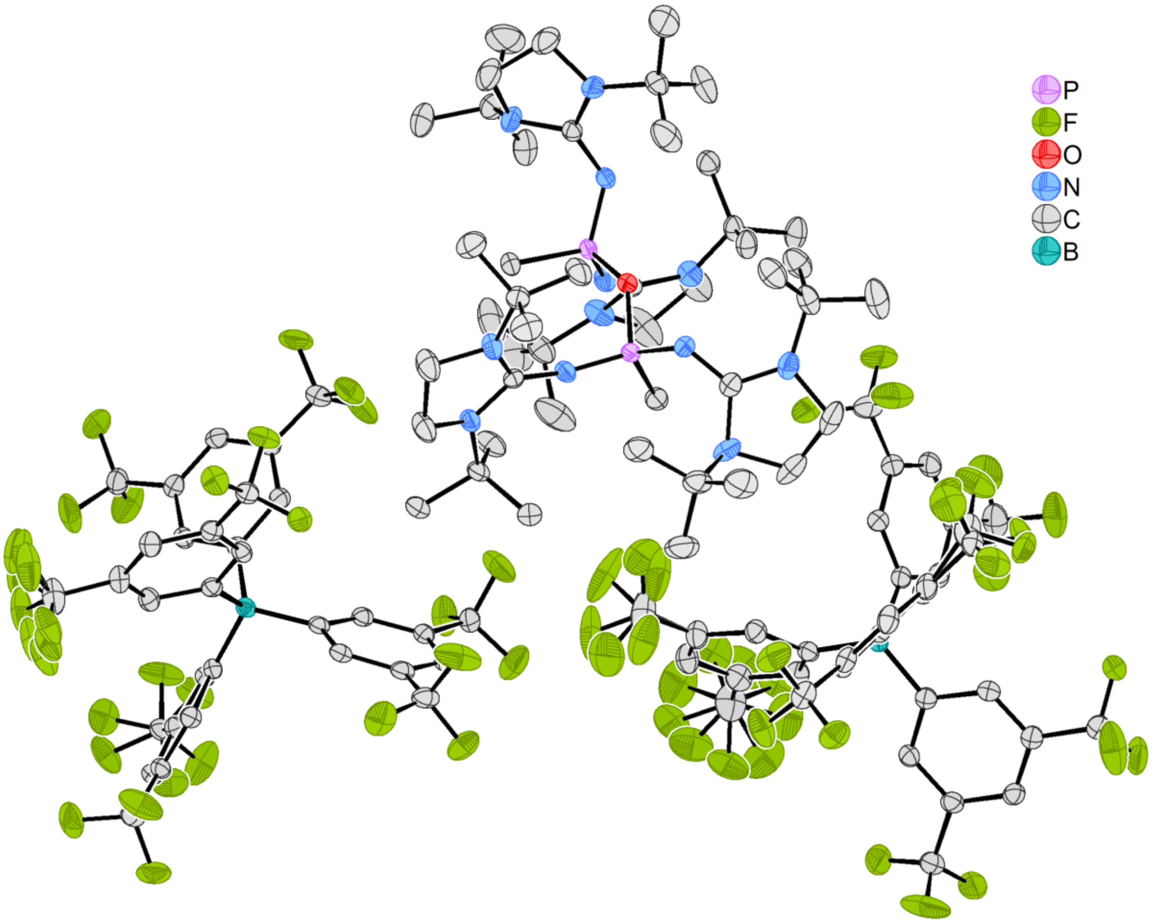

Figure S92: The asymmetric unit contains one molecule of [**4a‘**]($BArF_{24}$)$_2$. Five of the CF_3-fragments are disordered, of which four show a rotational disorder over two or three positions (0.78:0.22; 0.59:0.41; 0.87:0.13; 0.39:0.37:0.24; 0.43:0.31:0.26).

2.8 Crystal structure data of compound [4b]OTf

CCDC deposition number	**2244194**	ρ_{calc}g/cm^3	1.183
Empirical formula	$C_{56}H_{74}F_3N_6O_3PS$	μ/mm^{-1}	0.143
Formula weight	999.24	F(000)	2136.0
Temperature/K	100	Crystal size/mm^3	0.29 × 0.29 × 0.05
Crystal system	triclinic	Radiation	MoKα (λ = 0.71073)
Space group	$P\bar{1}$	2Θ range for data collection	1.988 to 52.834°
a/Å	12.1219(2)	Index ranges	-15 ≤ h ≤ 15, -26 ≤ k ≤ 26, -28 ≤ l ≤ 28
b/Å	21.3779(3)	Reflections collected	74124
c/Å	22.6000(3)	Independent reflections	23011 [R_{int} = 0.0357, R_{sigma} = 0.0395]
α/°	106.5338(7)	Data/restraints/parameters	23011/555/1485
β/°	90.8992(7)	Goodness-of-fit on F^2	1.019
γ/°	91.4357(7)	Final R indexes [I>=2σ (I)]	R_1 = 0.0529, wR_2 = 0.1282
Volume/Å^3	5611.07(14)	Final R indexes [all data]	R_1 = 0.0741, wR_2 = 0.1390
Z	4	Largest diff. peak/hole / e Å^{-3}	0.79/-0.44

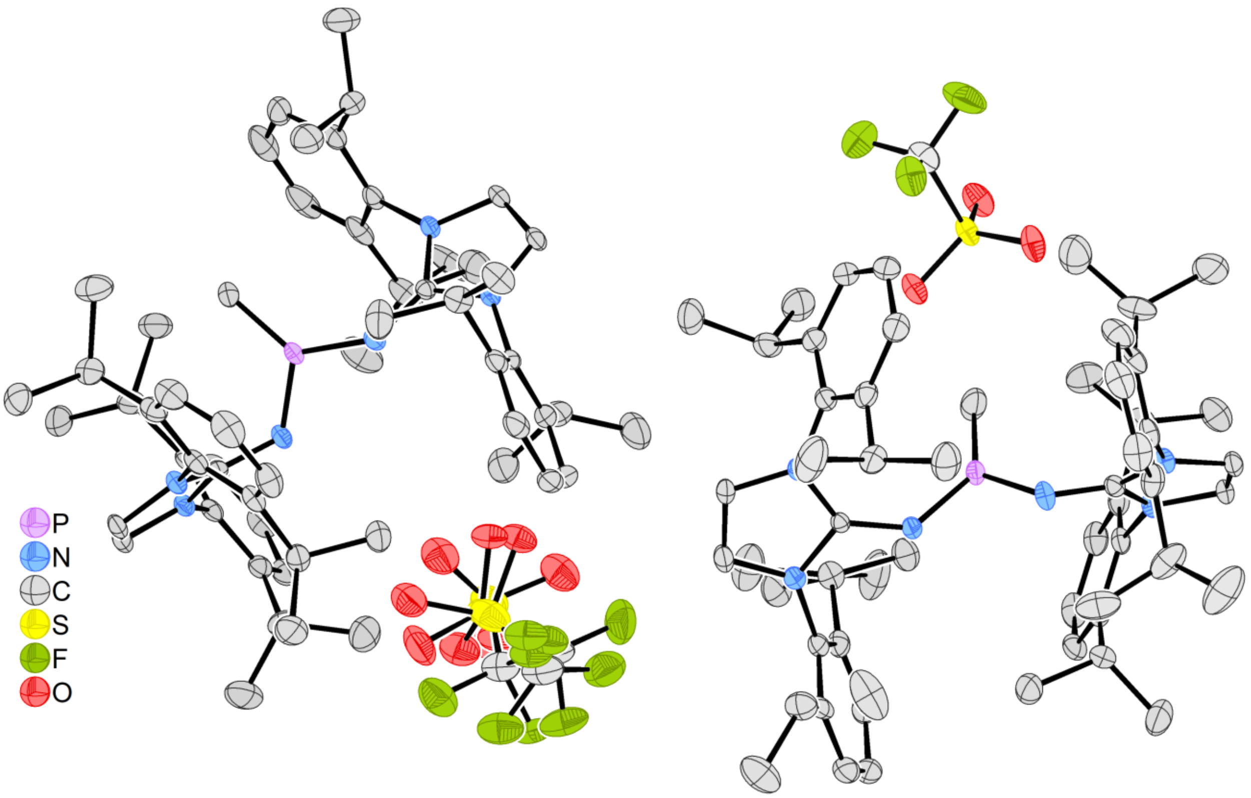

Figure S93: The asymmetric unit contains two molecules of [**4b**]OTf. One isopropyl group is disordered over two positions with a ratio of 3:7 and one OTf$^-$ anion is disordered over three positions (0.71:0.15:0.14).

2.9 Crystal structure data of compound 8

CCDC deposition number	**2244193**	ρ_{calc}g/cm^3	1.099
Empirical formula	$C_{55}H_{73}N_6P$	μ/mm^{-1}	0.094
Formula weight	849.16	F(000)	1840.0
Temperature/K	100	Crystal size/mm^3	0.272 × 0.143 × 0.112
Crystal system	monoclinic	Radiation	MoKα (λ = 0.71073)
Space group	$P2_1/n$	2Θ range for data collection	2.898 to 56.692°
a/Å	12.9765(2)	Index ranges	-17 ≤ h ≤ 17, -26 ≤ k ≤ 26, -26 ≤ l ≤ 26
b/Å	20.2016(4)	Reflections collected	73164
c/Å	20.2590(4)	Independent reflections	12743 [R_{int} = 0.0642, R_{sigma} = 0.0460]
α/°	90	Data/restraints/parameters	12743/18/626
β/°	104.9440(10)	Goodness-of-fit on F^2	1.036
γ/°	90	Final R indexes [I>=2σ (I)]	R_1 = 0.0606, wR_2 = 0.1412
Volume/Å^3	5131.20(17)	Final R indexes [all data]	R_1 = 0.0869, wR_2 = 0.1534
Z	4	Largest diff. peak/hole / e Å^{-3}	0.49/-0.32

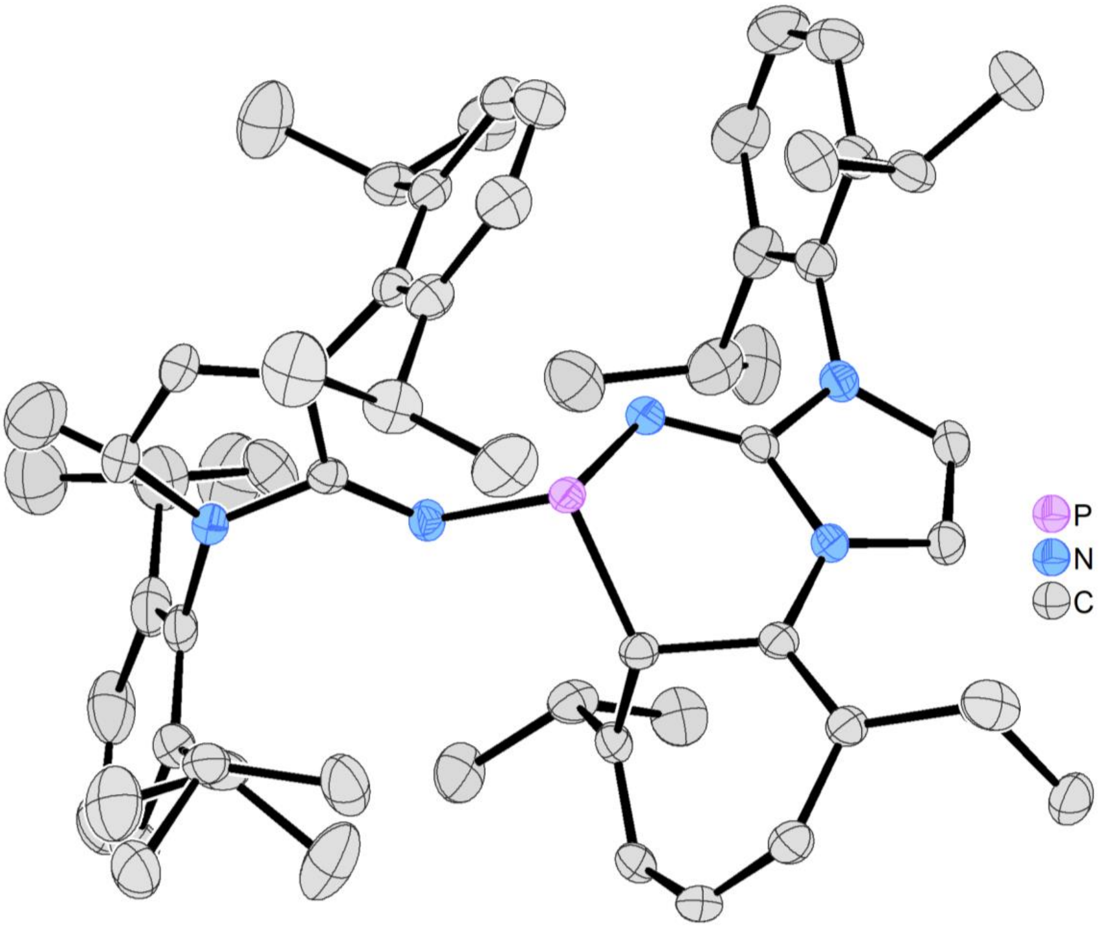

Figure S94: The asymmetric unit contains one molecule of **8**. Two isopropyl groups are disordered over two positions with the ratios of 22:3 and 73:27, respectively.

3 Computational studies

3.1 General

All calculations were executed using Gaussian16[9] employing the B3LYP[10] functional in conjunction with def2-TZVP[11] basis sets and empirical GD3BJ dispersion correction.[12] Calculations in implicit solvent were done employing the SMD solvation model.[13]

3.2 Optimized structures of structures G-I

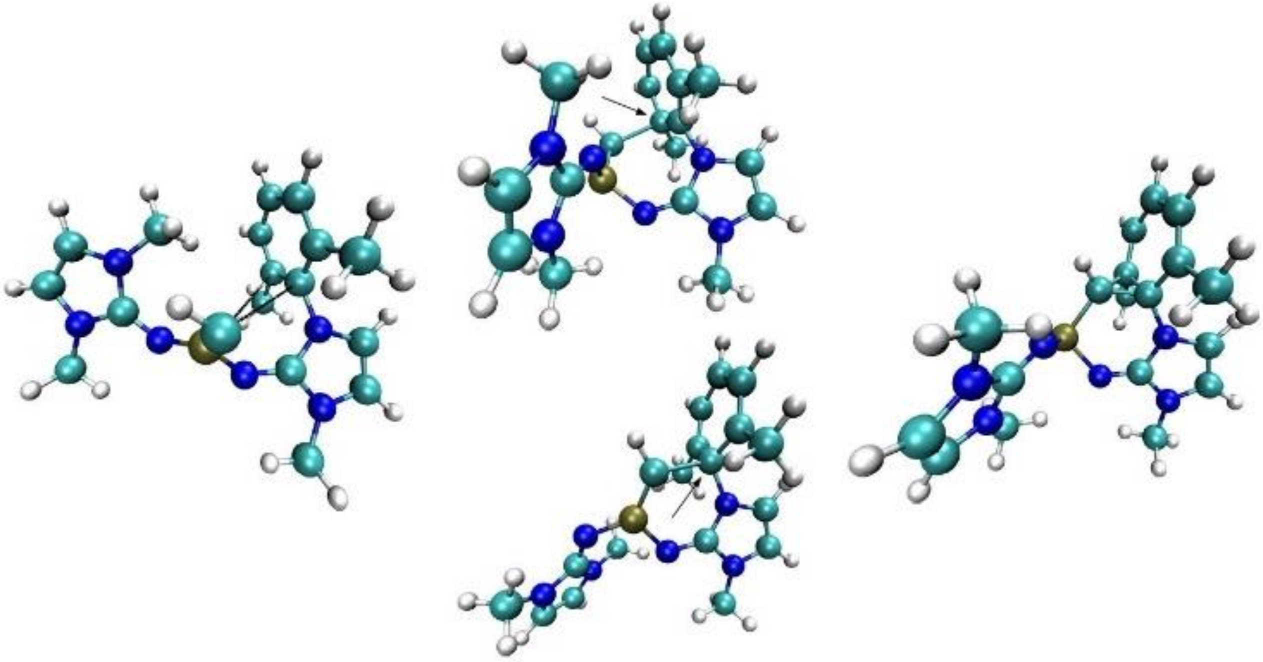

Figure S95: Optimized geometries of the reaction **G**(left)→ $\mathbf{GH}^{\ddagger}_{1\text{-}2}$ → **H**(right) obtained at B3LYP-GD3BJ/def2-TZVP level of theory, the upper transition state is targeting the methyl-bound carbon atom of the phenyl ring, the lower is targeting the nitrogen-bound carbon leading to the cyclopropane formation in **H**.

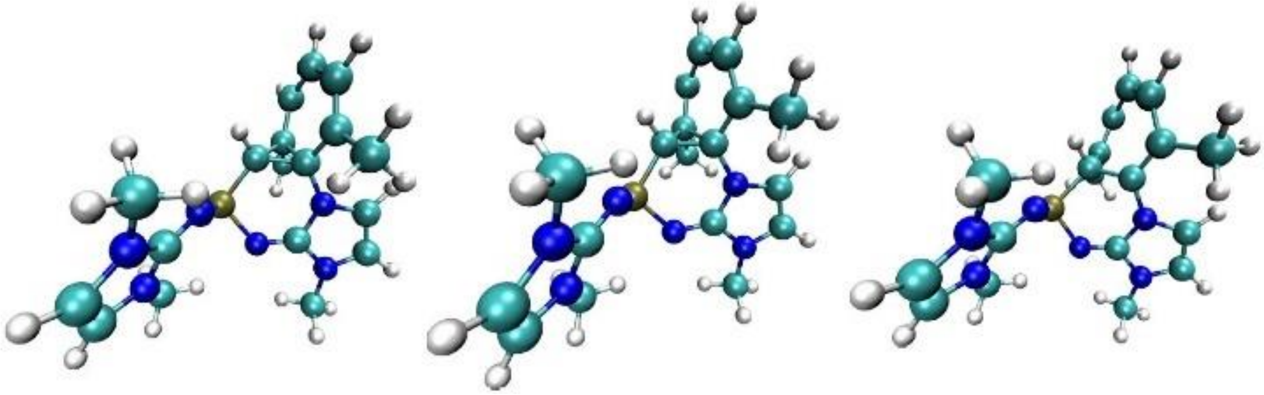

Figure S96: Optimized geometries of the reaction **H**(left)→ **HI**‡ → **I**(right) obtained at B3LYP-GD3BJ/def2-TZVP level of theory.

Table S2: Electronic energy ΔE and estimated Gibbs-free $\Delta G°$ energy relative to compound I in kJ·mol^{-1} obtained at B3LYP-GD3BJ/def2-TZVP level of theory.

	ΔE		$\Delta G°$	
Geometry	vac	impl. solv.	vac	impl. solv.
G	130.2	117.9	120.4	101.8
$GH_1^{‡}$	225.2	226.0	215.6	215.4
$GH_2^{‡}$	214.1	209.9	204.0	199.7
H	39.8	40.9	38.6	37.5
HI‡	49.5	45.3	44.4	50.1
I	0	0	0	0

Table S3: Variation of the C–P$_{carbene}$ bond distance r_{PC} in Å, the sum of X–P–X angles (X = C, N) $\sum_{i=1}^{3}\alpha_{i,X-P-X}$ in degree and Mulliken atomic partial charges of the C$_{carbene}$ and P atoms q_C and q_P obtained at B3LYP-GD3BJ/def2-TZVP level in vacuum (vac.) and implicit solvation (impl. solv.), respectively.

	r_{PC}		$\sum_{i=1}^{3}\alpha_{i,X-P-X}$		q_C		q_P	
**Geometry**	vac.	impl. solv.	vac.	impl. solv.	vac.	impl. solv.	vac.	impl. solv.
**G**	1.606	1.613	359.6	359.4	-0.526	-0.703	0.304	0.357
$GH_1^{‡}$	1.693	1.692	331.4	333.7	-0.330	-0.330	0.256	0.256
$GH_2^{‡}$	1.603	1.601	359.6	360.0	-0.413	-0.388	0.398	0.410
**H**	1.855	1.854	300.1	302.1	-0.142	-0.160	0.273	0.234
$HI^{‡}$	1.859	1.859	299.9	301.6	-0.002	-0.010	0.249	0.211
**I**	1.870	1.870	300.2	302.1	0.150	0.183	0.239	1.196

3.3 Cartesian Coordinates

Molecular geometries of the optimised structures (A-F) in the singlet state with respective minimum energies in Hartree in the xyz-file format:

A (singlet)
Number of atoms: 3
Energy: -39.1513425355

C	-0.000000	-0.000000	0.175054
H	0.000000	-0.862090	-0.525161
H	0.000000	0.862090	-0.525161

B (singlet)
Number of atoms: 15
Energy: -196.510584942

C	-0.091461	-0.003916	0.126501
C	-0.758697	-1.353456	-0.144359
H	-1.522112	-1.548281	0.611570
H	-1.251880	-1.369137	-1.119614
H	-0.032673	-2.166988	-0.108845
C	1.193941	0.212033	-0.783510
H	1.352110	-0.667602	-1.400070
H	1.076969	1.124067	-1.360227
H	2.110897	0.377726	-0.178428
C	-1.080684	1.151018	-0.006838
H	-1.889152	1.033135	0.717563
H	-0.586442	2.100418	0.195372
H	-1.526860	1.182270	-1.004298
C	0.892304	0.128898	1.192307
H	1.336724	-0.873070	1.342372

C (singlet)
Number of atoms: 11
Energy: -173.276627611

C	-1.091027	-0.807222	0.000000
H	-2.050929	-0.299223	0.000000
H	-1.011629	-1.442491	-0.885040
H	-1.011629	-1.442491	0.885040
C	1.323636	-0.397499	-0.000000
H	1.455228	-1.023012	-0.886253
H	2.048676	0.409032	0.000001
H	1.455227	-1.023013	0.886253
C	-0.155222	1.493051	0.000000
H	-1.242676	1.696617	-0.000000
N	-0.015226	0.199229	-0.000000

D (singlet)
Number of atoms: 11
Energy: -459.878929952

C	-1.721571	-0.080755	0.000000
H	-2.226630	0.883184	-0.000004
H	-2.017675	-0.643945	-0.886975
H	-2.017672	-0.643941	0.886978
C	0.885059	-1.424733	0.000000
H	0.597070	-1.994542	-0.884823
H	1.961437	-1.268730	0.000004
H	0.597060	-1.994538	0.884823
C	1.032052	1.519819	-0.000001
H	0.510506	2.477604	0.000007
P	0.094844	0.206595	-0.000001

E (singlet)
Number of atoms: 35
Energy: -1099.55126705

C	4.786759	0.391406	-0.156149
C	4.467641	-0.893912	-0.402808
N	3.601708	1.084209	0.033864
C	2.525287	0.232495	-0.102033
N	3.083083	-1.001820	-0.366377
C	3.470204	2.490341	0.338883
H	2.410086	2.695827	0.465314
H	3.860645	3.101920	-0.476743
H	4.002622	2.734545	1.259419
C	2.328291	-2.176466	-0.756113
H	3.029107	-2.932330	-1.105157
H	1.643742	-1.922826	-1.567099
H	1.737436	-2.560832	0.074465
N	1.288454	0.612816	-0.042230
P	0.019415	-0.237687	0.575272
N	-1.243253	0.445702	-0.175713
C	-2.517031	0.204185	-0.118723
N	-3.468236	1.196730	-0.235600
C	-4.737748	0.644569	-0.226544
C	-4.591441	-0.689624	-0.104539
N	-3.233747	-0.965590	-0.031609
C	-3.146347	2.602437	-0.323777
H	-3.558150	3.032978	-1.238082
H	-2.062396	2.685023	-0.340951
H	-3.539647	3.144010	0.538463
H	-5.623611	1.248814	-0.294164
H	-5.326306	-1.471752	-0.055391
C	-2.659679	-2.299784	-0.068965
H	-1.837066	-2.357631	0.650926
H	-2.286955	-2.521569	-1.072091
H	-3.441367	-3.012706	0.186646
C	-0.118423	-1.397504	1.685666
H	0.819344	-1.679373	2.166877
H	5.745326	0.872075	-0.089001
H	5.094824	-1.744058	-0.598263

F (singlet)
Number of atoms: 39
Energy: -1101.98101155

N	3.491169	1.183823	-0.159459
C	2.512457	0.213116	-0.065808
N	3.119435	-1.015852	-0.241904
C	3.292637	2.500881	0.395773
H	2.238244	2.751480	0.314442
H	3.885435	3.228168	-0.161036
H	3.585099	2.544400	1.452882
C	2.415431	-2.169885	-0.760689
H	3.040324	-3.051399	-0.615347
H	2.200885	-2.064959	-1.831950
H	1.482482	-2.317902	-0.221410
N	1.281477	0.517903	0.133276
P	0.013204	-0.369064	0.701570
N	-1.244420	0.406013	0.031214
C	-2.507824	0.198489	-0.084857
N	-3.359685	1.232632	-0.434415
N	-3.259244	-0.940404	0.048165
C	-2.984679	2.609945	-0.224669
H	-3.502421	3.245343	-0.944939
H	-1.910832	2.698495	-0.366293
H	-3.236469	2.950360	0.788447
C	-2.722352	-2.279729	-0.079136
H	-1.835529	-2.378710	0.549898
H	-2.475909	-2.513947	-1.122868
H	-3.479236	-2.984792	0.265500
C	-0.130943	-1.565999	1.769394
H	0.814991	-1.910276	2.190140
C	4.502883	-0.825427	-0.660847
H	4.584306	-0.868786	-1.755405
H	5.151926	-1.590734	-0.235179
C	4.808191	0.576021	-0.143477
H	5.213929	0.544742	0.877438
H	5.509918	1.120736	-0.776047
C	-4.739980	0.821371	-0.260793
H	-5.390750	1.270336	-1.011977
H	-5.111332	1.104764	0.734201
C	-4.624219	-0.692977	-0.396691
H	-5.341045	-1.235828	0.219302
H	-4.752812	-1.009161	-1.440683

Molecular Geometries of the optimised structures (A-F) in the triplet state with respective minimum energies in Hartree in the xyz-file format:

A (singlet)
Number of atoms: 3
Energy: -39.1700142664

C	-0.000000	-0.000000	0.103094
H	0.000000	-0.996274	-0.309282
H	0.000000	0.996274	-0.309282

B (triplet)
Number of atoms: 15
Energy: -196.513120812

C	0.003958	-0.000000	0.105920
C	0.584803	-1.259815	-0.575626
H	0.180263	-2.166067	-0.123894
H	0.332105	-1.261431	-1.639268
H	1.671914	-1.287469	-0.484845
C	0.584485	1.260001	-0.575552
H	0.331786	1.261618	-1.639193
H	0.179718	2.166123	-0.123763
H	1.671589	1.287924	-0.484769
C	-1.528147	-0.000188	-0.044287
H	-1.964422	-0.883662	0.423437
H	-1.964644	0.883145	0.423496
H	-1.801467	-0.000186	-1.101695
C	0.364355	-0.000003	1.536344
H	1.306427	0.000039	2.069696

C (triplet)
Number of atoms: 11
Energy: -173.217267662

C	-1.234697	-0.607574	0.032712
H	-2.074583	0.022345	-0.251658
H	-1.223047	-1.492930	-0.608842
H	-1.375522	-0.937352	1.070764
C	1.234705	-0.607558	0.032712
H	1.223055	-1.492925	-0.608826
H	2.074584	0.022362	-0.251678
H	1.375545	-0.937315	1.070769
C	-0.000011	1.493323	-0.120198
H	-0.000019	2.139437	0.766393
N	0.000001	0.143891	-0.122611

D (triplet)
Number of atoms: 11
Energy: -459.848635085

C	-1.430506	-0.745427	0.256946
H	-2.358276	-0.244143	-0.015773
H	-1.461785	-1.763833	-0.134873
H	-1.341074	-0.777602	1.343419
C	1.430477	-0.745478	0.256943
H	1.461712	-1.763889	-0.134868
H	2.358266	-0.244236	-0.015788
H	1.341049	-0.777643	1.343416
C	0.000034	1.684202	0.241939
H	0.000075	2.277230	1.144853

P	0.000000	0.142289	-0.537690

E (triplet)
Number of atoms: 35
Energy: -1099.51489563

C	4.465574	0.784169	0.001869
C	3.733623	1.263383	-1.022243
N	3.650424	-0.069306	0.725199
C	2.387969	-0.128615	0.158748
N	2.459385	0.719106	-0.929543
C	4.021033	-0.803930	1.911348
H	3.221881	-1.511892	2.116954
H	4.135444	-0.136948	2.769081
H	4.956588	-1.339986	1.746098
C	1.386248	1.003060	-1.866348
H	1.612823	1.945263	-2.363849
H	0.445133	1.089544	-1.327573
H	1.286006	0.204086	-2.600222
N	1.441150	-0.852505	0.656388
P	-0.043311	-1.351069	0.132973
N	-0.984370	0.047923	0.094385
C	-2.261764	0.195580	0.163988
N	-2.862265	1.413032	0.441926
C	-4.241872	1.288702	0.419183
C	-4.526938	0.000811	0.148797
N	-3.323396	-0.677036	-0.014099
C	-2.107317	2.621689	0.668191
H	-1.908101	3.151114	-0.267389
H	-1.155300	2.351521	1.120269
H	-2.663305	3.275721	1.338583
H	-4.893116	2.123668	0.601507
H	-5.472939	-0.500058	0.056279
C	-3.211981	-2.084972	-0.333841
H	-3.030713	-2.687042	0.557088
H	-2.388367	-2.238813	-1.029900
H	-4.139765	-2.407529	-0.805048
C	0.105579	-1.973828	-1.494728
H	-0.007561	-3.033430	-1.725653
H	5.486440	0.971470	0.279863
H	3.997096	1.944570	-1.810268

F (triplet)
Number of atoms: 39
Energy: -1101.94429938

N	3.617668	0.023938	0.880764
C	2.400534	-0.070481	0.218939
N	2.521806	0.614664	-0.965427
C	3.967513	-0.923600	1.911132
H	3.063605	-1.196917	2.448257
H	4.681757	-0.469670	2.600060
H	4.415144	-1.834705	1.491368
C	1.429120	1.170287	-1.732915
H	1.480904	2.265711	-1.701545
H	0.475651	0.860723	-1.321411
H	1.485073	0.845917	-2.773517
N	1.419822	-0.722546	0.729197
P	-0.019363	-1.315782	0.158565
N	-1.034223	0.033930	0.115985
C	-2.306798	0.151766	0.140728
N	-2.914936	1.396315	0.243464
N	-3.318446	-0.795196	0.090443
C	-2.196575	2.525204	0.781847
H	-2.603274	3.451329	0.372345
H	-1.150200	2.431485	0.503858
H	-2.263811	2.566122	1.877299
C	-3.162787	-2.102375	-0.510943
H	-2.228195	-2.554830	-0.195744
H	-3.182981	-2.054681	-1.606895
H	-3.980119	-2.740751	-0.172400
C	0.193317	-1.873517	-1.482817
H	0.107698	-2.916968	-1.781698
C	3.823874	1.252096	-1.066763
H	3.746694	2.319296	-0.818123
H	4.231815	1.166925	-2.075268
C	4.645495	0.494228	-0.028210
H	5.179721	-0.352067	-0.483579
H	5.376087	1.125200	0.479666
C	-4.330227	1.245935	0.519252
H	-4.918075	2.033314	0.045911
H	-4.527523	1.268840	1.600943
C	-4.607105	-0.134921	-0.062701
H	-5.391662	-0.674195	0.468882
H	-4.887864	-0.068807	-1.123169

Molecular Geometries of the optimized structures (G-I) in vacuum with respective minimum energies in Hartree in the xyz-file format:

G in vac
Number of atoms: 48
Energy: -1370.05720185

C	5.172996	0.155966	-0.157132
C	4.593877	-1.013954	0.178039
N	4.164506	1.099459	-0.270276
C	2.941365	0.519606	-0.011317
N	3.225683	-0.798586	0.271721
C	4.329736	2.498508	-0.592752
H	3.341422	2.951634	-0.581576
H	4.770066	2.616414	-1.584472
H	4.965564	2.990489	0.144990
C	2.224465	-1.835907	0.436144
H	2.710470	-2.802964	0.321386

H	1.458808	-1.731928	-0.329568
H	1.734540	-1.771400	1.405957
N	1.807527	1.144069	-0.100631
P	0.489782	0.934916	0.863560
N	-0.685310	1.695889	0.022435
C	-1.934561	1.368997	-0.011173
N	-2.969320	2.272005	-0.080969
C	-4.181445	1.606524	-0.196496
C	-3.920999	0.286527	-0.195828
N	-2.541092	0.128440	-0.079443
C	-2.782080	3.702387	0.004866
H	-3.091557	4.190527	-0.921273
H	-1.722092	3.880341	0.167819
H	-3.353894	4.112345	0.838440
H	-5.115593	2.133805	-0.258988
H	-4.575026	-0.561519	-0.274738
C	0.212338	0.356207	2.335594
H	1.077484	-0.056899	2.853569
H	6.206195	0.403526	-0.317130
H	5.026314	-1.980795	0.358368
C	-1.869831	-1.119017	-0.269551
C	-1.886147	-2.061725	0.763212
C	-1.270462	-1.370031	-1.509025
C	-1.290559	-3.297769	0.518835
C	-0.682093	-2.617737	-1.707345
C	-0.698304	-3.576714	-0.704407
H	-1.288286	-4.042342	1.304753
H	-0.217034	-2.835332	-2.660734
H	-0.243930	-4.544504	-0.876289
C	-1.253925	-0.328809	-2.591483
H	-0.903130	-0.757464	-3.529340
H	-2.248829	0.089773	-2.756202
H	-0.596562	0.500087	-2.319228
C	-2.458262	-1.724619	2.105390
H	-3.487571	-1.368301	2.039691
H	-2.436478	-2.593751	2.762003
H	-1.848220	-0.925041	2.545880

$GH_1^‡$ in vac
Number of atoms: 48
Energy: -1370.02104283

C	4.548777	-0.949969	0.748119
C	4.688771	0.193883	0.051416
N	3.242208	-1.379802	0.582198
C	2.541000	-0.495601	-0.221880
N	3.463921	0.487832	-0.537679
C	2.650626	-2.571397	1.144114
H	2.479530	-2.460852	2.217214
H	3.299452	-3.430801	0.970825
H	1.694632	-2.725167	0.648438
C	3.223282	1.623766	-1.406759
H	3.979790	2.379521	-1.198470
H	2.236999	2.041959	-1.209683
H	3.283480	1.339498	-2.458504
N	1.297477	-0.661633	-0.520618
P	0.334678	0.147923	-1.638703
N	-0.070314	1.615699	-0.916093
C	-0.851344	1.711087	0.107097
N	-1.032976	2.882216	0.815062
C	-1.918122	2.679267	1.865006
C	-2.314715	1.395641	1.820794
N	-1.666573	0.786311	0.742883
C	-0.322098	4.099228	0.506811
H	-0.171624	4.149870	-0.569456
H	0.656132	4.126970	0.994394
H	-0.910842	4.953892	0.837172
H	-2.196302	3.472137	2.534837
H	-3.017947	0.854342	2.424841
H	5.256575	-1.493232	1.346865
H	5.543219	0.832476	-0.077564
C	-1.060082	-0.683596	-2.118341
H	-0.868177	-1.717197	-2.409916
C	-1.950088	-0.541107	0.299387
C	-2.643510	-0.698321	-0.948856
C	-1.571622	-1.610131	1.111267
C	-3.084301	-2.035740	-1.210959
C	-1.978423	-2.891076	0.751998
C	-2.739702	-3.091754	-0.402259
H	-3.667971	-2.202708	-2.107391
H	-1.691130	-3.733361	1.367964
H	-3.068183	-4.090749	-0.660855
C	-0.735940	-1.386664	2.341939
H	-1.296084	-0.895843	3.140882
H	-0.373856	-2.338629	2.728908
H	0.122721	-0.756877	2.107936
C	-3.467458	0.447529	-1.490209
H	-3.830797	0.194020	-2.484361
H	-4.326499	0.639813	-0.838752
H	-2.879802	1.357432	-1.577161

$GH_2^‡$ in vac
Number of atoms: 48
Energy: -1370.02524714

C	-5.396767	-0.213740	0.069498
C	-4.787406	0.340550	1.137274
N	-4.429015	-0.897210	-0.647777
C	-3.209083	-0.770089	-0.031774
N	-3.445419	-0.003685	1.080641
C	-4.631486	-1.626376	-1.881770
H	-4.954466	-0.952611	-2.676538
H	-5.379403	-2.407536	-1.742448
H	-3.681396	-2.078945	-2.153245

C	-2.459727	0.278312	2.107397
H	-2.980468	0.598725	3.006930
H	-1.764223	1.043869	1.771730
H	-1.898175	-0.628974	2.326131
N	-2.118957	-1.359013	-0.438709
P	-0.612481	-0.785764	-0.592196
N	-0.588282	0.829950	-0.375190
C	0.586213	1.428064	-0.339855
N	0.698229	2.794483	-0.336955
C	2.034284	3.153422	-0.222506
C	2.747383	2.013212	-0.175198
N	1.866158	0.943210	-0.272374
C	-0.433294	3.690832	-0.364109
H	-1.270388	3.169814	-0.821907
H	-0.183791	4.574253	-0.950789
H	-0.720983	4.001489	0.643825
H	2.346946	4.181175	-0.202909
H	3.803027	1.839971	-0.083969
H	-6.423849	-0.182653	-0.244134
H	-5.184032	0.942003	1.934229
C	0.832732	-1.460972	-0.752185
H	1.067660	-2.506560	-0.868269
C	2.367875	-0.439396	-0.034145
C	2.478353	-0.781452	1.357787
C	3.472834	-0.800127	-0.889481
C	3.461487	-1.670746	1.763340
C	4.419522	-1.688373	-0.426408
C	4.428198	-2.154993	0.890995
H	3.482201	-1.970071	2.805961
H	5.188487	-2.020841	-1.115606
H	5.193371	-2.838259	1.232061
C	3.424151	-0.383764	-2.326833
H	3.442110	0.701331	-2.460775
H	4.262049	-0.804101	-2.883349
H	2.487542	-0.735606	-2.777017
C	1.474649	-0.244594	2.333744
H	1.696156	-0.592877	3.342693
H	1.459564	0.850369	2.357173
H	0.460759	-0.570264	2.081086

H in vac
Number of atoms: 48
Energy: -1370.09164374

C	4.909894	-1.038935	0.728499
C	5.009073	0.113330	0.039154
N	3.591913	-1.458418	0.647281
C	2.842681	-0.567769	-0.105436
N	3.749409	0.419914	-0.467392
C	3.015751	-2.648875	1.221844
H	2.150624	-2.389117	1.832789
H	3.766237	-3.140288	1.838070
H	2.679762	-3.333571	0.441071
C	3.464914	1.576572	-1.293183
H	4.208307	2.343701	-1.076213
H	2.476176	1.967398	-1.057377
H	3.506882	1.331361	-2.355650
N	1.586101	-0.738306	-0.318584
P	0.570140	0.093566	-1.391548
N	0.215679	1.561353	-0.590921
C	-0.785742	1.705599	0.205994
N	-1.143421	2.892687	0.816602
C	-2.351154	2.737969	1.487838
C	-2.751509	1.464382	1.323523
N	-1.774813	0.809554	0.575119
C	-0.372146	4.104964	0.690421
H	0.187672	4.052964	-0.241515
H	0.334822	4.220730	1.516028
H	-1.040914	4.965051	0.669477
H	-2.811876	3.549700	2.020197
H	-3.628844	0.952780	1.672147
H	5.652680	-1.592288	1.273138
H	5.855933	0.751665	-0.133527
C	-0.906927	-0.949505	-0.975444
H	-0.607634	-1.986717	-0.902427
C	-1.868520	-0.537882	0.101405
C	-2.334452	-0.721059	-1.390865
C	-2.257088	-1.550285	1.099596
C	-3.212152	-1.882488	-1.600123
C	-2.947386	-2.639107	0.703286
C	-3.453593	-2.792182	-0.640384
H	-3.714867	-1.953669	-2.558345
H	-3.162992	-3.416971	1.425946
H	-4.095843	-3.638201	-0.851464
C	-1.756484	-1.374736	2.502684
H	-2.206053	-0.517378	3.008041
H	-1.965760	-2.264531	3.095818
H	-0.676195	-1.201526	2.496761
C	-2.690875	0.507496	-2.200317
H	-2.713979	0.246517	-3.260492
H	-3.677598	0.884891	-1.923747
H	-1.968416	1.311571	-2.081172

$HI_1^{\ddagger}$ in vac
Number of atoms: 48
Energy: -1370.08793913

C	-4.866495	-1.050912	-0.752835
C	-4.984328	0.101823	-0.067220
N	-3.543770	-1.453503	-0.663794
C	-2.812513	-0.555718	0.097654
N	-3.732046	0.423645	0.448115
C	-2.956654	-2.649968	-1.215641
H	-1.997250	-2.405393	-1.669808

H	-3.628791	-3.059860	-1.967464
H	-2.779911	-3.398528	-0.439918
C	-3.465756	1.583067	1.275889
H	-4.214290	2.343218	1.052586
H	-2.479019	1.983886	1.048431
H	-3.514370	1.337826	2.338089
N	-1.557389	-0.712179	0.328441
P	-0.556946	0.126465	1.404647
N	-0.199846	1.595369	0.606597
C	0.767386	1.706759	-0.232554
N	1.150353	2.885762	-0.842517
C	2.310561	2.685994	-1.580058
C	2.660164	1.394069	-1.461852
N	1.691362	0.762391	-0.675040
C	0.447633	4.131464	-0.654373
H	-0.070046	4.084917	0.301745
H	-0.289982	4.300777	-1.443063
H	1.158995	4.956940	-0.647231
H	2.783495	3.486024	-2.119662
H	3.502106	0.858597	-1.854618
H	-5.598365	-1.613156	-1.303131
H	-5.839053	0.732299	0.095265
C	0.937133	-0.901195	0.993270
H	0.636634	-1.942702	0.987055
C	1.761250	-0.562064	-0.192304
C	2.308003	-0.673053	1.514901
C	2.211506	-1.612491	-1.066318
C	3.182683	-1.794002	1.687249
C	2.786432	-2.734516	-0.536679
C	3.283156	-2.818307	0.792787
H	3.840754	-1.778331	2.549473
H	3.004679	-3.557806	-1.206921
H	3.899953	-3.672693	1.043689
C	1.993109	-1.482312	-2.552035
H	1.990606	-2.473064	-3.006703
H	1.040787	-0.996994	-2.774089
H	2.772336	-0.902141	-3.052731
C	2.644780	0.630759	2.196091
H	2.913209	1.405180	1.473959
H	1.808406	1.012584	2.783945
H	3.504344	0.494660	2.852951

I in vac
Number of atoms: 48
Energy: -1370.10680475

C	4.738790	1.151292	-0.831772
C	4.907794	0.061566	-0.059664
N	3.398597	1.498122	-0.774207
C	2.707461	0.631126	0.059823
N	3.670633	-0.276547	0.481027
C	2.767594	2.627883	-1.411681
H	1.734983	2.364995	-1.633227
H	3.297838	2.865918	-2.333091
H	2.763628	3.506018	-0.760538
C	3.459199	-1.371200	1.406907
H	4.247942	-2.107446	1.252797
H	2.496710	-1.842678	1.213230
H	3.488394	-1.031663	2.443604
N	1.448717	0.748931	0.287880
P	0.476283	-0.071305	1.402428
N	0.225576	-1.609510	0.689384
C	-0.663133	-1.782868	-0.220125
N	-0.975309	-3.001426	-0.787665
C	-2.027875	-2.857288	-1.679542
C	-2.389611	-1.564475	-1.697245
N	-1.539643	-0.867864	-0.824152
C	-0.275888	-4.218912	-0.457555
H	0.060480	-4.151672	0.575311
H	0.598329	-4.366877	-1.097050
H	-0.950144	-5.066871	-0.571437
H	-2.435049	-3.695233	-2.215570
H	-3.186556	-1.078315	-2.221225
H	5.445296	1.701166	-1.426050
H	5.789695	-0.515946	0.148296
C	-1.093303	0.822343	0.917341
C	-1.639342	0.472101	-0.436382
C	-2.281728	0.771123	1.836130
C	-2.230341	1.445037	-1.189939
C	-3.162120	1.797056	1.743199
C	-2.703219	2.650153	-0.565554
C	-3.143860	2.801757	0.720783
H	-3.960284	1.862833	2.477041
H	-2.875289	3.487181	-1.235675
H	-3.634491	3.740449	0.956201
H	-0.764887	1.859637	0.853259
C	-2.472411	-0.390991	2.757899
H	-2.619788	-1.311768	2.184142
H	-1.587856	-0.553899	3.378433
H	-3.342664	-0.252196	3.399661
C	-2.458314	1.329509	-2.678232
H	-2.271904	2.300997	-3.141325
H	-1.789630	0.605478	-3.141479
H	-3.487140	1.056262	-2.933959

Molecular Geometries of the optimised structures (G-I) in implicit solvent (diethyl ether) with respective minimum energies in Hartree in the xyz-file format:

G in implicit solvent diethyl ether
Number of atoms: 48
Energy: -1370.09751186

C	5.120503	-0.327288	0.206403
C	4.640321	0.865710	-0.199682
N	4.042986	-1.190312	0.314674
C	2.883264	-0.537396	-0.011062
N	3.264755	0.739974	-0.332089
C	4.109043	-2.575856	0.730932
H	3.160240	-3.048795	0.492098
H	4.283005	-2.648530	1.806185
H	4.913962	-3.081604	0.198873
C	2.361726	1.831715	-0.644093
H	2.810746	2.762213	-0.300961
H	1.421813	1.681818	-0.123433
H	2.159915	1.892516	-1.712830
N	1.693198	-1.077081	0.061818
P	0.434631	-0.908928	-0.965501
N	-0.768010	-1.702708	-0.210359
C	-2.001317	-1.330617	-0.045411
N	-3.055270	-2.197299	0.038733
C	-4.223746	-1.505089	0.322356
C	-3.911755	-0.198094	0.403715
N	-2.539820	-0.080371	0.175287
C	-2.922141	-3.629432	-0.130469
H	-2.447976	-4.081760	0.742379
H	-2.312569	-3.840427	-1.007311
H	-3.913155	-4.056880	-0.265565
H	-5.167462	-2.008638	0.428159
H	-4.521532	0.663336	0.604494
C	0.260991	-0.352201	-2.468986
H	1.171323	0.098975	-2.869966
H	6.125064	-0.644296	0.420813
H	5.143423	1.793358	-0.404287
C	-1.805370	1.135681	0.341468
C	-1.881573	2.115198	-0.655424
C	-1.083543	1.326872	1.526309
C	-1.214695	3.319085	-0.436119
C	-0.427137	2.544429	1.700916
C	-0.496921	3.534871	0.731869
H	-1.257633	4.088428	-1.196734
H	0.135836	2.712032	2.610656
H	0.015265	4.476792	0.884635
C	-1.011946	0.261535	2.580952
H	-0.551182	0.653670	3.486963
H	-2.003993	-0.115825	2.837181
H	-0.418298	-0.588361	2.236964
C	-2.617912	1.867844	-1.937597
H	-3.651406	1.561530	-1.767785
H	-2.624043	2.767171	-2.552769
H	-2.122441	1.069981	-2.498155

$GH_1^{\ddagger}$ in implicit solvent diethyl ether

Number of atoms: 48

Energy: -1370.05633027

C	4.569655	-0.934953	0.789925
C	4.725423	0.182331	0.052959
N	3.259839	-1.356258	0.629544
C	2.577195	-0.494372	-0.201564
N	3.505566	0.463940	-0.551460
C	2.653814	-2.501968	1.270717
H	2.330246	-2.260933	2.285563
H	3.373642	-3.318754	1.308222
H	1.786685	-2.804417	0.688656
C	3.286497	1.569603	-1.464902
H	4.087449	2.292614	-1.320818
H	2.333409	2.051229	-1.252976
H	3.293597	1.232906	-2.502497
N	1.323265	-0.655384	-0.502044
P	0.362650	0.154150	-1.607904
N	-0.084955	1.614961	-0.909726
C	-0.875248	1.714243	0.115172
N	-1.053111	2.877762	0.824583
C	-1.966260	2.682041	1.850535
C	-2.373661	1.401252	1.796244
N	-1.708384	0.792057	0.727324
C	-0.346402	4.102434	0.524040
H	-0.499839	4.382597	-0.518068
H	0.724932	3.984958	0.696296
H	-0.728878	4.890236	1.169206
H	-2.248165	3.478073	2.515792
H	-3.090117	0.863532	2.388597
H	5.265848	-1.468970	1.411125
H	5.586013	0.807529	-0.101966
C	-1.004760	-0.680693	-2.152131
H	-0.791352	-1.703269	-2.470529
C	-1.970845	-0.539062	0.281451
C	-2.588352	-0.707131	-1.009931
C	-1.648394	-1.603197	1.121284
C	-2.999054	-2.050104	-1.296491
C	-2.018011	-2.891522	0.734921
C	-2.695182	-3.103711	-0.466356
H	-3.525616	-2.222461	-2.226984
H	-1.764724	-3.728595	1.372893
H	-2.993530	-4.107042	-0.745441
C	-0.926376	-1.388306	2.423288
H	-1.588694	-0.988711	3.195354
H	-0.528139	-2.333587	2.790775
H	-0.100706	-0.686400	2.309904
C	-3.423054	0.421162	-1.574377
H	-3.767962	0.153487	-2.572302
H	-4.297202	0.602852	-0.940013
H	-2.854097	1.343035	-1.658467

$GH_2^{\ddagger}$ in implicit solvent diethyl ether

Number of atoms: 48
Energy: -1370.06247946

C	-5.418345	-0.219266	0.087715
C	-4.810362	0.198058	1.217441
N	-4.450531	-0.813118	-0.704995
C	-3.241723	-0.766979	-0.074484
N	-3.470699	-0.146078	1.119684
C	-4.671768	-1.386216	-2.018268
H	-5.056025	-0.626268	-2.698832
H	-5.382528	-2.210392	-1.953772
H	-3.721110	-1.758097	-2.389922
C	-2.484146	0.044017	2.166671
H	-2.998380	0.077368	3.124813
H	-1.923685	0.963829	2.012608
H	-1.793493	-0.796880	2.168165
N	-2.136393	-1.301708	-0.546901
P	-0.630547	-0.743396	-0.574757
N	-0.580659	0.864919	-0.353401
C	0.599929	1.454708	-0.278971
N	0.712098	2.814535	-0.235994
C	2.041084	3.172753	-0.082411
C	2.757165	2.033437	-0.042958
N	1.877566	0.962996	-0.184024
C	-0.420527	3.714445	-0.286941
H	-1.057562	3.467030	-1.134699
H	-0.047883	4.730085	-0.398198
H	-1.013841	3.647407	0.626488
H	2.348193	4.201486	-0.028754
H	3.812656	1.867790	0.065022
H	-6.443868	-0.150347	-0.226898
H	-5.202030	0.698063	2.084578
C	0.798681	-1.445922	-0.743035
H	1.013698	-2.497990	-0.855411
C	2.393978	-0.421023	-0.031499
C	2.555332	-0.840940	1.334530
C	3.456607	-0.747983	-0.950113
C	3.520261	-1.786846	1.642122
C	4.391421	-1.695691	-0.585163
C	4.432911	-2.251473	0.697706
H	3.572749	-2.151492	2.663095
H	5.123281	-2.003847	-1.324994
H	5.187227	-2.980171	0.963328
C	3.375206	-0.233834	-2.354401
H	3.411012	0.857224	-2.415400
H	4.189680	-0.629168	-2.962808
H	2.422990	-0.539630	-2.806762
C	1.607367	-0.334580	2.380254
H	1.813700	-0.800378	3.344756
H	1.678000	0.749437	2.517045
H	0.567327	-0.554920	2.118157

H in implicit solvent diethyl ether
Number of atoms: 48
Energy: -1370.12684187

C	5.015284	-0.960105	0.725144
C	5.086309	0.168240	-0.007278
N	3.697081	-1.385698	0.702158
C	2.923105	-0.523919	-0.050270
N	3.807628	0.449575	-0.479577
C	3.158871	-2.554873	1.357147
H	2.354211	-2.274540	2.038358
H	3.956844	-3.036159	1.918653
H	2.757109	-3.258441	0.626139
C	3.494636	1.584154	-1.326777
H	4.255011	2.348095	-1.168768
H	2.523872	1.997179	-1.057613
H	3.484968	1.305798	-2.381529
N	1.650740	-0.699233	-0.210732
P	0.603285	0.093267	-1.278616
N	0.196807	1.555867	-0.500555
C	-0.869456	1.707734	0.216334
N	-1.271489	2.896665	0.780257
C	-2.546133	2.759767	1.318436
C	-2.935723	1.484821	1.133780
N	-1.891142	0.818138	0.492691
C	-0.513474	4.120781	0.661190
H	-0.421254	4.419397	-0.385258
H	0.488302	3.990467	1.070912
H	-1.027239	4.903185	1.215560
H	-3.049653	3.582874	1.792392
H	-3.840746	0.977978	1.413460
H	5.776433	-1.496319	1.262597
H	5.921854	0.804648	-0.236326
C	-0.848555	-0.989486	-0.883121
H	-0.518590	-2.008587	-0.729944
C	-1.909192	-0.554422	0.088728
C	-2.245954	-0.838417	-1.423058
C	-2.344873	-1.528222	1.108302
C	-3.062242	-2.042619	-1.637176
C	-2.959318	-2.665331	0.719017
C	-3.346856	-2.910979	-0.650156
H	-3.482779	-2.181892	-2.627279
H	-3.210483	-3.409270	1.465793
H	-3.939113	-3.792841	-0.863363
C	-1.988338	-1.254138	2.538357
H	-2.523165	-0.395013	2.949094
H	-2.215339	-2.120500	3.159677
H	-0.920869	-1.029959	2.628394
C	-2.570517	0.326299	-2.332031
H	-2.502820	0.002244	-3.373073
H	-3.585760	0.689544	-2.157634
H	-1.882878	1.160198	-2.210097

$HI_1^{\ddagger}$ in implicit solvent diethyl ether

Number of atoms: 48

Energy: -1370.12334188

C	4.943157	-0.986998	0.764207
C	5.049046	0.133889	0.024477
N	3.618081	-1.388872	0.718879
C	2.875503	-0.519865	-0.055264
N	3.784956	0.434025	-0.474972
C	3.045090	-2.541792	1.373781
H	2.230500	-2.240205	2.033611
H	3.822143	-3.030313	1.957896
H	2.647482	-3.246093	0.641306
C	3.507269	1.564623	-1.339433
H	4.285758	2.310840	-1.185942
H	2.545619	2.006571	-1.083275
H	3.498084	1.272460	-2.390375
N	1.603402	-0.673005	-0.239854
P	0.586302	0.137484	-1.320335
N	0.180408	1.600564	-0.543742
C	-0.835099	1.711196	0.246914
N	-1.249672	2.885413	0.829516
C	-2.469056	2.695429	1.467250
C	-2.813681	1.403473	1.329130
N	-1.788604	0.769128	0.617163
C	-0.561240	4.142332	0.644295
H	-0.572396	4.439384	-0.406430
H	0.476480	4.059022	0.967391
H	-1.061275	4.903215	1.239625
H	-2.974751	3.501510	1.967637
H	-3.679341	0.868980	1.670070
H	5.683758	-1.532706	1.320467
H	5.900491	0.752738	-0.193802
C	-0.890021	-0.926911	-0.942064
H	-0.557832	-1.957266	-0.884241
C	-1.793016	-0.572500	0.181375
C	-2.234973	-0.762699	-1.550403
C	-2.264558	-1.606349	1.065282
C	-3.061194	-1.918487	-1.733743
C	-2.770743	-2.766482	0.544581
C	-3.183206	-2.914247	-0.808555
H	-3.666531	-1.952784	-2.633716
H	-3.006966	-3.570555	1.232219
H	-3.756695	-3.797944	-1.063329
C	-2.158586	-1.411516	2.555630
H	-2.142635	-2.384088	3.048899
H	-1.248181	-0.873041	2.826626
H	-2.999965	-0.853121	2.973675
C	-2.583679	0.505339	-2.289188
H	-3.064526	1.235412	-1.633572
H	-1.708139	0.984451	-2.730135

H	-3.291693	0.279891	-3.088136

I in implicit solvent diethyl ether

Number of atoms: 48

Energy: -1370.14242038

C	-4.815068	-1.059180	-0.862244
C	-4.965001	0.008810	-0.055023
N	-3.476557	-1.415202	-0.833188
C	-2.768683	-0.569249	-0.002058
N	-3.714954	0.323086	0.470469
C	-2.860048	-2.508493	-1.547890
H	-2.062981	-2.141237	-2.195804
H	-3.619727	-3.000563	-2.151695
H	-2.428205	-3.230286	-0.852866
C	-3.485464	1.402308	1.411210
H	-4.280906	2.136641	1.289643
H	-2.531637	1.884585	1.203537
H	-3.488754	1.043155	2.441351
N	-1.493547	-0.689227	0.181169
P	-0.509338	0.083555	1.320164
N	-0.188033	1.624412	0.654477
C	0.757069	1.804827	-0.204031
N	1.118219	3.024558	-0.721770
C	2.241912	2.888171	-1.523764
C	2.586723	1.590301	-1.547676
N	1.659075	0.888785	-0.759306
C	0.448532	4.256645	-0.373338
H	0.496952	4.428579	0.703292
H	-0.600694	4.221207	-0.669213
H	0.939561	5.076872	-0.892164
H	2.697463	3.735145	-2.004787
H	3.410358	1.102320	-2.028576
H	-5.532912	-1.595070	-1.456607
H	-5.839247	0.582436	0.194513
C	1.041232	-0.862073	0.872384
C	1.683264	-0.469220	-0.429182
C	2.168380	-0.939661	1.866997
C	2.268189	-1.431666	-1.202245
C	2.993289	-2.010809	1.760206
C	2.639774	-2.694727	-0.624134
C	2.988383	-2.948057	0.675025
H	3.733973	-2.171813	2.538729
H	2.810707	-3.498506	-1.334161
H	3.413745	-3.925530	0.879646
H	0.662815	-1.874280	0.729586
C	2.368862	0.136723	2.885342
H	2.657432	1.075621	2.402404
H	1.449444	0.341190	3.439389
H	3.158143	-0.127947	3.590122
C	2.586239	-1.250824	-2.667031
H	2.384166	-2.188378	-3.190300

H	1.977076	-0.474382	-3.127946
H	3.638384	-1.010912	-2.848805

3.4 Steric maps of [3a]+ and [3b]+

Computation of percent buried volume (%Vbur) values and steric maps of [**3a**]I and [**3b**]OTf was performed *via* the SambVca 2.1 web application.[14] The spheres are centered at the phosphorus-bound carbon atom. Bond radii are scaled by 1.17, the sphere radius is set to 3.5 Å, mesh spacing for numerical integration is set to 0.10 Å, H atoms are not included in the calculations. The steric maps are depicted in **Figure S 97** ([**3a**]+) and **Figure S 98** ([**3b**]+).

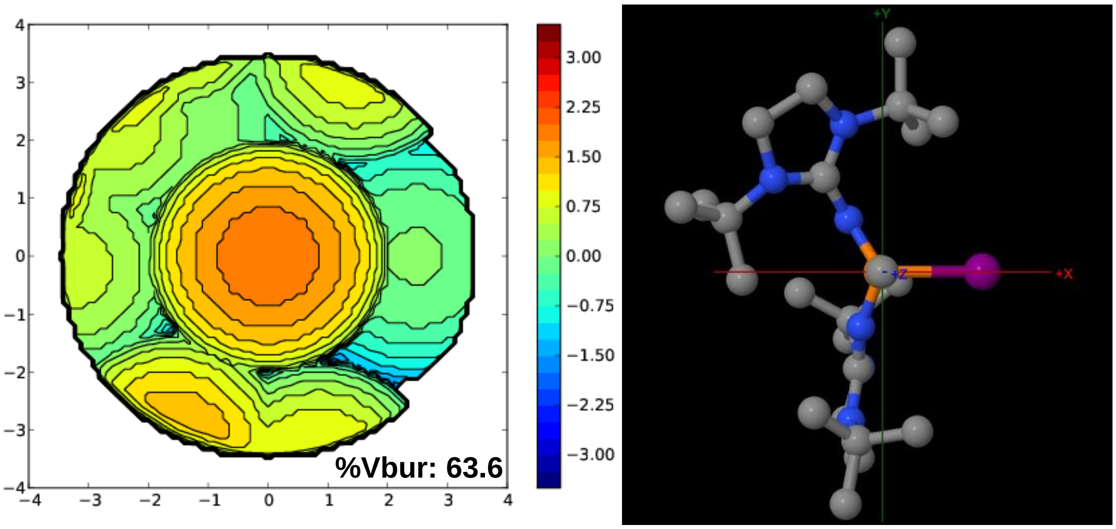

Figure S 97: Steric map of [**3a**]+ (left) and the corresponding alignment of the molecule in the ball-and-stick model (right).

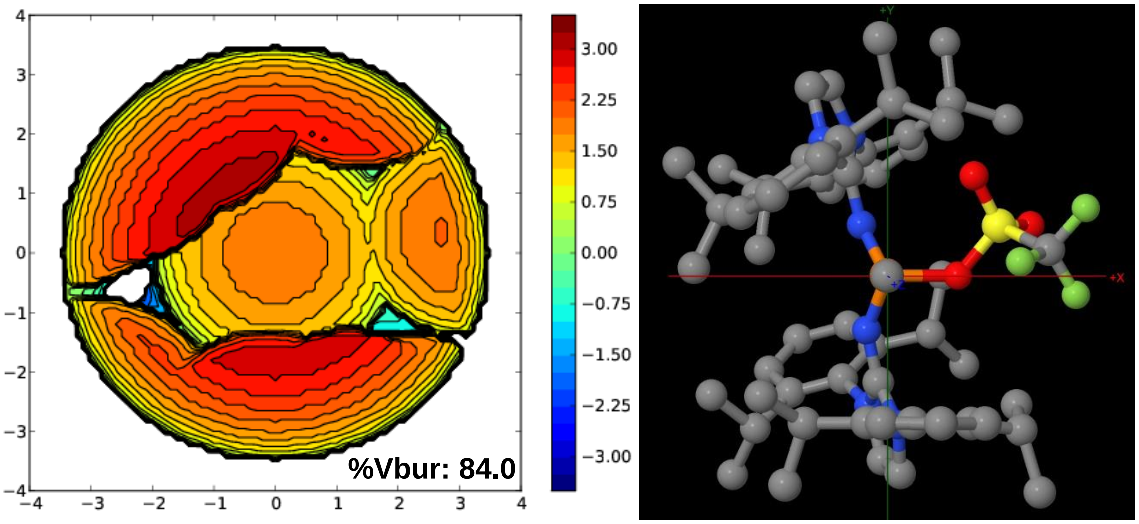

Figure S 98: Steric map of [**3b**]+ (left) and the corresponding alignment of the molecule in the ball-and-stick model (right).

3.5 Atomic charges and electrostatic surface potential plots of model compounds of phosphonium cations [3a]$^+$, [3b]$^+$ and [4a]$^+$/[4b]$^+$

Model systems (Dipp and *t*Bu groups substituted with methyl groups) of the phosphonium cations [**3a**]$^+$, [**3b**]$^+$ and [**4a**]$^+$/[**4b**]$^+$ have been calculated at B3LYP+D3/def2-TZVP DFT level. The obtained geometrical data along with the NBO and Mulliken partial charges are listed in Table S4-S6 and the corresponding electrostatic surface potential plots are depicted in Figure S99. The NBO partial charges of the phosphorus atoms in [**3a**]$^+$ and [**3b**]$^+$ are 1.842*e* and 2.238*e*, respectively, suggesting a higher electrophilicity for [**3b**]$^+$. This finding is in line with the assumption of electron transfer to the P atom as an alternative reaction pathway for deprotonation of the methyl group attached to phosphorus. Note that the hydrogen atoms of the methyl group at the phosphorus atom feature higher positive partial charges (Mulliken and NBO analysis) than other hydrogen atoms in the molecule, which is a direct indication of higher acidity.

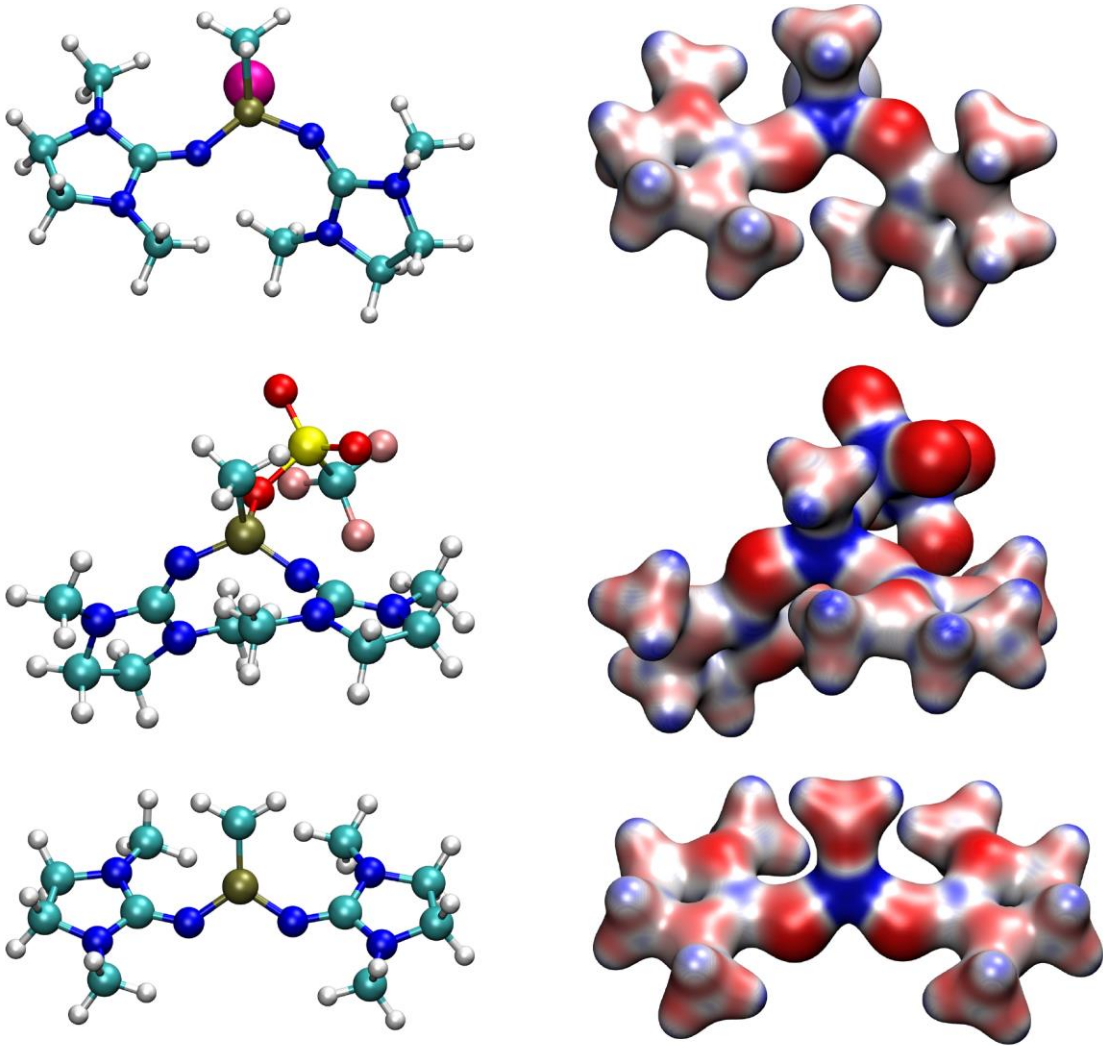

Figure S99: Minimum structures (left) and electrostatic surface potential plots (right) obtained at B3LYP+D3/def2-TZVP level for models of [**3a**]$^+$ (top), [**3b**]$^+$ (center) and [**4a**]$^+$/[**4b**]$^+$ (bottom). In all cases the pronounced positive charge at the P atom is visible. The sum of angles of the N_2PC unit of the optimized geometries are 334.2° ([**3a**]$^+$), 344.0° ([**3b**]$^+$) and 359.8° ([**4a**]$^+$).

Table S4: Atomic coordinates in Å, NBO and Mulliken charges in units of the elementary charge *e* of model [**3a**]$^{+}$ obtained at B3LYP+D3/def2-TZVP level according to an NBO and Mulliken population analysis.

	x	y	z	q_NBO	q_Mull
N	-2.544735	-2.363652	-0.279469	-0.3489	-0.092027
C	-2.249029	-1.089762	0.068739	0.65582	0.300559
N	-3.394986	-0.388984	0.236752	-0.36179	-0.116333
C	-1.593811	-3.450751	-0.25186	-0.40409	-0.286778
H	-0.592133	-3.053108	-0.392094	0.2312	0.160885
H	-1.821027	-4.149018	-1.057584	0.21159	0.146101
H	-1.633943	-3.98874	0.701324	0.19751	0.133891
C	-3.531865	1.05005	0.236008	-0.40229	-0.276111
H	-3.739713	1.423016	-0.771877	0.20184	0.144472
H	-2.62981	1.527919	0.5963	0.21885	0.140162
H	-4.358206	1.328081	0.890452	0.21404	0.151483
N	-1.006462	-0.704105	0.193963	-1.04364	-0.412679
**P**	-0.009916	0.335819	0.826302	**1.84232**	**0.464506**
N	1.487963	-0.160336	0.927894	-1.0203	-0.404814
C	2.415934	-0.711727	0.186627	0.65167	0.284689
N	3.62894	-1.003067	0.71322	-0.3497	-0.095382
N	2.3873	-1.093205	-1.11363	-0.36653	-0.129844
C	3.919475	-1.005072	2.127786	-0.40341	-0.27728
H	3.759463	-1.996178	2.566329	0.19537	0.130335
H	3.274999	-0.287273	2.627808	0.23179	0.156378
H	4.960362	-0.719272	2.280158	0.21175	0.145181
C	1.441012	-0.670681	-2.124131	-0.40755	-0.296971
H	1.743074	0.274134	-2.584728	0.2008	0.147485
H	0.448071	-0.565324	-1.702044	0.22823	0.155146
H	1.400637	-1.439027	-2.896592	0.21278	0.149041
C	-4.547025	-1.196056	-0.17511	-0.20929	-0.101737
H	-4.87241	-0.887865	-1.173891	0.19662	0.117757
H	-5.375171	-1.066761	0.519287	0.21914	0.141911
C	-3.978035	-2.614187	-0.173501	-0.21083	-0.097679
H	-4.197489	-3.142708	0.760057	0.19533	0.115114
H	-4.333292	-3.216593	-1.007756	0.21907	0.141494
C	4.424863	-1.794717	-0.217057	-0.21056	-0.094683
H	4.381239	-2.854462	0.05673	0.1928	0.111635
H	5.465295	-1.474587	-0.211401	0.21869	0.140927
C	3.721618	-1.51862	-1.544048	-0.20838	-0.104247
H	4.205901	-0.709965	-2.100807	0.19577	0.116764
H	3.657261	-2.395953	-2.184947	0.21697	0.138861
C	-0.466581	0.855973	2.484084	-0.98594	-0.357637
**H**	-0.467394	-0.033684	3.117409	**0.25867**	**0.159004**

**H**	0.270169	1.567431	2.852757	**0.26403**	**0.168784**
**H**	-1.455499	1.310194	2.48676	**0.25179**	**0.162532**
I	0.015337	2.497721	-0.457029	-0.20123	-0.180897

Table S5: Atomic coordinates in Å, NBO and Mulliken charges in units of the elementary charge *e* of model [**3b**]$^+$ obtained at B3LYP+D3/def2-TZVP level according to an NBO and Mulliken population analysis.

	x	y	z	q_NBO	q_Mull
N	-1.269285	2.977375	-1.088003	-0.34767	-0.095043
C	-0.303985	2.456974	-0.30678	0.66165	0.295318
N	0.449537	3.453022	0.217992	-0.36601	-0.119591
C	-2.407461	2.238474	-1.585517	-0.40228	-0.278098
H	-2.136984	1.193206	-1.689796	0.23088	0.156055
H	-2.689923	2.63578	-2.560522	0.21106	0.14752
H	-3.26237	2.320989	-0.90826	0.20269	0.137284
C	1.78542	3.317045	0.754041	-0.40184	-0.296248
H	2.51916	3.706572	0.041426	0.20391	0.144441
H	2.019722	2.275391	0.947541	0.21641	0.144489
H	1.874149	3.875204	1.687	0.21268	0.155883
N	-0.163754	1.16368	-0.13549	-1.04715	-0.370975
**P**	0.366075	0.163023	0.948229	**2.23846**	**0.509884**
N	1.806686	-0.441987	0.838242	-1.03918	-0.384582
C	2.628435	-0.955194	-0.049261	0.65801	0.309752
N	2.393667	-1.298446	-1.335193	-0.36283	-0.113803
N	3.912721	-1.219686	0.266018	-0.34574	-0.093416
C	1.091732	-1.463707	-1.94703	-0.41181	-0.301047
H	1.223276	-1.457567	-3.027996	0.22054	0.153231
H	0.441243	-0.63399	-1.687385	0.22587	0.155287
H	0.624419	-2.408854	-1.657144	0.20327	0.142676
C	4.58035	-0.743735	1.454373	-0.40366	-0.28044
H	3.843034	-0.566401	2.232697	0.23182	0.157314
H	5.125211	0.185512	1.256455	0.19674	0.132459
H	5.288647	-1.497825	1.797784	0.21366	0.149061
C	0.096352	0.667533	2.638346	-1.01746	-0.389137
**H**	-0.92274	1.030494	2.766021	**0.26312**	**0.174513**
C	0.047828	4.745801	-0.34309	-0.21073	-0.106518
H	0.779493	5.06069	-1.09329	0.199	0.119722
H	-0.004986	5.506684	0.433696	0.21903	0.14175
C	-1.312844	4.431738	-0.967064	-0.21179	-0.097668
H	-2.140958	4.72332	-0.31392	0.20083	0.118847
H	-1.453057	4.900982	-1.939354	0.21916	0.1414
C	3.546878	-2.019253	-1.882593	-0.20773	-0.106075
H	3.742836	-1.709223	-2.906929	0.21874	0.142273
H	3.350394	-3.095786	-1.870289	0.1981	0.120826
C	4.665058	-1.633516	-0.914134	-0.21035	-0.097213
H	5.331049	-2.462211	-0.680559	0.22016	0.143189
H	5.262688	-0.796821	-1.290331	0.19501	0.114181
**H**	0.278044	-0.176818	3.302952	**0.26975**	**0.176116**
**H**	0.796684	1.466055	2.881894	**0.26606**	**0.173567**

O	-0.639044	-1.205199	0.816679	-0.96838	-0.352912
**S**	-2.187586	-1.357083	1.041629	**2.3058**	**0.822773**
O	-2.408643	-2.372753	2.016515	-0.88134	-0.402518
O	-2.831707	-0.085209	1.126331	-0.89808	-0.414824
C	-2.625745	-2.102581	-0.611365	0.70948	0.4619
F	-3.935223	-2.328509	-0.625596	-0.28906	-0.140979
F	-1.975151	-3.24813	-0.785322	-0.29143	-0.146468
F	-2.312149	-1.265284	-1.597071	-0.29738	-0.154158

Table S6: Atomic coordinates in Å, NBO and Mulliken charges in units of the elementary charge *e* of model [**4a**]$^+$/[**4b**]$^+$ obtained at B3LYP+D3/def2-TZVP level according to an NBO and Mulliken population analysis.

	x	y	z	q_NBO	q_Mull
N	3.530027	1.138909	-0.219445	-0.34576	-0.086705
C	2.566662	0.201271	-0.179162	0.65319	0.30156
N	3.116656	-1.030783	-0.153833	-0.36159	-0.108593
C	3.310934	2.550476	-0.005151	-0.40391	-0.27982
H	2.270395	2.784748	-0.213703	0.23447	0.161822
H	3.950139	3.123394	-0.677587	0.21177	0.145835
H	3.543754	2.831563	1.026878	0.19897	0.134705
C	2.429007	-2.270236	-0.444578	-0.40271	-0.2928
H	2.958878	-2.795373	-1.241882	0.21251	0.149896
H	1.415428	-2.071846	-0.78347	0.21815	0.155001
H	2.386721	-2.914952	0.434979	0.21061	0.151204
N	1.285741	0.512299	-0.213634	-0.99518	-0.428534
**P**	0.013907	-0.067482	0.506075	**1.92769**	**0.514345**
N	-1.288361	0.471951	-0.181308	-0.99679	-0.422876
C	-2.572694	0.189716	-0.139764	0.65398	0.320307
N	-3.512887	1.145675	-0.235854	-0.34177	-0.083405
N	-3.155098	-1.030011	-0.052194	-0.36362	-0.132326
C	-3.264046	2.560593	-0.08121	-0.40451	-0.280612
H	-3.892959	3.116145	-0.777212	0.21247	0.146146
H	-2.21915	2.764534	-0.299909	0.23465	0.16285
H	-3.489847	2.889497	0.938095	0.20006	0.135869
C	-2.499336	-2.280728	-0.373219	-0.40857	-0.29069
H	-1.4777	-2.286553	-0.004562	0.22136	0.147919
H	-2.496913	-2.461267	-1.453251	0.19936	0.142704
H	-3.038368	-3.091135	0.116206	0.21873	0.154167
C	0.048649	-0.99396	1.848506	-1.13401	-0.519541
**H**	0.992361	-1.249686	2.306815	**0.25787**	**0.177375**
C	4.572751	-0.946502	-0.297977	-0.20915	-0.113563
H	4.856155	-1.252158	-1.30901	0.20176	0.125057
H	5.072951	-1.597424	0.417073	0.21832	0.142225
C	4.850374	0.539464	-0.046963	-0.21212	-0.094652
H	5.209744	0.724052	0.969712	0.20071	0.118287
H	5.565706	0.960499	-0.751707	0.21928	0.141035
C	-4.849505	0.58236	-0.066881	-0.21237	-0.093861
H	-5.546798	1.00822	-0.786294	0.22001	0.142002
H	-5.214537	0.791286	0.943352	0.20059	0.117737

C	-4.597977	-0.910138	-0.289501	-0.20993	-0.105412
H	-5.156391	-1.544663	0.395759	0.21958	0.142024
H	-4.826709	-1.211543	-1.316301	0.19704	0.119261
**H**	-0.872922	-1.295278	2.324157	**0.25884**	**0.184057**

4 References

1 M. D. Böhme, T. Eder, M. B. Röthel, P. D. Dutschke, L. F. B. Wilm, F. E. Hahn and F. Dielmann, *Angew. Chem. Int. Ed.*, **2022**, *61*, e202202190.

2 P. Löwe, M. Feldt, M. A. Wünsche, L. F. B. Wilm and F. Dielmann, *J. Am. Chem. Soc.*, **2020**, *142*, 9818–9826.

3 O. V. Dolomanov, L. J. Bourhis, R. J. Gildea, J. A. K. Howard and H. Puschmann, *J. Appl. Crystallogr.*, **2009**, *42*, 339–341.

4 L. Palatinus and G. Chapuis, *J. Appl. Crystallogr.*, **2007**, *40*, 786–790.

5 M. C. Burla, R. Caliandro, M. Camalli, B. Carrozzini, G. L. Cascarano, L. de Caro, C. Giacovazzo, G. Polidori and R. Spagna, *J. Appl. Crystallogr.*, **2005**, *38*, 381–388.

6 G. M. Sheldrick, *Acta Crystallogr. A*, **2015**, *71*, 3–8.

7 G. M. Sheldrick, *Acta Crystallogr. C*, **2015**, *71*, 3–8.

8 G. M. Sheldrick, *Acta Crystallogr. A*, **2008**, *64*, 112–122.

9 T. Vreven, J. Throssell, J. A. Montgomery, Jr., J. E. Peralta, F. Oligaro, M. Bearpark, J. J. Heyd, E. N. Brothers, K. N. Kudin, V. N. Staroverov, T. A. Keith, R. Kobayashi, J. Normand, K. Raghavachari, A. P. Rendell, J. C. Burant, S. S. Iyengar, J. Tomasi, M. Cossi, J. M. Millam., M. Klene, C. Adamo, R. Cammi, J. W. Ochterski, R. L. Martin, K. Morokum, O. Farkas, J. B. Foresman and D. J. Fox, *Gaussian 16*, Gaussian Inc., Wallingford CT, **2016**.

10 A. D. Becke, *J. Chem. Phys.*, **1993**, *98*, 1372–1377.

11 F. Weigend and R. Ahlrichs, *Phys. Chem. Chem. Phys.*, **2005**, *7*, 3297–3305.

12 S. Grimme, S. Ehrlich and L. Goerigk, *J. Comput. Chem.*, **2011**, *32*, 1456–1465.

13 A. V. Marenich, C. J. Cramer and D. G. Truhlar, *J. Phys. Chem. B*, **2009**, *113*, 6378–6396.

14 L. Falivene, Z. Cao, A. Petta, L. Serra, A. Poater, R. Oliva, V. Scarano and L. Cavallo, *Nat. Chem.*, **2019**, *11*, 872–879.
